# Discordant hepatic fatty acid oxidation and triglyceride hydrolysis leads to liver disease

**DOI:** 10.1172/jci.insight.135626

**Published:** 2021-01-25

**Authors:** Ebru S. Selen, Joseph Choi, Michael J. Wolfgang

**Affiliations:** 1 Department of Biological Chemistry and; 2 Department of Pharmacology and Molecular Sciences, Johns Hopkins University School of Medicine, Baltimore, Maryland, USA.

**Keywords:** Metabolism, Bioenergetics, Fatty acid oxidation, Mitochondria

## Abstract

To extract energy from stored lipids, fatty acids must first be liberated from triglyceride before their β-oxidation in mitochondria in a coordinated and stepwise manner. To determine the independent and interdependent roles of hepatic triglyceride hydrolysis and fatty acid oxidation, mice were generated with a liver-specific defect in triglyceride hydrolysis (Atgl^L–/–^), fatty acid oxidation (Cpt2^L–/–^), or both (double knockout). The loss of either gene resulted in the compensatory increase in the other, demonstrating their coordination. The loss of individual components of fatty acid catabolism (carnitine palmitoyl transferase 2 [Cpt2], adipose triglyceride lipase [Atgl], and Pparα) resulted in largely independent effects on hepatocyte morphology, intermediary metabolism, and gene expression in response to fasting. However, high-fat feeding revealed the interdependent role of Atgl and Cpt2, as the loss of only one of the genes resulted in steatosis (fatty liver) but the loss of both components resulted in significant steatohepatitis (inflammation and fibrosis). Lipolysis and β-oxidation are intimately linked within a continuous pathway, and disruption of their coordination leads to unique cellular and molecular phenotypes that ultimately result in liver disease.

## Graphical abstract

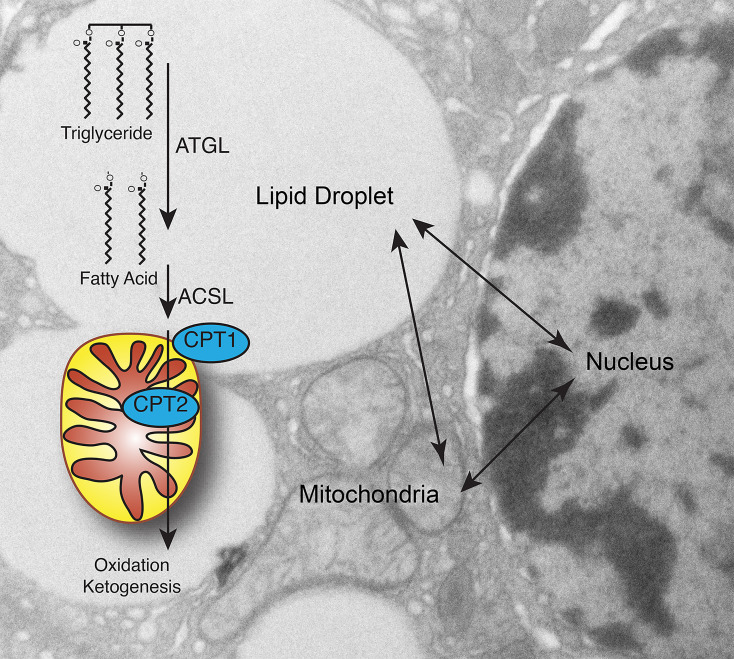

## Introduction

The metabolism, storage, and flux of lipids in the liver play a central role in starvation, diet-induced obesity, diabetes, and nonalcoholic steatohepatitis (NASH). The liver has a uniquely large dynamic range for lipid metabolism as it switches between a primary site of de novo lipogenesis to a primary site of lipid oxidation. The balance of lipid synthesis, uptake, export, and oxidation plays an essential role in the progression and pathogenesis of the metabolic syndrome and is particularly important for the growing incidence of fatty liver and NASH. However, the mechanisms governing the shift from normal metabolic physiology to pathophysiology are poorly understood with respect to the role of lipid metabolism.

Fatty acids that have been made de novo or incorporated from the diet are stored as triglyceride (TG) in lipid droplets and are mobilized during times of energy deficit to provide fatty acids for mitochondrial oxidative metabolism. Under most conditions, the liberation of fatty acids from TGs is regulated by the TG hydrolase adipose TG lipase (Atgl; also known as Pnpla2, desnutrin) (1, 2). Atgl is the first rate-setting enzyme in TG hydrolysis (1–3), and mutations in either Atgl or its coactivator Cgi-58 result in neutral lipid storage disease in humans (4, 5). These disorders as well as the complete loss of Atgl in mice result in defects in mitochondrial fatty acid oxidation. The inability to mobilize TGs starves mitochondria of fatty acids and limits oxidative metabolism. Additionally, defects in TG hydrolysis have been shown to exhibit significant transcriptional deficits (3, 6–10). That is, liberation of fatty acids from lipid droplets is an important regulator of Pparα-mediated regulation of fatty acid oxidative transcriptional programing. Therefore, Atgl is important both for providing the substrate for fatty acid oxidation and for coordinating the transcriptional program required for sustaining the oxidation of fatty acids.

Fatty acids are oxidized in mitochondria to provide hepatocytes with ATP and NADH to facilitate gluconeogenesis and generate acetyl-CoA, the carbon substrate for ketogenesis. This enables the liver to buffer blood glucose and provide alternative fuel (ketone bodies) for highly oxidative tissues during food deprivation. The significance of fatty acid oxidation in many biological processes is made evident from multiple mutations in this pathway that cause human disease (11). Long-chain fatty acid β-oxidation is governed by the regulated translocation of activated fatty acids (acyl-CoAs) from the cytoplasm to the matrix of the mitochondria. This is mediated by successive acyltransferases carnitine palmitoyl transferases 1 and 2 (Cpt1 and Cpt2). Mutations in CPT2 result in metabolic disease, the most severe presenting as hypothermia, cardiomegaly, hepatomegaly, and hypoglycemia in the first days of life (OMIM #600650). It is evident that fatty acid oxidation is an important and central catabolic process and that its dysregulation can lead to liver pathology and multisystemic dysfunction.

To extract energy from stored lipids, fatty acids must first be liberated from TGs before mitochondrial β-oxidation in a coordinated and stepwise manner. Within this metabolic continuum, we have generated single and double KO mouse models within key lipid catabolism genes (Atgl, Cpt2, and Pparα) to interrogate the independent and interdependent roles of the genes in this pathway. Surprisingly, we show that defects in these genes result in independent effects on hepatocyte morphology, intermediary metabolism, and gene expression. However, high-fat feeding revealed the interdependent role of Atgl and Cpt2 as the loss of only one of the genes resulted in steatosis; however, loss of both components resulted in significant steatohepatitis.

## Results

### Reciprocal regulation of hepatic TG hydrolysis and fatty acid oxidation.

**Figure 1:**
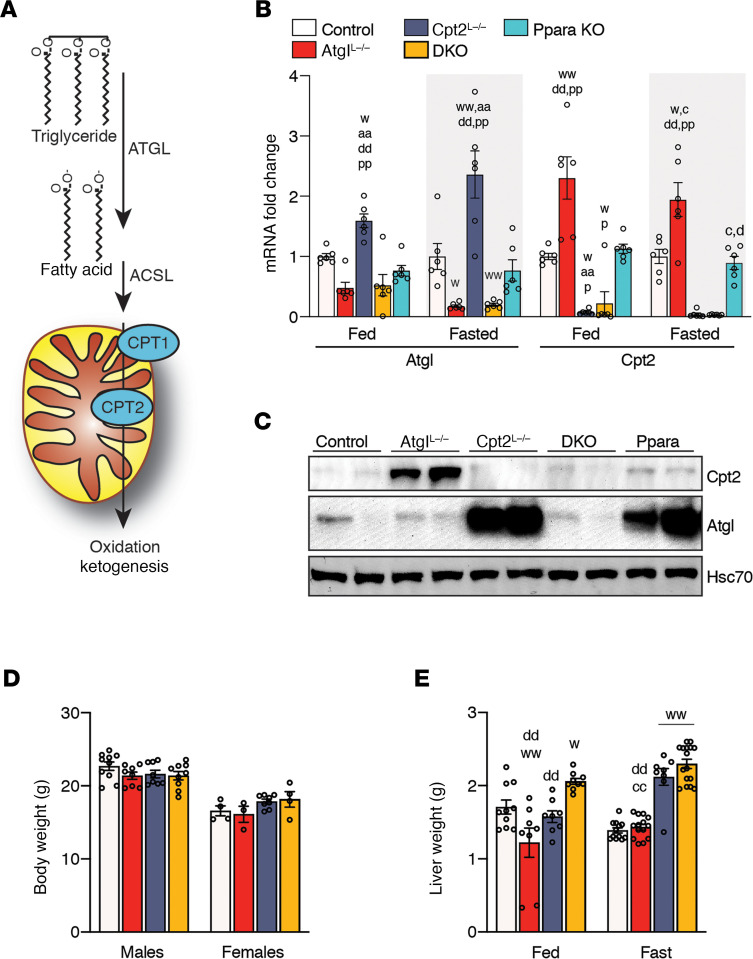
Characterization of mice with a liver-specific double KO of Cpt2 and Atgl. (**A**) Schematic of the relationship between TG hydrolysis and fatty acid β-oxidation. (**B**) mRNA of Atgl and Cpt2 under fed and fasted conditions in the livers of control, Atgl-Cpt2^L–/–^ (DKO), Atgl^L–/–^ (Atgl), Cpt2^L–/–^ (Cpt2), and Pparα-KO mice (*n* = 6/genotype). (**C**) Western blot for Atgl and Cpt2 in livers of control, Atgl, Cpt2, DKO, and Pparα-KO mice. (**D**) Body weights of 9-week-old male and female mice fed a chow diet (males, *n* = 8–16; females, *n* = 8–10). (**E**) Liver weights of male mice under fed and fasted conditions (*n* = 10–16). ACSL, acyl-CoA synthetase long chain.

Fatty acid oxidation and lipid droplet hydrolysis are intimately linked in a metabolic continuum. This is particularly important during times of nutrient deprivation in which fatty acids must first be released from stored TGs before they are available for oxidation (Figure 1A). To understand the independent and interdependent roles of TG hydrolysis and fatty acid oxidation, we generated mice with a liver-specific KO of Atgl, the first step in TG hydrolysis (Atgl^L–/–^); mice with a liver-specific KO of Cpt2, an obligate step in mitochondrial fatty acid β-oxidation (Cpt2^L–/–^); mice with a loss of both genes (double KO, DKO); and mice with a loss of Pparα, the main transcriptional regulator of hepatic lipid catabolism. Previously, we performed microarray analysis on livers from Cpt2^L–/–^ mice following a 24-hour fast and demonstrated an induction of *Atgl* mRNA (12). Here, we confirmed this by quantitative PCR and showed that *Atgl* was induced in the liver by the loss of Cpt2 (Figure 1B). At the protein level, the loss of Cpt2 induced a dramatic upregulation of Atgl by fasting, in part due to its stabilization on the abundant lipid droplets that accumulate in Cpt2^L–/–^ livers upon fasting (Figure 1C). This induction was similarly observed in lipid-laden, PPARα-KO fasted livers (Figure 1C). Additionally, Cpt2 was induced at the mRNA and protein level in Atgl^L–/–^ fasted liver (Figure 1, B and C). These data suggest a reciprocal regulation of the machinery of lipid hydrolysis and fatty acid oxidation in hepatocytes.

**Figure 2:**
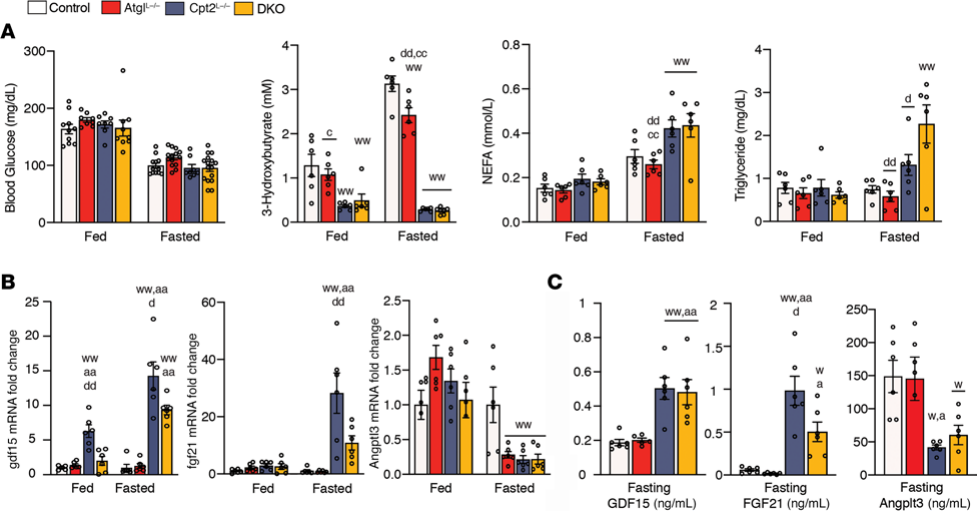
Characterization of serum from mice with a liver-specific double KO of Cpt2 and Atgl. (**A**) Serum metabolites in mice under fed and fasted conditions (*n* = 6). (**B**) mRNA of hepatokines in livers of mice in fed and fasted states were measured by quantitative reverse-transcription-PCR (*n* = 6). (**C**) ELISA of hepatokine levels in ng/mL in serum of fasted mice (*n* = 6). One- or 2-way ANOVA followed by Tukey’s multiple-comparison test were performed where appropriate to detect significance between genotypes. Single letter denotes *P* < 0.05. Double letters denote *P* < 0.01. Letters w (control), a (Atgl), c (Cpt2), d (DKO), and p (Pparα) represent significance between the genotypes. Data are shown as mean ± SEM.

Next, we determined the physiological consequence of losing hepatic fatty acid oxidation and lipid droplet hydrolysis. Body weights were not significantly different between male or female control, Atgl^L-/^, Cpt2^L–/–^, or DKO mice (Figure 1D). As expected, Cpt2^L–/–^ and DKO livers were considerably enlarged following a 24-hour fast with minimal effects on other tissues (Figure 1E). Even though fatty acid oxidation is required for hepatic gluconeogenesis, all genotypes were able to maintain systemic blood glucose concentrations following a 24-hour fast (Figure 2A). Although circulating glucose concentrations were normal in all genotypes, Cpt2^L–/–^ and DKO mice failed to induce the generation of ketone bodies upon fasting (Figure 2A). Concomitant with this observation, Cpt2^L–/–^ and DKO mice exhibited elevated serum nonesterified fatty acid (NEFA) and TG following a fast (Figure 2A).

Previously, we showed that the loss of hepatic fatty acid oxidation results in dramatic changes in hepatokine expression (12, 13). Gdf15 mRNA and serum concentrations increased and Angptl3 concentrations decreased in Cpt2^L–/–^ and DKO mice during the fed state and potentiated following fasting (Figure 2, B and C). Fgf21 mRNA and serum concentrations increased in Cpt2^L–/–^ mice following fasting, but Atgl loss in DKO mice suppressed the induction of Fgf21 (Figure 2, B and C). Atgl-mediated lipolysis has been linked to Pparα directed gene expression and Fgf21 is particularly sensitive to Pparα (14). These data suggest that the loss of Atgl could partially suppress the transcriptional response in Cpt2^L–/–^ mice.

### Specific defects in lipid catabolism generate unique cellular phenotypes.

**Figure 3:**
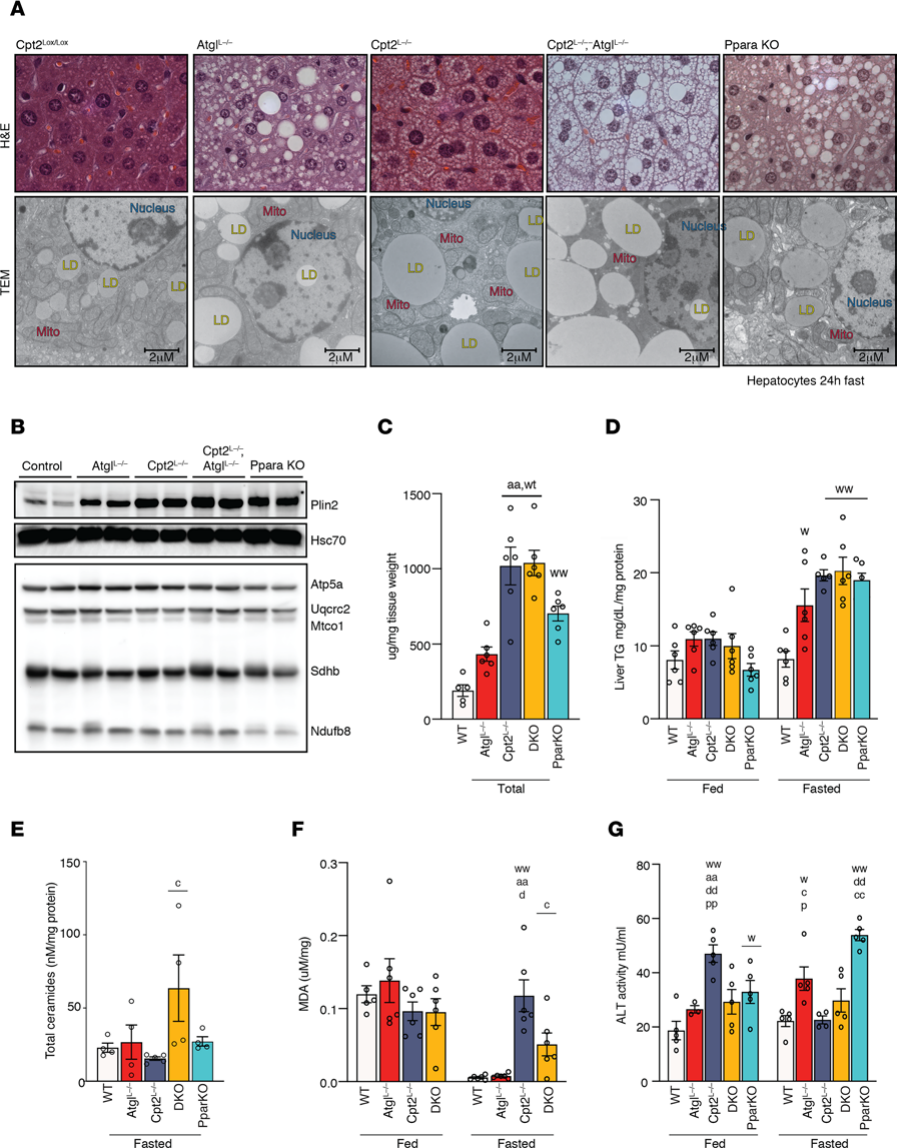
Loss of individual components of fatty acid catabolism results in unique hepatocyte morphology. (**A**) H&E staining and transmission electron micrographs of livers from 24-hour fasted control, Atgl^L–/–^, Cpt2^L–/–^, DKO, and Pparα-KO mice. (**B**) Western blot of oxidative phosphorylation (OXPHOS) complexes in livers of all genotypes. (**C**) Total liver fatty acid measurements from all genotypes (*n* = 6). (**D**) Triglyceride content of liver from mice under fed and fasting conditions (*n* = 6). (**E**) Total ceramides from all genotypes (*n* = 4). (**F**) Lipid peroxidation in livers of fed and fasted mice measured using thiobarbituric acid reactive substances (TBARS) assay (*n* = 6). (**G**) Liver damage assessed by serum ALT activity of fed and 24-hour-fasted animals and represented as fold changes relative to control (*n* = 5). One- or 2-way ANOVA followed by Tukey’s multiple-comparison test were performed where appropriate to detect significance between genotypes. Single letter denotes *P* < 0.05. Double letters denote *P* < 0.01. Letters w (control), a (Atgl), c (Cpt2), d (DKO), and p (Pparα) represent significance between the genotypes. Data are shown as mean ± SEM. Females were used in **A** and males in **B**–**G**. MDA, malondialdehyde.

To understand the independent and interdependent contributions of Atgl and Cpt2 we compared the liver histology of fasted control, Atgl^L–/–^, Cpt2^L–/–^, DKO, and Pparα-KO mice. Surprisingly, although defects in any of these lipid catabolism genes increased lipid content over control mice, the lipid droplet morphology between these models was unique. Previous reports have shown that Atgl^L–/–^ mice exhibited large periportal lipid droplets (9, 15, 16). Additionally, we previously reported that Cpt2^L–/–^ mice exhibited small uniformly distributed lipid droplets and Pparα-KO mice exhibited an intermediate lipid droplet size (12). Surprisingly, DKO mice exhibited both large and small lipid droplets, suggesting that the induction of Atgl in Cpt2^L–/–^ mice served to efficiently eliminate large lipid droplets (Figure 3A). Atgl has been shown to be preferentially associated with large lipid droplets, and the accumulation of small lipid droplets has been suggested to arise from a defect in lipophagy (15, 17). Intriguingly, transmission electron micrographs (TEMs) demonstrated the loss of Atgl was associated with an abundance of nuclear lipid droplets somewhat independent of total lipid concentration (Figure 3A). Although the mechanism by which nuclear lipid droplets are formed is not clear, these data suggest that Atgl may be required for nuclear TG hydrolysis in hepatocytes. The dual loss of Atgl and Cpt2 in DKO mice demonstrated physical stress on the nucleus by both the growth of intranuclear lipid droplets and the incursion of extranuclear lipid droplets distorting the nuclear structure.

Analysis of mitochondrial proteins demonstrated normal mitochondrial content whereas Perilipin2 (Plin2) expression, a protein stabilized by lipid droplet association, was induced concomitant with lipid droplet accumulation in the different models (Figure 3B). The analysis of total liver fatty acid content (Figure 3C) and TG content (Figure 3D) demonstrated, in agreement with the histology, that Pparα-KO, Cpt2^L–/–^, and DKO mice exhibited the most lipid accumulation, whereas Atgl^L–/–^ mice exhibited an intermediary lipid accumulation. Concomitant with the increased hepatic fatty acids, DKO mice exhibited increased ceramides (Figure 3E). Consistent with the role of lipid droplets as a cytoprotective organelle, the loss of Atgl in Cpt2^L–/–^ mice improved hepatic lipid peroxidation (Figure 3F). Given the dramatic changes in tissue architecture and induction of ceramide production, we were surprised to see that serum alanine aminotransferase (ALT) activity was largely unchanged in the different models following a 24-hour fast (Figure 3G). These data show that the loss of fatty acid catabolism at different points results in unique cellular and physiological effects.

### Specific defects in lipid catabolism generate unique molecular and metabolic phenotypes.

**Figure 4:**
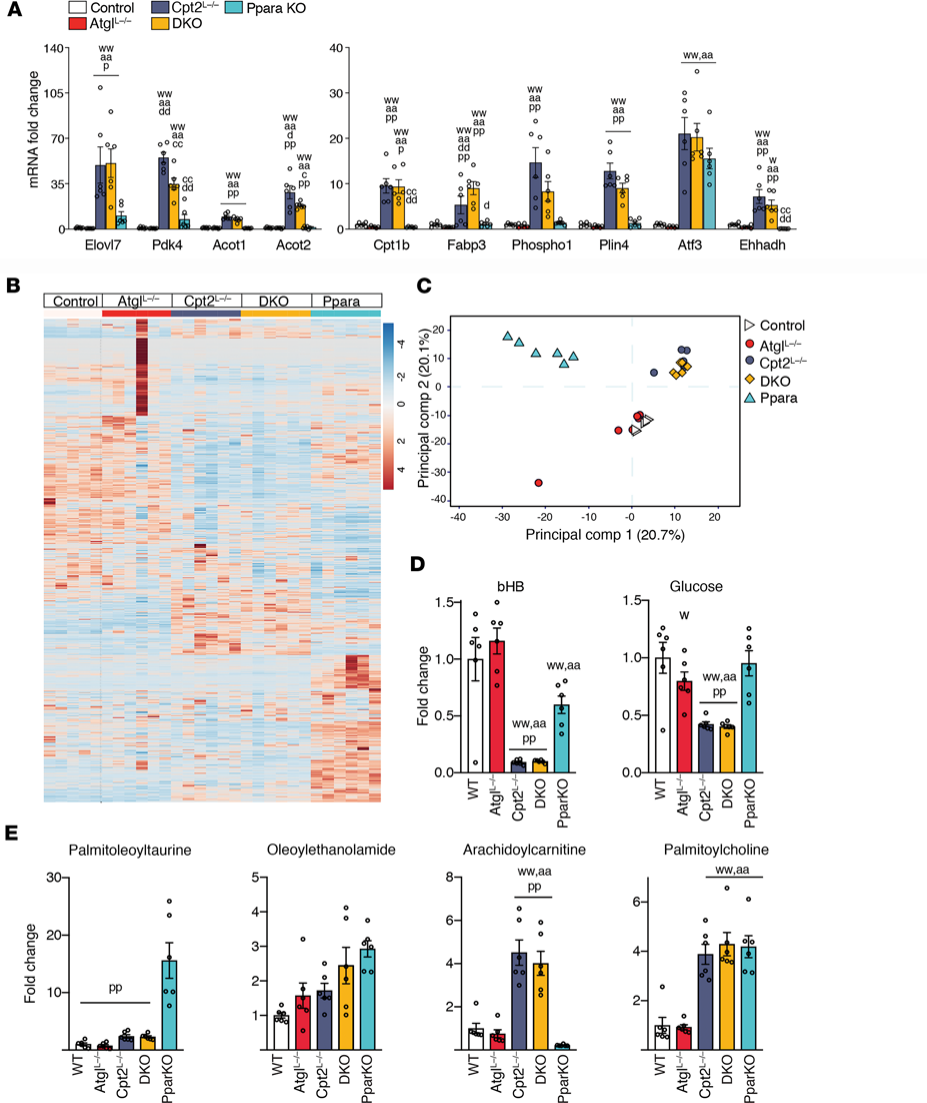
Specific defects in lipid catabolism generate unique molecular and metabolic phenotypes. (**A**) mRNA fold changes of selected Pparα target gene expression of 24-hour fasted liver. (**B**) Heatmap of liver metabolites extracted from 24-hour fasted male mice. (**C**) PCA of liver metabolome data. (**D**) Fold differences of 3-hydroxybutyrate and glucose in livers of 24-hour fasted male mice. (**E**) Fold differences of nonpolar metabolites extracted from the livers of 24-hour fasted male mice. One-way or 2-way ANOVA followed by Tukey’s multiple-comparison test were performed where appropriate to detect significance between genotypes (see also Supplemental Table 2). Single letter denotes *P* < 0.05. Double letters denote *P* < 0.01. Letters w (control), a (Atgl), c (Cpt2), d (DKO), and p (Pparα) represent significance between the genotypes. Data are shown as mean ± SEM.

Previously, we showed that the loss of hepatic fatty acid oxidation results in a robust induction of Pparα target genes in the liver in response to lipid availability (12, 13). Others have shown that inhibiting TG hydrolysis by the KO of Atgl results in a defect in Pparα-mediated transcription (3, 6–10). That is, the liberation of fatty acids from lipid droplets is important for Pparα-mediated transcription of fatty acid oxidative genes. Therefore, we examined the expression of a suite of Pparα target genes in control, Pparα-KO, Atgl^L–/–^, Cpt2^L–/–^, and DKO livers in response to a 24-hour fast. Although several genes such as *Fgf21* (Figure 2C), *Pdk4*, and *Acot2* showed a modest suppression in their induction upon the loss of Atgl in Cpt2^L–/–^ mice, most genes in DKO mice resembled the response of Cpt2^L–/–^ mice (Figure 4A). These data show that even though Atgl and Cpt2 are in the same pathway, they play independent roles in driving the transcriptional response to fasting.

To further understand the role of dysregulated lipid catabolism during fasting, freeze-clamped livers were collected from control, Pparα-KO, Atgl^L–/–^, Cpt2^L–/–^, and DKO mice following a 24-hour fast and subjected to untargeted metabolomics (Figure 4B and Supplemental Table 1; supplemental material available online with this article; https://doi.org/10.1172/jci.insight.135626DS1). Principal component analysis (PCA) showed the clustering of control and Atgl^L–/–^ metabolomes, and Cpt2^L–/–^ and DKO metabolomes, whereas Pparα-KO metabolome separated distinctly (Figure 4C). Similar to the transcriptional responses we observed, these steps in lipid catabolism result in unique metabolomic profiles. Glucose and β-hydroxybutyrate were suppressed in the Cpt2^L–/–^ and DKO livers owing to defects in gluconeogenesis and ketogenesis, respectively (Figure 4D). The different models accumulated lipids but different classes of lipids. For example, Pparα-KO mice exhibited a large induction of acyltaurines, whereas Cpt2^L–/–^ and DKO livers exhibited large increases in long-chain acylcarnitines, and all genotypes besides Atgl^L–/–^ livers exhibited increased palmitoylcholine (Figure 4E). These data show that individual defects in lipid catabolism result in unique molecular and metabolic phenotypes.

### Combined defects in TG hydrolysis and fatty acid oxidation lead to high-fat diet–induced NASH.

**Figure 5:**
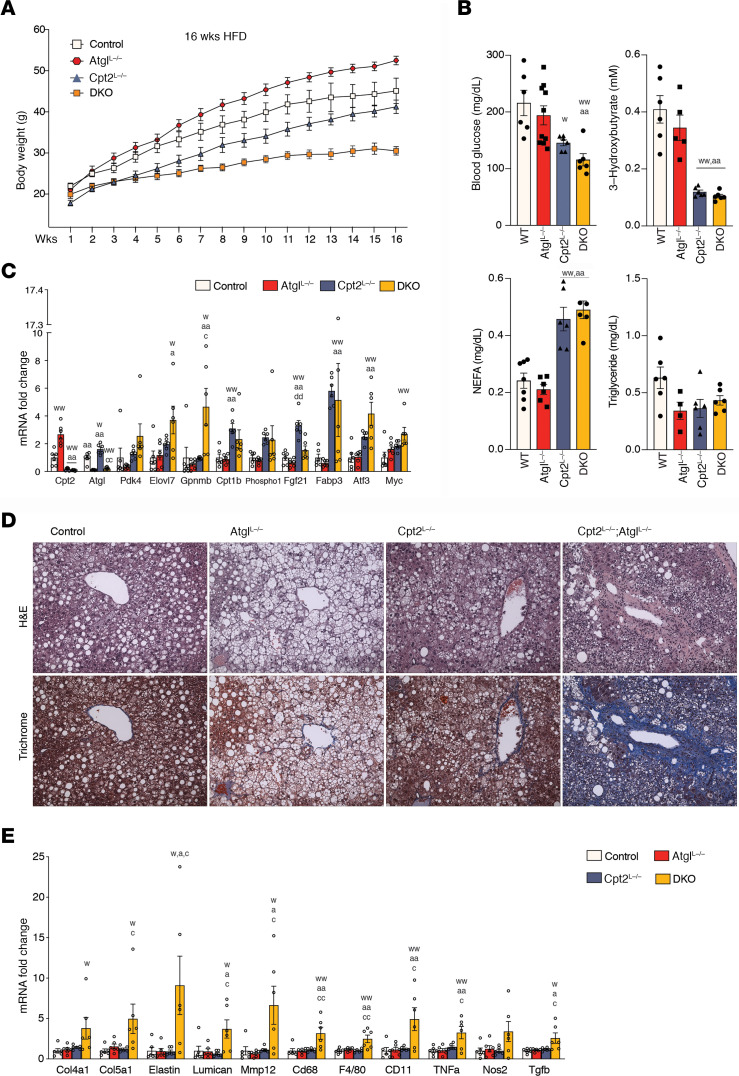
Combined defects in TG hydrolysis and fatty acid oxidation lead to HFD-induced NASH. (**A**) Body weights of control, Atgl^L–/–^, Cpt2^L–/–^, and DKO male mice fed an HFD for 16 weeks (*n* = 9–14). (**B**) Serum glucose, 3-hydroxybutyrate, NEFA, and TG concentrations of 16-week-old male HFD mice (*n* = 6). (**C**) HFD dramatically altered selected genes in liver of male mice. Gene expression represented as in fold changes relative to control (*n* = 6). (**D**) Histological morphology of the livers was assessed by H&E and trichrome staining. (**E**) HFD effect on inflammatory and fibrosis genes in livers of male mice. Gene expression represented as in fold changes relative to control (*n* = 6). One-way or 2-way ANOVA followed by Tukey’s multiple-comparison test were performed where appropriate to detect significance between genotypes. Single letter denotes *P* < 0.05. Double letters denote *P* < 0.01. Letters w (control), a (Atgl), c (Cpt2), d (DKO), and p (Pparα) represent significance between the genotypes. Data are shown as mean ± SEM.

Although fasting elicits a short-term delivery of fatty acids to the liver, high-fat feeding mediates a continuous delivery of lipid that necessitates further adaptations. To understand the role of TG hydrolysis and fatty acid oxidation in response to a high-fat diet (HFD), we placed control, Atgl^L–/–^, Cpt2^L–/–^, and DKO mice on an HFD for 16 weeks. As we have shown previously, Cpt2^L–/–^ mice are resistant to weight gain on an HFD (Figure 5A). Atgl^L–/–^ mice gained more weight on an HFD. Surprisingly, DKO mice gained almost no weight in response to an HFD, which may indicate a hepatotoxic effect (Figure 5A). Although control and Atgl^L–/–^ mice exhibited the expected diet-induced high blood glucose, Cpt2^L–/–^ and DKO mice exhibited glucose concentrations more similar to chow-fed mice owing to the inability of their livers to perform gluconeogenesis (13) (Figure 5B). As expected, Cpt2^L–/–^ and DKO mice did not produce appreciable ketone bodies and exhibited increased circulating NEFA (Figure 5B). The analysis of mRNA from Atgl^L–/–^, Cpt2^L–/–^, and DKO livers revealed a similar regulation to the HFD as to fasting (Figure 5C). However, several genes, including *Pdk4* and *Elovl7*, iwere now induced even higher in the DKO mice. Interestingly, the proto-oncogene *Myc* was also induced in DKO mice, which suggests a path toward the progression of hepatocellular carcinoma seen in late stages of NASH.

Given that DKO mice did not gain appreciable weight on the HFD, we suspected that the dual loss of Atgl and Cpt2 may lead to liver failure. Histological analysis of the livers revealed the development of steatosis in all of the livers with Atgl^L–/–^ livers exhibiting large lipid droplets (Figure 5D). DKO mice exhibited widespread areas of damaged liver with a great deal of mononuclear infiltration and significant fibrosis as revealed by accompanying Masson’s trichrome stain (Figure 5D and Supplemental Figure 1). Next, we profiled genes associated with fibrosis and inflammation in these mouse models. Consistent with the histological indications, control, Atgl^L–/–^, and Cpt2^L–/–^ livers revealed the same expression of genes such as *Col4a1*, *Mmp12*, and *Tnfa*, whereas the DKO livers showed significant elevation in both fibrosis and inflammatory genes (Figure 5E). These data show that the loss of TG hydrolysis or fatty acid oxidation alone is sufficient for the generation of steatosis but the loss of both is required for the generation of NASH.

**Figure 6:**
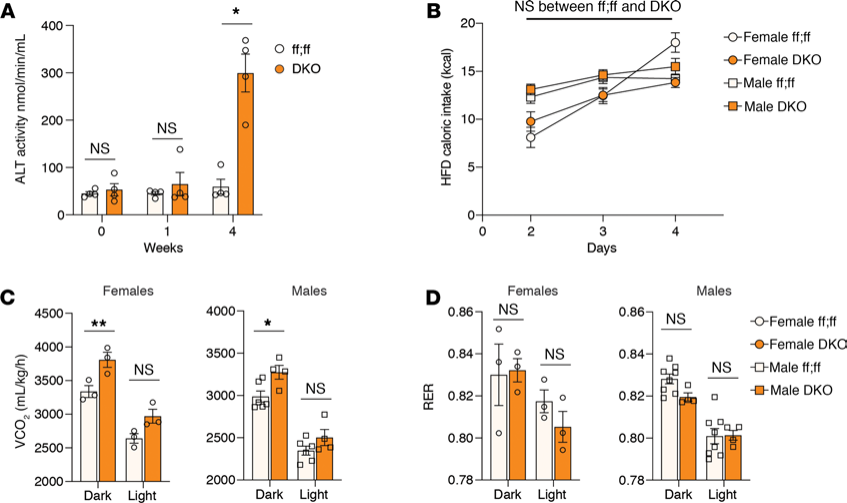
Four-week HFD is sufficient to induce liver damage while increasing energy expenditure in DKO animals. (**A**) Assessment of liver damage by serum ALT activity of 4-week-old male HFD mice (*n* = 4–6). (**B**) Quantification of caloric intake in males and female animals. (**C**) Representative volume of carbon dioxide (VCO_2_) measurement during Comprehensive Laboratory Animal Monitoring System (CLAMS) study. (**D**) Representative respiratory exchange ratio (RER) measurement during CLAMS study. Data are shown as mean ± SEM. Two-way ANOVA followed by Tukey’s multiple-comparison test were performed to detect significance between genotypes. *P < 0.05. Colors indicate genotypes. White, ff:ff (WT); orange, DKO. Shapes indicate sex. Circle, female; square, male.

To better understand the resistance to HFD-induced weight gain in DKO mice, we placed male and female DKO and littermate control mice on HFD for 4 weeks before differences in body weight were apparent. Given the additive resistance to HFD-induced weight gain over Cpt2^L–/–^ mice, we suspected that the observed liver damage (Figure 5D and Supplemental Figure 1) resulted in both inducing energy expenditure and suppressing food intake. Although 4 weeks of HFD was sufficient to induce liver damage in DKO mice (Figure 6A), we did not observe changes in food intake in male or female DKO mice (Figure 6B). However, we did observe increased energy expenditure (Figure 6C) without a change in respiratory exchange ratio (Figure 6D), similar to what we have observed for Cpt2^L–/–^ mice (13).

## Discussion

The progression from simple steatosis to NASH is likely caused by a combination of genetic and environmental factors. However, the first step is an excess storage of fatty acids as TG in hepatocytes. Fatty or steatotic livers can then progress to fibrotic and inflamed livers (NASH). These dense and fibrotic livers can further lead to liver failure and in some cases hepatocellular carcinoma. The role of hepatic fatty acid catabolism in the pathogenesis and progression of NASH, obesity, and diabetes in humans has been controversial owing to conflicting experimental results (18, 19). Although evidence exists for suppressed oxidative metabolism in humans with NASH (20, 21) strong evidence exists for an increase in oxidative metabolism (22, 23). However, it is unclear if this increased oxidative capacity is capable of meeting the demand for the apparent increase in lipid flux. Although mice have been excellent genetic models of steatosis, few models progress to NASH with significant inflammation and fibrosis. Here, we have shown that Atgl and Cpt2 are regulated in a compensatory manner, and the loss of both fatty acid oxidative capacity and TG hydrolysis results in progressive HFD-induced steatohepatitis.

TG hydrolysis and fatty acid β-oxidation exist in a continuous catabolic pathway. That is, fatty acids must first be liberated from lipid droplets before they can be made available for β-oxidation. Therefore, we were surprised at the striking differences in hepatocyte morphology, intermediary metabolism, and gene expression in mice with liver-specific defects in Atgl, Cpt2, and Pparα-KO mice. It had been previously postulated that lipids destined for fatty acid oxidation must be first funneled into the lipid droplet in a cell-autonomous manner. That is, exogenous fatty acids did not have direct access to mitochondria but instead first required entry into a lipid droplet. However, this idea of futile cycling during fatty acid oxidation has recently been challenged using adipose tissue as a model system, which demonstrated that exogenously derived fatty acids could fuel brown adipose tissue thermogenesis independent of cell-autonomous lipid droplet hydrolysis (24, 25). Similar to adipocytes, we have shown that the loss of Atgl in the liver does not lead to a loss in hepatocyte fatty acid oxidation as Atgl^L–/–^ mice generated ketone bodies with only modest deficits. Similarly, we have shown that Atgl^L–/–^ mice exhibited normal fasting-induced transcription, and the loss of Atgl in Cpt2^L–/–^ mice suppressed only a subset of highly induced Pparα transcriptional target genes. Others have shown that altering TG hydrolysis results in significant transcriptional changes in Pparα-responsive genes (3, 6–10). Global loss of Atgl or the loss of Atgl in cultured cells is critical for activating Pparα. However, our data suggest that in vivo adipocyte TG hydrolysis provides ample fatty acids to activate Pparα and stimulate mitochondrial β-oxidation independent of the hepatocyte lipid droplet. However, given the suppression of the highly Pparα-sensitive genes *Pdk4* and *Fgf21* in DKO mice, hepatocyte Atgl likely serves to provide fatty acids in an additive manner.

Atgl^L–/–^, Cpt2^L–/–^, and Pparα-KO mice livers all became lipid laden following a fast owing to their inability to efficiently catabolize lipids. However, the morphology stemming from their steatosis was unique. Although Atgl^L–/–^ mice exhibited large periportal lipid droplets, Cpt2^L–/–^ mice exhibited small uniformly distributed lipid droplets; Pparα-KO mice exhibited an intermediary lipid droplet phenotype. The microvesicular steatosis of Cpt2^L–/–^ livers resembles other defects, such as Reye’s syndrome and acute fatty liver of pregnancy (26, 27). Surprisingly, Atgl-Cpt2 DKO livers exhibited both large and small lipid droplet phenotypes. Given the unique lipid droplet phenotypes and incredible intracellular lipid burden, we decided to take a closer look with TEMs and observed abundant nuclear lipid droplets in Atgl^L–/–^ and DKO livers. Canonically, lipid droplets form at the ER in complex with the TG biosynthetic enzymes. However, lipid droplets have long been observed by TEMs in the nucleus, particularly in hepatocytes (28, 29). The purpose of nuclear lipid droplets and how they are generated remains enigmatic. However, our results suggest that Atgl may be required for nuclear hepatocyte TG hydrolysis.

Here, we have demonstrated the independent and interdependent roles of hepatic fatty acid oxidation and TG hydrolysis in response to starvation and consumption of an HFD. Atgl and Cpt2 are regulated in a compensatory manner, and the loss of these enzymes in the liver results in surprisingly unique hepatocyte morphology, intermediary metabolism, and gene expression in response to increased hepatic lipid flux. Moreover, the exaggerated Pparα-transcriptional response observed in fasted Cpt2^L–/–^ livers is not dependent on hepatic Atgl. Finally, the loss of TG hydrolysis or fatty acid oxidation alone is sufficient for the generation of steatosis; however, the loss of both Atgl and Cpt2 is required for the generation of significant steatohepatitis. Hepatic lipid catabolism is regulated at multiple steps, serving as feedback to regulate unique aspects of liver physiology.

## Methods

### Animals.

Cpt2^fl/fl^ and Cpt2^L–/–^ mice were previously described (12). Mice were housed in ventilated racks with a 14-hour light/10-hour dark cycle and fed a standard chow diet (2018SX, Teklad Global). To generate Atgl-Cpt2 DKO mice, we bred Atgl^fl/fl^ mice (The Jackson Laboratories 024278; ref. 30) to Cpt2^fl/fl^ mice. Albumin-Cre mice were obtained from The Jackson Laboratories (catalog 3574). Control mice were Atgl^fl/fl^ Cpt2^fl/fl^ littermates. For fasting experiments, mice were food deprived for 24 hours (3 pm to 3 pm). For fed studies, mice were food deprived for 4 hours (11 am to 3 pm). All mice were 8–9 weeks old at the time of sacrifice.

### Quantitative real-time PCR.

RNA was isolated from liver tissue using TRIzol reagent (Invitrogen, Thermo Fisher Scientific) and was further purified using RNeasy Mini Kit (QIAGEN), as recommended by the manufacturer. MultiScribe High-Capacity cDNA reverse transcription kit (Applied Biosystems, Thermo Fisher Scientific) was used to synthesize cDNA from 1 μg/μL RNA input. cDNA (2 ng/μL) was amplified with SsoAdvanced Universal SYBR Green (Bio-Rad) in the presence of selected primers (12). 18S and cycloA were used as housekeeping genes. Gene expression was normalized to 18S and cycloA averages. Data are expressed as 2-ΔCt.

### Western blot.

Frozen liver tissue pieces were homogenized in RIPA buffer (50 mM Tris-HCl at pH 7.4, 150 mM NaCl, 1 mM EDTA, 1% Triton X-100, and 0.25% deoxycholate) with PhosSTOP phosphoprotease inhibitor (Roche) and protease inhibitor cocktail (Roche). Homogenates were centrifuged at 4°C for 10 minutes at 12,000*g*. Supernatants were transferred to a new tube, and total protein concentrations were quantified by BCA assay (Thermo Fisher Scientific). A total of 30 μg lysate was separated by Tris-glycine SDS-PAGE (10% and 12% polyacrylamide), followed by a transfer to PVDF membranes (Immobilon). Membranes were blocked with 5% nonfat milk in TBS with Tween 20 (TBST) for 1 hour and incubated with primary antibodies at 1:1000 (total OXPHOS, ab110413, Abcam; Plin2, HPA016607, MilliporeSigma; Atgl, 2138, Cell Signaling Technology; Cpt2, PAS-122117, Pierce, Thermo Fisher Scientific) overnight. Heat shock chaperone 70 (7298, Santa Cruz Biotechnology) was used at 1:1000 as loading control. HRP-conjugated anti-rabbit (GE Healthcare, now Cytiva, NA934V) or fluorescence-based (Cy3-conjugated anti-mouse or Cy5-conjugated anti-rabbit; Invitrogen, Thermo Fisher Scientific) secondaries were used at 1:1000 where appropriate. Proteins were visualized using the Amersham Prime enhanced chemiluminescent substrate (Cytiva) or epifluorescence on an Alpha Innotech MultiImage III instrument.

### Histology.

For liver histology, tissue was fixed in 10% neutral buffered formalin, embedded in paraffin, sectioned, and stained with H&E or processed for trichrome staining (AML Laboratories Inc.). For TEMs, mice were perfused with a 2% paraformaldehyde, 2% gluteraldehyde-PBS solution, and a 3 mm^3^ block of liver was excised and further processed for TEMs with osmium tetroxide. Ultrathin sections were then cut and imaged with a Hitachi 7600 microscope as previously described (31).

### Serum and tissue metabolites.

Enzymatic and colorimetric assays were used to measure serum levels of 3-hydroxybutyrate (Stanbio B-HB LiquiColor Assay, EKF Diagnostics), NEFA (NEFA-HR; ref. 2; Wako Diagnostics), and TG (TR0100, MilliporeSigma). Tissue TG levels were measured as previously reported (32). Lipid peroxidation in liver tissue was measured with thiobarbituric acid reactive substances (TBARS) assay (Cayman Chemical) as directed by the manufacturer. Untargeted metabolomics from flash-frozen liver samples was performed by Metabolon Inc. Serum ALT activity was measured by ALT Activity Assay kit (MAK052, MilliporeSigma) as directed by the manufacturer. Hepatokine GDF15, FGF21, and Angplt3 serum levels were measured by Quantikine ELISA kits (R&D Systems, Inc).

### Gas chromatography/mass spectrometry.

Total fatty acids were analyzed as previously described (33), with a minor modification for tissue extraction. The Folch method was used to extract total lipids from frozen liver tissue pieces (approximately 50 mg). Bottom fraction was transferred to a new tube and dried under nitrogen. Lipids were further derivatized by adding 1.5 mL 1 N methanolic HCl and methanolized for 16 hours at 75°C. After methanolysis, the fatty acid methyl esters were extracted with 3 mL hexane, twice, then dried under nitrogen. Then 50 mm, 0.2 μL 0.11 μm film OV-1 and a 100 mm, 0.2 μm, 0.15 μm film SP-2560 capillary columns were used to separate fatty acid methyl esters. The columns were calibrated with the following standards that were injected at the beginning of each set of samples: NIH-F fame mix (plus added phytanic, pristanic, C27:0, and C26:0) and the Supelco 37 fame mix. The samples were first injected on the polar column, SP-2560. After the analysis, the sample vials were decapped, and hexane was added to the original 50 or 100 μL volume and recapped before analysis on the second nonpolar OV-1 column. The results were collected from the chemstation, the integration and calibration were checked, the calibrated results were then transferred to Microsoft Excel, and the results were compared and merged using Excel macros developed for the preparation of combined reports based on the identification and quantitation of the fatty acid methyl esters. Standards of known fatty acid composition and reagent blanks were analyzed with each set of samples.

### Statistics.

Data were analyzed using Prism software (GraphPad). Heatmap and PCA were generated by MetaboAnalyst (https://www.metaboanalyst.ca). Significance was determined using 1-way or 2-way ANOVA with Tukey’s post hoc correction for multiple-variable experiments. Supplemental Table 2 was generated using MetaboAnalyst statistical analysis software.

### Study approval.

All procedures were performed in accordance with the NIH *Guide for the Care and Use of Laboratory Animals* (National Academies Press, 2011) and under the approval of the Johns Hopkins University School of Medicine Animal Care and Use Committee.

## Author contributions

MJW conceptualized the project. ESS and MJW established the methodology, performed the visualizations, and wrote the original draft of the manuscript. MJW provided the resources. ESS, JC, and MJW provided formal analysis, conducted the investigations, and participated in the writing, review, and editing of the manuscript. MJW provided funding.

## Supplementary Material

Supplemental data

## Version 1 01/25/2021

Electronic publication
