# Supplementary material for: Discordant hepatic fatty acid oxidation and triglyceride hydrolysis leads to liver disease
Source: JCI Insight. 2021 Jan 25;6(2):e135626. doi: 10.1172/jci.insight.135626 (PMC7934875; doi:10.1172/jci.insight.135626)
Supplement: Supplemental data [file jciinsight-6-135626-s087.pdf]

Supp Figure 1. H&E and Trichrome staining of Liver Specific Double Knockout mice.

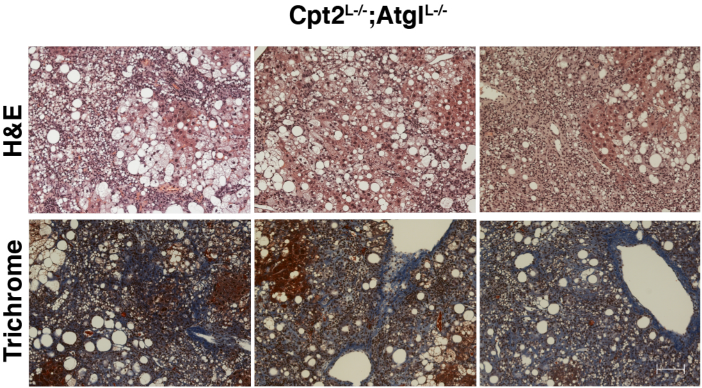

BIOCHEMICAL	SUPER PATHWAY	SUB PATHWAY	COMP ID	PLATFORM	CHEMICAL ID	PPAR-a	PPAR-a	PPAR-a	PPAR-a	PPAR-a	PPAR-a	Atgl cff	Atgl cff	Atgl cff	Atgl cff	Atgl cff	Atgl cff	ff:ff Cre	ff:ff Cre	ff:ff Cre	ff:ff Cre	ff:ff Cre	ff:ff Cre	Cpt2 cff	Cpt2 cff	Cpt2 cff	Cpt2 cff	Cpt2 cff	Cpt2 cff	ff:ff	ff:ff	ff:ff	ff:ff	ff:ff	ff:ff
(14 or 15)-methylpalmitate (a17:0 or i17:0)	Lipid	Fatty Acid, Branched	38768	LC/MS Neg	100002945	1.2237	1.4116	0.9531	1.1944	1.3607	1.1968	1.0967	1.3889	0.8840	1.8654	1.3897	0.8590	1.0459	0.9202	0.9652	1.1803	0.9719	0.9392	1.0428	1.0070	1.2972	0.9561	1.0598	1.2455	0.9102	0.8354	0.8549	0.8391	1.0218	0.9607
(16 or 17)-methylstearate (a19:0 or i19:0)	Lipid	Fatty Acid, Branched	38296	LC/MS Neg	100002356	1.0352	1.9525	1.1516	1.5282	1.9078	1.5025	1.1967	1.9097	0.8775	3.1641	1.4910	0.8601	0.8947	0.7813	0.9987	1.0329	0.9601	0.8940	0.7802	1.0222	1.2800	0.7590	0.9071	1.1202	0.8154	0.8244	1.0228	0.9389	1.1111	0.6921
(3'-5')-adenylyladenosine*	Nucleotide	Dinucleotide	62396	LC/MS Neg	100020723	1.4479	1.1685	0.6578	1.0000	0.9112	1.1944	1.7005	2.3850	1.0763	5.4651	1.0983	0.7482	0.3841	0.3200	0.4809	0.3200	0.3200	0.3200	0.3200	0.4958	0.3200	0.3200	0.3200	0.3200	1.0573	0.8672	1.3657	1.2390	1.0618	0.3200
(3'-5')-adenylylcytidine	Nucleotide	Dinucleotide	52753	LC/MS Neg	100009062	2.3939	0.6456	0.6409	1.3042	1.9977	1.6563	1.5467	2.0616	0.9297	15.5689	0.4007	0.4007	0.8191	0.4007	0.4007	0.5891	0.4007	0.4007	0.4007	0.4007	0.4007	0.4007	0.4007	0.4007	0.7552	0.4007	1.0000	1.2044	0.4007	0.5540
(3'-5')-adenylylguanosine*	Nucleotide	Dinucleotide	62760	LC/MS Neg	100020724	0.8363	0.6016	0.6016	0.6016	0.6016	0.6016	1.1134	1.3872	0.6016	3.5753	0.7120	0.6016	0.6016	0.6016	0.6016	0.6016	0.6016	0.6016	0.6016	0.6016	0.6016	0.6016	0.6016	0.6016	0.6016	0.6016	0.9213	0.7921	0.6016	0.6016
(3'-5')-adenylyluridine	Nucleotide	Dinucleotide	52740	LC/MS Neg	100009060	1.4491	1.1445	0.8027	0.9413	1.0793	1.3817	1.5335	2.2373	1.3435	19.6237	1.2626	0.6658	0.7170	0.4810	0.6234	0.6688	0.3779	0.2521	0.2381	0.5911	0.8223	0.2381	0.2500	0.2381	1.1308	0.7728	1.5398	1.5469	1.2332	0.5298
(3'-5')-cytidylylcytidine*	Nucleotide	Dinucleotide	62397	LC/MS Neg	100020727	0.9878	1.4227	0.8936	6.0017	3.8164	1.0122	0.9084	1.6997	0.4728	5.6757	1.2361	0.4728	0.6811	0.6707	0.8363	0.4728	0.5993	0.4728	0.4728	0.4802	0.4728	0.4728	0.4728	0.4728	0.4728	0.4728	0.7399	0.4728	0.9772	0.4728
(3'-5')-cytidylylguanosine	Nucleotide	Dinucleotide	52745	LC/MS Neg	100009063	0.3508	0.3508	0.3508	1.0000	0.3508	0.3508	0.3508	0.3508	0.3508	0.3508	0.3508	0.3508	0.3508	0.3508	0.3508	0.3508	0.3508	0.3508	0.3508	0.3508	0.3508	0.3508	0.3508	0.3508	0.3508	0.3508	0.3508	0.3508	0.3508	0.3508
(3'-5')-cytidylyluridine*	Nucleotide	Dinucleotide	62399	LC/MS Neg	100020728	0.5135	0.7896	0.5135	0.5135	0.5135	0.5135	0.5648	1.0000	0.5135	14.4022	1.0888	0.5135	0.5135	0.5135	0.5135	0.5135	0.5135	0.5135	0.5135	0.5135	0.5135	0.5135	0.5135	0.5135	0.5135	0.5135	1.1439	0.5135	0.5135	0.5135
(3'-5')-guanylylcytidine	Nucleotide	Dinucleotide	52746	LC/MS Neg	100009064	0.9691	1.0453	0.7225	2.9310	2.0243	0.8244	1.1262	1.7567	0.4932	23.0875	1.2938	0.4932	0.4932	0.4932	0.4932	0.4932	0.4932	0.5229	0.4932	0.7435	0.4932	0.4932	0.4932	0.4932	0.4932	0.4932	1.3662	0.4932	0.7549	0.4932
(3'-5')-guanylyluridine	Nucleotide	Dinucleotide	52743	LC/MS Neg	100009056	0.4822	0.4822	0.4822	0.4822	0.4822	0.4822	0.6775	1.0000	0.4822	31.1343	0.4822	0.4822	0.4822	0.4822	0.4822	0.4822	0.4822	0.4822	0.4822	0.4822	0.4822	0.4822	0.4822	0.4822	0.4822	0.4822	0.7178	0.4822	0.4822	0.4822
(3'-5')-uridylylcytidine*	Nucleotide	Dinucleotide	62398	LC/MS Neg	100020729	0.9059	1.1756	0.6981	1.0000	1.1273	0.6225	0.8995	1.4061	0.7229	3.3872	1.2285	0.5849	0.4375	0.6802	1.1720	1.4790	1.5866	1.0752	0.7555	0.6318	0.9814	0.8141	0.9839	1.1457	0.7331	0.8597	1.1694	1.0416	1.0826	0.4375
(3'-5')-uridylyluridine	Nucleotide	Dinucleotide	52741	LC/MS Neg	100009058	0.8261	1.2386	1.0914	1.1407	1.1711	1.0000	1.0619	1.4546	0.9276	5.6123	0.9785	0.8142	0.9176	0.3522	1.1101	0.8404	0.8403	0.9678	0.4607	0.7528	0.4641	0.6388	0.6630	0.4687	0.8558	1.0210	1.3573	1.3924	1.4628	0.3522
(R)-3-hydroxybutyrylcarnitine	Lipid	Fatty Acid Metabolism(Acyl Carnitine)	43264	LC/MS Pos Early	100003926	0.2030	0.3246	0.4069	0.4787	0.6162	0.1714	0.8718	1.2888	1.2868	0.7325	1.0000	2.0096	0.0283	0.0071	0.0194	0.0222	0.0240	0.0140	0.0150	0.0497	0.0071	0.0071	0.0248	0.0071	1.1206	1.4253	1.6051	1.4802	0.8468	0.0171
(S)-3-hydroxybutyrylcarnitine	Lipid	Fatty Acid Metabolism(Acyl Carnitine)	52984	LC/MS Pos Early	100009271	0.5627	0.6586	0.7871	1.9458	1.3749	0.5107	0.7784	1.1345	1.0458	0.8420	0.9906	1.7007	0.3467	0.3274	0.3699	0.3383	0.4001	0.3406	0.3227	0.4960	0.3041	0.2043	0.3748	0.4374	0.9114	1.2798	1.1633	1.3024	1.0122	0.3266
1-(1-enyl-oleoyl)-GPE (P-18:1)*	Lipid	Lysoplasmalogen	44621	LC/MS Neg	100005372	1.1175	1.6491	1.1126	0.9487	1.2835	2.7909	0.4539	0.7107	0.5204	10.0838	0.9731	0.3635	1.1338	1.5173	1.4268	1.5770	0.3635	0.8473	0.7967	0.6257	0.5901	1.2586	0.8863	0.8246	0.8565	0.7552	0.4795	0.4497	1.1550	1.2224
1-(1-enyl-palmitoyl)-2-arachidonoyl-GPE (P-16:0/20:4)*	Lipid	Plasmalogen	52673	LC/MS Pos Late	100009002	0.8556	1.0840	0.9698	0.8732	0.8516	0.9265	1.0095	1.0594	1.1043	2.2136	1.0842	1.0205	1.0212	0.8191	0.8393	0.9703	0.9021	0.8397	0.7535	0.8945	0.6063	0.7404	0.8012	0.7554	1.2114	0.9793	0.9642	1.1133	1.0469	0.8253
1-(1-enyl-palmitoyl)-2-linoleoyl-GPE (P-16:0/18:2)*	Lipid	Plasmalogen	52677	LC/MS Pos Late	100009069	0.4377	0.4163	0.4754	0.3963	0.3443	0.4898	1.1372	1.1434	1.4700	3.2330	1.0594	1.2140	0.7628	0.6139	0.5518	0.8583	0.7080	0.6535	0.7721	0.9724	0.4775	0.3150	0.7334	0.6695	1.2009	1.2468	1.0704	1.0035	1.3302	0.6951
1-(1-enyl-palmitoyl)-2-oleoyl-GPE (P-16:0/18:1)*	Lipid	Plasmalogen	52477	LC/MS Pos Late	100009005	0.8173	0.7188	0.7066	0.8001	0.6641	0.5914	1.1473	1.0633	1.1425	3.0168	0.8907	1.1817	1.0329	0.7023	0.8174	0.8542	1.0096	0.8254	0.5818	0.9904	0.7403	0.7365	0.8045	0.4926	1.1196	1.0248	0.9324	0.7425	1.0864	0.8309
1-(1-enyl-palmitoyl)-2-palmitoyl-GPC (P-16:0/16:0)*	Lipid	Plasmalogen	52716	LC/MS Pos Late	100009162	0.7998	0.8988	0.9053	0.9477	0.9404	1.1583	0.9812	1.0227	0.9674	1.5759	1.1782	0.9590	0.7049	0.5977	0.8079	1.0806	0.8732	0.8443	0.7415	0.8686	0.7777	0.5802	0.8716	0.8899	1.1106	1.0915	1.2721	1.1284	1.4552	0.7755
1-(1-enyl-palmitoyl)-GPE (P-16:0)*	Lipid	Lysoplasmalogen	39270	LC/MS Pos Late	100003000	0.8426	1.7006	1.4021	1.1596	0.8830	1.8264	0.6071	0.8109	0.7695	5.7515	1.0795	0.6658	1.1459	0.6725	0.9432	1.1822	0.9601	0.8783	0.9709	0.5970	1.6056	0.9829	0.8992	1.1031	1.0292	0.8091	1.0171	0.7235	0.6722	0.9501
1-(1-enyl-stearoyl)-2-arachidonoyl-GPE (P-18:0/20:4)*	Lipid	Plasmalogen	52475	LC/MS Pos Late	100008999	0.8289	1.0720	0.9123	0.8710	0.7904	0.9476	1.0091	1.0101	1.1102	1.5938	0.9909	1.0565	0.8578	0.7301	0.7734	0.9029	0.8690	0.8857	0.7500	0.8244	0.5246	0.7131	0.7872	0.7276	1.0849	0.9717	0.9101	1.0134	1.0826	0.8014
1-(1-enyl-stearoyl)-2-linoleoyl-GPE (P-18:0/18:2)*	Lipid	Plasmalogen	52748	LC/MS Pos Late	100009225	0.7867	0.7616	0.6795	0.8149	0.7190	0.8180	1.0667	0.8391	1.1445	2.0347	1.1126	1.2371	0.6883	0.6240	0.7112	0.9684	1.0052	0.6527	0.8025	1.1024	0.6097	0.5657	0.8082	0.6368	0.9553	1.0320	1.1262	0.9086	1.4317	0.7826
1-(1-enyl-stearoyl)-GPE (P-18:0)*	Lipid	Lysoplasmalogen	39271	LC/MS Pos Late	100003001	1.0335	2.0080	1.6187	1.2822	1.4868	2.4247	0.6692	0.9327	0.7132	4.2824	1.1229	0.6743	0.8193	0.6201	0.8111	1.3500	0.9341	0.8981	1.0880	0.5583	1.5290	0.9041	0.9881	0.8900	0.8921	0.8391	0.8032	0.6103	0.8119	1.1303
1,2-dilinoleoyl-GPC (18:2/18:2)	Lipid	Phosphatidylcholine (PC)	52603	LC/MS Pos Late	100008903	1.7137	2.1110	1.7718	1.5241	1.5846	2.0127	0.8323	1.0118	0.8701	1.1993	0.9669	0.9216	0.9780	0.8166	0.7937	1.0381	1.0434	0.9788	1.2248	1.3539	1.3963	1.2616	1.2283	1.1519	0.8092	0.9225	1.0171	0.9768	0.9869	0.8331
1,2-dilinoleoyl-GPE (18:2/18:2)*	Lipid	Phosphatidylethanolamine (PE)	53174	LC/MS Pos Late	100009217	3.0256	3.4121	3.6226	3.7404	3.8016	4.1837	0.7811	0.7396	1.0453	0.8259	0.7780	0.9547	1.3939	1.1275	1.6507	1.7181	1.4414	2.4221	3.1022	2.7230	3.1749	2.5368	2.2513	[illegible]	0.6696	0.8881	0.9080	0.8770	1.0683	1.2923
1,2-dipalmitoyl-GPC (16:0/16:0)	Lipid	Phosphatidylcholine (PC)	19130	LC/MS Pos Late	100000657	0.8844	1.0263	0.9721	0.8441	0.7522	0.8522	1.1141	1.1197	1.0178	1.2087	1.0486	0.9861	0.9027	0.7529	0.6994	0.7593	0.7349	0.6212	0.8063	0.8325	0.7593	0.7564	0.7143	0.6140	1.1941	1.0079	1.1030	1.0424	1.0893	0.6729
1,2-dipalmitoyl-GPE (16:0/16:0)*	Lipid	Phosphatidylethanolamine (PE)	57341	LC/MS Pos Late	100009204	0.9813	0.9389	0.9184	0.9314	0.5445	0.7393	1.4520	0.9613	1.2714	1.3178	0.8921	0.9723	1.1105	0.8250	0.8875	0.7341	0.4983	0.4045	1.0646	1.3080	0.8589	0.7499	0.6195	0.3329	1.1978	1.2965	0.9334	0.8658	1.0661	0.6181
1,5-anhydroglucitol (1,5-AG)	Carbohydrate	colysis, Gluconeogenesis, and Pyruvate Metabo	20675	LC/MS Neg	100000580	1.5667	1.3322	1.1394	0.9611	1.0322	1.1042	0.9297	1.0793	0.7370	1.1235	0.8611	0.9119	1.1732	0.6196	0.9569	1.0169	0.9366	0.9519	0.7307	1.1856	1.1648	0.9207	0.8751	0.9419	1.1465	1.0983	0.7538	0.9674	0.7553	0.8950
10-heptadecenoate (17:1n7)	Lipid	Long Chain Fatty Acid	33971	LC/MS Neg	100001278	0.9850	1.1425	0.8483	0.8268	1.0917	1.0341	1.2912	1.8886	0.7270	3.0846	1.2493	0.7419	0.7070	1.0056	1.3571	1.3312	1.0652	1.1163	1.2015	1.0861	1.2044	1.1596	1.1565	1.7996	0.5849	0.5651	0.8096	0.6906	0.7233	0.9944
10-nonadecenoate (19:1n9)	Lipid	Long Chain Fatty Acid	33972	LC/MS Neg	100001277	1.3054	2.3284	1.2485	1.3600	1.6167	1.6700	1.5448	2.5366	0.7886	5.5328	1.6220	0.7446	0.7624	0.7062	0.8971	1.0030	0.8337	0.8790	0.8661	0.8378	1.7736	0.9284	0.8916	1.4345	0.8041	0.7345	1.1012	0.8946	1.0236	0.7592
12,13-DiHOME	Lipid	Fatty Acid, Dihydroxy	38395	LC/MS Neg	62	3.4280	3.8629	2.7559	2.8261	2.1091	3.0766	0.9483	1.2177	0.7128	4.5166	1.0850	0.6558	0.8988	0.7704	0.9868	1.0132	1.1521	1.2238	0.9617	0.8118	1.4787	0.9859	1.0195	2.0946	0.7391	0.7921	1.5701	1.0284	1.1311	0.8136
12-dehydrocholate	Lipid	Secondary Bile Acid Metabolism	44644	LC/MS Neg	100001072	0.0842	0.9202	0.7295	0.3249	0.1359	1.7083	3.4558	1.5209	1.7722	0.4749	0.5894	1.5240	0.6805	0.0860	0.5366	2.9847	0.3963	2.0768	2.6105	0.1742	0.9603	0.7530	3.6950	0.9439	2.4176	2.0299	1.9366	1.5212	1.3790	0.4325
12-HEPE	Lipid	Eicosanoid	46293	LC/MS Neg	100002165	1.2632	0.8329	0.7090	0.4856	0.3443	0.5520	1.5009	0.9473	0.9487	0.7874	0.8460	1.1928	1.1291	0.9289	0.8469	0.8043	0.9307	0.6147	1.4397	1.2076	0.9093	0.9990	0.9798	[illegible]	1.2630	0.9687	1.1960	1.1395	1.2447	0.8796
12-HETE	Lipid	Eicosanoid	37536	LC/MS Neg	100002167	1.0432	0.8695	0.7693	0.5503	0.3253	0.5775	1.7949	1.0295	0.9834	0.4314	1.0071	1.2821	0.9929	0.7185	0.6937	0.6282	0.6165	0.5146	0.9028	1.2389	1.2290	0.5819	0.8481	0.5795	1.8319	1.6654	0.9503	1.2393	1.3708	0.8790
12-HHTrE	Lipid	Eicosanoid	57393	LC/MS Neg	1261	1.0173	0.9319	0.7048	1.6995	0.5595	0.5964	1.8740	1.6770	2.0053	0.5055	1.0042	0.9958	2.3129	0.6271	0.8153	0.7535	0.7867	0.6914	0.5878	0.8940	0.6767	0.4816	0.5674	0.6084	1.5250	1.6579	0.8987	1.5054	1.9947	0.5820
13-HODE + 9-HODE	Lipid	Fatty Acid, Monohydroxy	37752	LC/MS Neg	100002196	1.6553	1.4319	1.1585	1.8410	1.1427	0.8528	1.1593	1.4058	0.8937	0.7553	1.0233	0.6840	1.0059	0.7566	1.1177	1.0597	0.8344	1.3487	0.9955	0.8090	2.2868	1.3491	1.3820	2.8447	0.7845	0.7725	1.2792	1.0589	0.9983	0.7865
14,15-DiHETrE	Lipid	Fatty Acid, Dihydroxy	46305	LC/MS Neg	100002752	1.6979	1.8141	1.7845	2.2354	1.4590	1.8313	1.2734	1.1718	0.8472	2.0774	1.2461	0.9766	0.8907	0.5653	0.8152	1.2364	0.9075	0.8400	0.6383	1.4133	0.6324	0.3274	0.6864	0.8070	0.9794	1.2360	1.1011	1.0046	1.2671	0.7897
14-HDoHE/17-HDoHE	Lipid	Docosanoid	53034	LC/MS Neg	100009278	1.0956	1.1674	0.6730	0.5032	0.4849	0.7137	1.2452	0.7929	0.7478	0.6686	0.9599	0.9218	0.8567	0.7480	0.7852	0.7691	0.9324	0.4776	1.0675	0.9656	1.4192	0.8413	0.9668	0.5721	1.8510	1.3748	1.1887	1.3949	1.7066	0.8014
16-hydroxypalmitate	Lipid	Fatty Acid, Monohydroxy	39609	LC/MS Neg	100002953	0.7822	0.9836	1.2121	1.4872	0.9770	0.8959	0.8290	1.0851	0.6523	1.5881	1.1935	0.7283	0.9786	0.8765	1.2130	1.1472	1.4910	0.8477	1.3589	0.9842	0.4631	1.1925	1.3246	[illegible]	1.0158	0.8114	1.3340	1.1368	1.2149	1.5597
19,20-DiHDPA	Lipid	Fatty Acid, Dihydroxy	46286	LC/MS Neg	100002844	2.1527	2.5014	2.6018	2.9692	2.1767	2.3414	1.0026	1.2881	0.8048	3.0930	1.1963	0.8304	0.7742	0.7282	0.8087	1.1640	1.0245	0.8705	0.6933	0.9423	1.3697	0.6055	0.7133	0.8893	0.8542	1.0507	1.3496	0.9731	1.4465	0.8038
1-arachidonoyl-GPC* (20:4)*	Lipid	Lysophospholipid	34061	LC/MS Neg	100001551	0.7798	0.8485	0.6913	0.5584	0.6248	0.9653	1.4572	1.8783	0.8592	1.8199	1.3110	1.0783	1.2065	0.9241	1.2007	1.3063	0.7937	0.8824	0.8308	1.0489	0.6489	0.4392	0.7471	0.7713	1.5910	1.3815	1.0105	1.4376	2.0623	0.9895
1-arachidonoyl-GPE (20:4n6)*	Lipid	Lysophospholipid	35186	LC/MS Pos Late	100001571	0.8076	1.0858	0.7305	0.5672	0.5479	0.8542	1.1825	1.3445	0.7613	2.4343	1.1632	0.7505	1.1493	0.8049	1.1653	1.0125	0.8238	0.9875	0.9161	1.2531	0.6850	0.6658	0.9558	0.8068	1.1668	1.2172	1.0509	1.1947	1.2194	0.8219
1-arachidonoyl-GPI* (20:4)*	Lipid	Lysophospholipid	34214	LC/MS Neg	100001654	0.7125	0.9941	0.7076	0.3662	0.6155	0.9497	1.3936	1.3891	0.8083	0.9298	1.3396	1.0280	1.2402	0.9761	1.4493	1.3531	0.8377	1.1268	1.0171	1.0059	0.9167	0.6496	1.0173	0.9165	1.3180	0.9794	1.1859	1.5231	1.7081	1.2624
1-arachidonylglycerol (20:4)	Lipid	Monoacylglycerol	52401	LC/MS Pos Late	100001433	0.8120	1.3328	0.4858	0.8006	0.8894	0.8244	1.9754	4.1633	0.8201	77.1537	0.9859	0.9696	0.4858	0.4858	0.4858	0.4858	0.4858	0.5212	0.4858	0.7954	1.1350	0.4858	0.7350	0.4858	1.4238	1.2201	1.4756	1.2539	0.7732	0.4858
1-carboxyethylisoleucine	Amino Acid	Leucine, Isoleucine and Valine Metabolism	62558	LC/MS Neg	100020427	0.3748	1.0095	0.6406	0.3748	0.5584	0.6721	2.2105	1.0687	1.3107	1.9697	0.6206	1.1425	0.9300	0.3748	0.3963	0.9261	0.7903	1.0611	0.6588	0.8811	1.1305	0.5815	1.2488	0.5108	0.4997	0.6175	1.9984	0.9905	0.8179	0.6470
1-carboxyethylleucine	Amino Acid	Leucine, Isoleucine and Valine Metabolism	62559	LC/MS Neg	100020425	0.5150	0.8193	0.7961	0.8049	0.7142	0.5708	1.7072	0.9613	1.0431	0.4093	0.9312	1.4716	0.7734	0.4598	0.4692	0.9493	1.0727	0.8225	0.7477	0.9777	1.0611	0.7522	1.3947	1.0994	0.8806	0.8197	1.9316	1.0798	1.0223	0.6440
1-carboxyethylphenylalanine	Amino Acid	Phenylalanine Metabolism	62566	LC/MS Neg	100020419	0.5056	1.1386	0.6760	0.3910	0.4359	0.7735	1.8559	0.6480	1.0981	0.4481	0.8278	1.4995	0.9153	0.4544	0.6171	1.3235	0.9453	0.9608	0.7020	0.8362	1.2260	1.0694	1.5805	0.9805	0.8630	0.6593	1.8650	1.0286	0.9798	0.4572
1-carboxyethylvaline	Amino Acid	Leucine, Isoleucine and Valine Metabolism	62562	LC/MS Neg	100020423	0.5005	0.9183	0.6573	0.5485	0.6067	0.7508	1.5728	0.8793	1.1638	0.7275	0.8361	1.4473	0.9945	0.4723	0.5376	1.0055	0.9376	0.9685	0.7121	0.8814	0.8699	1.0514	1.2682	1.0264	1.1414	0.7170	1.8523	1.0697	0.7817	0.7300
1-dihomo-linolenylglycerol (20:3)	Lipid	Monoacylglycerol	48341	LC/MS Neg	100006121	0.4777	1.0000	0.6656	0.2970	0.2970	0.8990	1.9539	3.2853	0.2970	89.7223	0.6290	0.2970	0.2970	0.2970	0.2970	0.2970	0.2970	0.2970	0.2970	0.2970	0.8298	0.2970	0.2970	0.2970	0.5103	1.0304	0.9970	0.8864	1.1480	0.2970
1-dihomo-linoleoylglycerol (20:2)	Lipid	Monoacylglycerol	35103	LC/MS Neg	100001483	0.4820	1.5876	1.1274	1.3486	1.1550	1.0019	1.9238	3.5021	0.3259	90.3371	1.0174	0.3259	0.3259	0.3259	0.3259	0.3259	0.3259	0.3259	0.3259	0.3259	0.7033	0.3259	0.3259	0.3259	0.9619	0.6199	1.4618	0.7529	0.9981	0.3259
1-docosahexaenoylglycerol (22:6)	Lipid	Monoacylglycerol	35153	LC/MS Pos Late	100001481	1.0064	2.1000	1.0217	0.9936	0.8734	1.2254	1.7531	5.3307	0.8371	520.7250	1.6236	0.6174	0.4410	0.4308	0.4100	0.7781	0.5636	0.6528	0.5748	1.1129	1.0528	0.6379	0.5988	0.5285	1.0516	0.8923	2.6429	2.1173	0.8389	0.3130
1-heptadecenoylglycerol (17:1)*	Lipid	Monoacylglycerol	62449	LC/MS Neg	100020769	0.1427	0.1427	0.1427	0.1427	0.1427	0.1427	1.0244	0.1427	0.1427	8.4228	0.1427	0.1427	0.1427	0.1427	0.1427	0.1427	0.1427	0.1427	0.1427	0.1427	0.5536	0.1427	0.1427	0.1427	0.1427	0.1427	0.1427	0.1427	0.1427	0.1427
1-lignoceroyl-GPC (24:0)	Lipid	Lysophospholipid	49617	LC/MS Pos Late	100002873	1.0713	0.9107	1.0096	0.7692	0.9644	1.1786	1.0455	0.9369	0.8080	1.3733	0.7630	0.9904	0.5279	0.5277	0.6902	0.7023	0.5971	0.6885	0.7086	0.6877	0.5277	0.5277	0.7261	0.5781	1.0735	1.0848	1.1034	0.8630	1.3154	0.7226
1-linolenoylglycerol (18:3)	Lipid	Monoacylglycerol	34393	LC/MS Neg	100001435	1.6604	3.0915	1.5478	1.4265	1.3378	2.8740	2.6469	6.6414	0.5725	103.7453	1.9185	0.5711	0.9290	0.5495	0.5948	0.7320	0.5480	0.8198	2.0701	1.4467	5.0124	0.7642	0.7720	0.6159	0.6767	0.7430	1.7930	0.9277	1.2400	0.3798
1-linoleoyl-GPC (18:3)*	Lipid	Lysophospholipid	45951	LC/MS Pos Late	100000350	1.7979	2.2043	1.8830	1.2313	1.2173	2.0210	1.0406	1.2759	0.8514	1.5551	0.9812	0.8906	0.4637	0.4375	0.5399	0.6122	0.4278	0.4308	0.5282	0.8898	1.0724	0.8477	0.5438	0.5206	1.0835	1.0188	1.1649	1.1596	1.2679	0.3480
1-linoleoyl-2-arachidonoyl-GPC (18:2/20:4n6)*	Lipid	Phosphatidylcholine (PC)	52710	LC/MS Pos Late	100009131	0.8941	0.9893	0.9004	0.6572	0.7274	1.1127	1.3860	1.4712	1.1060	1.2174	1.0396	1.1977	0.6747	0.5225	0.5435	0.8925	0.7508	0.7422	0.7817	1.1675	0.5833	0.6040	0.7563	0.6352	1.2193	1.1899	1.1662	1.2893	1.5039	0.5930
1-linoleoyl-2-arachidonoyl-GPE (18:2/20:4)*	Lipid	Phosphatidylethanolamine (PE)	53189	LC/MS Pos Late	100009222	1.1980	1.2339	1.3286	1.3043	1.3636	1.5564	1.3004	1.0885	1.1494	1.0403	0.8194	1.1036	0.8726	0.6721	0.7881	0.9777	0.8615	0.7766	1.0754	1.4457	0.9516	0.9395	1.0271	0.8268	0.9827	0.9832	0.8746	0.9280	1.2840	0.8272
1-linoleoyl-2-linolenoyl-GPC (18:2/18:3)*	Lipid	Phosphatidylcholine (PC)	53176	LC/MS Pos Late	100009343	1.9210	2.0468	2.5539	1.9199	1.9652	2.4084	1.1347	1.3486	1.2071	0.8839	0.8950	1.5828	0.5285	0.3832	0.4327	0.7096	0.3095	0.5904	0.9170	1.5298	0.9036	0.9449	0.8002	0.7041	0.9837	1.0645	1.3249	1.3063	1.3012	0.5174
1-linoleoylglycerol (18:2)	Lipid	Monoacylglycerol	27447	LC/MS Neg	100001040	0.1616	1.8627	0.5247	1.0000	0.7591	1.0242	5.6979	23.2731	0.6844	1118.3171	2.3855	0.1616	0.3086	0.1616	0.1616	0.3577	0.2142	0.1616	0.3019	0.9985	2.9324	0.5223	0.3494	0.3252	0.4799	0.1616	5.3472	1.2910	1.1728	0.1616
1-linoleoyl-GPC (18:2)	Lipid	Lysophospholipid	34419	LC/MS Pos Late	100001395	1.6592	2.4559	1.8863	1.0351	0.9792	2.0871	0.9840	1.3743	0.6683	2.4234	1.0577	0.7416	1.0549	0.7833	0.9240	1.1246	0.9371	0.9545	1.0160	1.2247	1.0610	0.6459	1.0747	1.0474	0.9752	0.8676	1.0894	0.9577	1.0503	0.8000
1-linoleoyl-GPE (18:2)*	Lipid	Lysophospholipid	36600	LC/MS Pos Late	100001570	1.7712	2.4858	1.9246	1.6039	1.5118	2.3392	0.7942	0.8868	0.6714	1.8609	0.9371	0.7329	1.3630	0.9556	1.4065	1.2482	1.0833	1.2093	1.5288	1.7307	1.8568	1.3777	1.7795	1.5843	1.0188	0.9668	0.8355	0.8793	0.9812	1.0723
1-linoleoyl-GPG (18:2)*	Lipid	Lysophospholipid	54885	LC/MS Neg	100009227	1.3744	1.2194	1.0268	0.6960	0.7752	1.5240	0.7866	0.9831	0.5061	0.3711	1.1411	0.5366	1.6246	0.7167	1.5046	1.2801	0.8170	0.5878	0.8888	0.6574	0.4066	0.5796	0.6164	0.3711	1.1034	1.0478	0.7547	1.0993	1.1645	1.1211
1-linoleoyl-GPI* (18:2)*	Lipid	Lysophospholipid	36594	LC/MS Neg	100001778	2.3308	1.8924	1.8274	1.4657	1.4176	2.0136	1.2639	1.1984	0.8823	14.7998	1.5917	0.6437	1.4831	0.6108	1.1657	0.7547	0.6351	0.7004	1.0599	0.9400	0.7384	0.4802	0.9401	0.6899	1.1541	0.8057	1.1780	1.4817	1.3269	0.6517
1-linoleoyl-GPS (18:2)*	Lipid	Lysophospholipid	43676	LC/MS Neg	100004606	2.8360	2.5725	2.0485	1.9933	2.5312	4.1388	0.5112	0.4280	0.4473	0.4280	0.4280	0.4280	1.1532	1.0102	1.0845	0.4280	0.4280	0.4280	1.0167	0.4399	1.2019	0.9898	0.8085	0.4280	1.2034	0.8938	0.5118	0.4280	1.1581	0.6580
1-margaroylglycerol (17:0)	Lipid	Monoacylglycerol	34391	LC/MS Neg	100001429	0.2514	0.3284	0.2514	1.0000	0.6647	1.3454	0.2514	0.6136	0.2514	2.2564	0.2514	0.2514	0.2514	0.2514	0.2514	0.2514	0.2514	0.2514	0.2514	0.2514	0.2514	0.2514	0.2514	0.2514	0.2514	0.2514	0.2514	0.2514	0.2514	0.2514
1-methyl-4-imidazoleacetate	Amino Acid	Histidine Metabolism	32350	LC/MS Pos Early	100001208	2.0397	2.7117	4.7281	6.0464	6.1272	4.9162	0.9265	1.0834	0.7035	0.7052	0.8502	1.1489	1.0056	0.7836	0.8365	1.1444	2.2330	1.0355	0.8493	1.1257	1.9175	1.4988	1.0680	1.2136	1.3652	0.9380	0.7268	0.7372	0.9087	1.1401
1-methyl-5-imidazoleacetate	Amino Acid	Histidine Metabolism	62946	LC/MS Pos Early	100002910	1.1965	0.8864	1.1259	0.9105	1.1928	1.0029	2.5764	2.7681	1.2230	1.3632	0.3457	0.7307	1.4083	0.7873	0.7235	0.9207	0.9787	0.7213	0.1118	0.5437	0.6817	0.3457	1.0574	0.7641	9.7881	0.8534	1.3420	1.3075	1.6023	0.5727
1-methylhistamine	Amino Acid	Histidine Metabolism	43831	LC/MS Pos Early	100001204	1.1752	1.1833	1.2874	1.2923	0.7870	0.9051	0.6499	1.2574	0.8627	0.9114	0.6721	0.4381	0.4994	0.4301	0.7254	0.9994	0.6915	0.4880	0.6919	0.6692	0.5486	0.5580	1.0006	0.7217	2.6004	1.3805	0.5689	1.1780	2.4666	0.6525
1-methylhistidine	Amino Acid	Histidine Metabolism	30460	LC/MS Pos Early	100001051	0.9665	1.5269	1.0175	0.7515	0.8051	1.4440	1.1329	0.9100	0.8371	0.6483	0.8406	0.7141	1.8245	1.3276	1.2202	0.9825	1.7043	1.3201	1.5607	1.3906	0.5566	0.2153	1.3093	1.3456	1.2414	0.9388	0.9186	1.3131	1.0899	1.3255
1-methylnicotinamide	factors and Vitami	Nicotinate and Nicotinamide Metabolism	27665	LC/MS Pos Early	55	1.3577	1.0244	2.0385	1.5487	2.2940	1.9144	0.4307	0.6399	0.4506	1.4439	0.6848	0.7844	0.5527	0.4778	0.7749	0.8205	0.5476	0.6872	0.5178	0.2803	0.1167	1.3164	0.3456	0.1155	0.7116	0.8611	1.1001	0.9845	1.3297	0.6319
1-myristoyl-2-arachidonoyl-GPC (14:0/20:4)*	Lipid	Phosphatidylcholine (PC)	53195	LC/MS Pos Late	100009139	1.4869	1.4573	1.1812	1.3561	1.2516	1.5588	0.8142	1.0514	0.7478	0.8808	1.1862	0.4880	1.1719	0.9757	0.9611	1.3380	1.1354	1.4535	1.1378	0.9890	0.6969	0.6750	1.1057	1.0110	0.9400	0.8015	0.9554	0.9419	0.9245	1.0966
1-myristoyl-2-palmitoyl-GPC (14:0/16:0)	Lipid	Phosphatidylcholine (PC)	19258	LC/MS Pos Late	100000672	1.5799	1.2772	1.0996	1.2482	1.2891	1.5133	0.9510	0.8411	0.8977	1.2645	0.8542	0.6063	1.1737	0.7895	1.0900	1.2592	1.0278	0.8653	1.0444	1.0642	0.9977	0.7397	0.7915	0.7482	0.8707	0.9343	0.9613	0.8002	0.9797	1.0023
1-oleoyl-2-arachidonoyl-GPE (18:1/20:4)*	Lipid	Phosphatidylethanolamine (PE)	55041	LC/MS Pos Late	100009219	0.6204	0.6567	0.5995	0.5611	0.5511	0.5672	1.0968	1.2761	1.0377	0.8370	1.1462	1.0221	0.8041	0.8275	0.8135	0.8552	0.9961	0.9998	0.8621	0.8273	0.7014	0.7216	1.0395	1.0002	1.2538	0.9057	0.9624	1.1820	1.0888	0.8162
1-oleoyl-2-arachidonoyl-GPI (18:1/20:4)*	Lipid	Phosphatidylinositol (PI)	54994	LC/MS Polar	100010951	0.6505	0.8075	0.7326	1.3164	1.1634	0.7550	1.2701	1.1851	1.0289	1.2050	0.9540	1.0340	0.5663	0.5987	0.7482	0.8128	0.7334	0.5582	0.7715	1.4212	0.8481	0.8176	0.9047	0.7845	1.0943	1.0801	1.1512	1.3032	0.9711	0.6858
1-oleoyl-2-docosahexaenoyl-GPC (18:1/22:6)*	Lipid	Phosphatidylcholine (PC)	52697	LC/MS Pos Late	100009130	0.5970	0.6495	0.4743	0.2765	0.2993	0.4790	1.2665	1.5120	0.9890	0.8761	1.2141	1.2041	0.7146	0.6794	0.6958	0.8196	0.8981	0.8631	0.8366	1.0214	0.6419	0.7532	0.9830	0.9211	1.3166	0.9328	1.1687	1.3268	1.1816	0.7095
1-oleoyl-2-docosahexaenoyl-GPE (18:1/22:6)*	Lipid	Phosphatidylethanolamine (PE)	53209	LC/MS Pos Late	100009220	0.7425	0.8131	0.6766	0.5037	0.5031	0.6232	1.0779	1.1254	1.0723	0.8185	1.0583	0.9458	0.8075	0.7825	0.7465	0.8694	0.9959	1.0081	0.9005	0.9866	0.6637	0.7358	0.9949	0.9858	1.1773	0.8973	1.3025	1.1916	1.2229	0.7548
1-oleoyl-2-linoleoyl-GPE (18:1/18:2)*	Lipid	Phosphatidylethanolamine (PE)	52687	LC/MS Pos Late	100009078	1.4857	1.7954	1.5426	1.5820	1.5550	1.4809	0.7383	0.7934	0.8326	0.6404	0.9783	0.6907	1.4514	1.2500	1.4972	1.4266	1.6305	1.5953	1.7798	1.4812	1.8856	2.0624	2.1160	2.2059	0.8686	0.7013	0.8880	0.8808	0.8627	1.3029
1-oleoylglycerol (18:1)	Lipid	Monoacylglycerol	21184	LC/MS Neg	100000924	1.7894	3.5884	2.0792	2.5166	2.4400	2.4463	2.9379	15.7591	0.8294	181.2963	1.8283	0.7302	0.6144	0.5845	0.6077	0.9637	0.4782	0.6471	0.6856	0.8455	3.7603	0.7579	0.8915	0.8798	0.9699	0.7982	2.0693	1.0301	1.2042	0.4717
1-oleoyl-GPC (18:1)	Lipid	Lysophospholipid	48258	LC/MS Pos Late	100001272	1.1154	1.6338	0.9663	0.6344	0.6876	1.3107	0.9030	1.0489	0.6448	0.9815	0.9586	0.6020	1.0474	0.7646	1.1884	1.0839	0.9208	1.0051	0.9758	1.0140	1.2115	0.6422	1.0149	1.1427	0.9982	0.8711	1.0409	0.8830	0.9856	0.9771
1-oleoyl-GPE (18:1)	Lipid	Lysophospholipid	35628	LC/MS Pos Late	100001569	1.0194	1.5565	1.1037	0.8432	0.8752	1.4471	0.9185	0.9943	0.7569	0.9338	1.0350	0.6095	0.9733	0.7577	1.0866	0.9852	0.8587	0.9260	1.0057	0.8886	1.8946	0.9481	1.1357	1.1965	1.0168	0.7573	1.0706	0.8756	1.0203	0.9469
1-oleoyl-GPI (18:1)	Lipid	Lysophospholipid	36602	LC/MS Neg	100001777	0.8629	0.8193	0.5687	0.8762	1.2346	1.0062	2.0809	1.5232	0.9903	3.1108	1.5367	1.1667	0.8276	0.7289	0.9328	0.9187	0.5696	1.1744	0.9821	1.6924	0.8671	0.7470	0.9956	0.9148	1.7681	1.4802	0.7452	1.4809	1.5045	0.7742
1-palmitoleoyl-2-linolenoyl-GPC (16:1/18:3)*	Lipid	Phosphatidylcholine (PC)	53180	LC/MS Pos Late	100009345	2.1169	2.1568	1.7429	1.6149	1.8431	2.3704	0.5832	0.6439	0.7107	0.5352	0.6975	0.4978	1.2627	1.0782	1.3989	1.5494	1.6363	1.3270	1.8869	2.1321	1.5439	1.3890	1.5235	1.5122	0.5299	0.6368	0.8980	0.7540	0.6600	1.1801
1-palmitoleoylglycerol (16:1)*	Lipid	Monoacylglycerol	52431	LC/MS Neg	100008952	0.7564	1.6111	1.0250	1.5324	0.2373	0.5391	0.9987	4.0964	0.2373	48.8336	1.7021	1.3313	0.5274	1.1314	0.4733	0.5448	0.8157	1.6649	0.5278	1.3990	2.8210	1.3248	0.8997	1.0064	0.6556	0.6485	0.3174	0.5328	0.2787	0.6299
1-palmitoleoyl-GPC* (16:1)*	Lipid	Lysophospholipid	33230	LC/MS Pos Late	100001511	1.2549	1.5854	1.1010	0.6126	0.6597	1.3846	0.7718	1.0625	0.5338	0.8568	0.7486	0.4504	1.2484	1.0920	1.5174	1.2127	1.1129	1.1510	1.3380	1.2474	1.4606	0.6566	1.1710	1.2157	0.8309	0.6669	1.1210	0.8653	0.9375	1.1564
1-palmitoyl-2-arachidonoyl-GPC (16:0/20:4n6)	Lipid	Phosphatidylcholine (PC)	52462	LC/MS Pos Late	100008914	0.6236	0.7113	0.6944	0.6504	0.6996	0.7418	1.1578	1.2579	0.9954	1.0965	1.1194	1.0248	0.8321	0.7926	0.8281	0.8328	0.8371	0.9190	0.7476	0.8127	0.6456	0.6151	0.8357	0.8037	1.2760	1.0582	1.0601	1.2464	1.0370	0.7951
1-palmitoyl-2-arachidonoyl-GPE (16:0/20:4)*	Lipid	Phosphatidylethanolamine (PE)	52464	LC/MS Pos Late	100008990	0.6611	0.7642	0.7315	0.7595	0.7489	0.7274	1.1255	1.2464	1.0565	1.0077	1.1150	1.0817	1.0066	0.9221	1.0562	0.9848	0.9853	0.9885	0.9810	1.0167	0.7532	0.7683	0.9697	0.9401	1.2158	1.1074	0.9934	1.1998	1.1755	0.9139
1-palmitoyl-2-arachidonoyl-GPI (16:0/20:4)*	Lipid	Phosphatidylinositol (PI)	52467	LC/MS Pos Late	100008993	0.5875	0.6585	0.8447	1.2212	1.2279	0.8591	1.3003	1.3384	1.0772	0.9837	1.1648	1.3104	0.8959	0.7403	1.0111	0.8592	0.8050	0.7674	0.9228	1.2455	0.8476	0.6140	0.8729	0.8894	1.5955	1.3782	1.1950	1.5748	1.3428	0.6904
1-palmitoyl-2-docosahexaenoyl-GPC (16:0/22:6)	Lipid	Phosphatidylcholine (PC)	52610	LC/MS Pos Late	100008915	0.5821	0.5570	0.5137	0.4522	0.4646	0.5095	1.0676	1.2997	0.9975	1.0500	1.1907	1.1849	0.8089	0.7918	0.7370	0.8293	0.8346	0.8503	0.7273	0.8457	0.5930	0.5865	0.8287	0.7742	1.2304	1.1023	1.0815	1.2867	1.1636	0.7138
1-palmitoyl-2-docosahexaenoyl-GPE (16:0/22:6)*	Lipid	Phosphatidylethanolamine (PE)	52465	LC/MS Pos Late	100008991	0.5277	0.5294	0.4885	0.3585	0.3743	0.4496	1.1002	1.1556	1.0967	1.0166	1.2165	1.1360	0.7908	0.7495	0.7593	0.8206	0.8651	0.8793	0.7562	0.9252	0.5202	0.5294	0.8109	0.8048	1.2528	1.1833	1.1727	1.3149	1.1678	0.7181
1-palmitoyl-2-gamma-linolenoyl-GPC (16:0/18:3n6)*	Lipid	Phosphatidylcholine (PC)	54812	LC/MS Pos Late	100009154	1.1534	1.3298	0.9380	0.8801	0.8118	0.9649	0.9080	0.8439	0.8740	1.0065	1.0194	0.7939	1.2090	1.2226	1.2844	1.5062	1.2198	1.1768	1.3273	1.8836	1.2415	1.1467	1.2459	1.2078	0.9406	1.0812	0.9127	0.9935	0.8688	1.1479
1-palmitoyl-2-linoleoyl-GPC (16:0/18:2)	Lipid	Phosphatidylcholine (PC)	42446	LC/MS Pos Late	1537	0.9933	1.2049	1.1888	1.0811	1.0985	1.1197	0.9187	1.0756	0.9038	0.9201	1.1211	1.1368	0.8710	0.8884	0.8557	0.8408	0.9999	0.9691	0.9354	0.9445	0.9285	0.8358	0.9581	1.0096	1.1406	1.0125	0.9718	1.1980	0.9644	0.8004
1-palmitoyl-2-linoleoyl-GPE (16:0/18:2)	Lipid	Phosphatidylethanolamine (PE)	42449	LC/MS Pos Late	100001870	0.9671	1.0949	1.2255	1.3675	1.2827	1.0585	0.9268	0.9020	0.9452	0.7597	1.0254	1.0514	0.9946	0.9475	1.1143	0.9353	1.0495	1.0054	1.0951	1.1325	1.0466	1.0132	1.2260	1.2788	1.1338	0.9697	0.8947	1.1613	1.0140	0.8946
1-palmitoyl-2-linoleoyl-GPG (16:0/18:2)	Lipid	Phosphatidylglycerol (PG)	52628	LC/MS Neg	1543	0.8322	0.6380	1.0763	1.1308	1.2171	1.2886	2.2239	2.0760	1.3507	1.3423	1.9577	1.2280	0.7076	0.3986	0.7864	0.6397	0.8230	0.7851	0.7841	0.7817	0.5811	0.2899	0.6836	0.6182	2.5991	1.9221	1.7035	1.8670	2.5163	0.8740
1-palmitoyl-2-linoleoyl-GPI (16:0/18:2)	Lipid	Phosphatidylinositol (PI)	52450	LC/MS Polar	1528	0.7798	0.8460	1.0299	1.1828	0.9149	0.7871	1.9016	1.5950	1.5163	1.5557	1.7329	1.6949	0.7118	0.6363	0.7264	0.6780	0.7285	0.5419	0.5900	0.8346	0.6184	0.5859	0.8727	0.8207	2.2399	1.8107	1.8751	2.0579	1.5019	0.7041
1-palmitoyl-2-oleoyl-GPC (16:0/18:1)	Lipid	Phosphatidylcholine (PC)	52461	LC/MS Pos Late	1539	0.8792	1.0587	1.0301	0.9373	0.9994	1.0163	0.9414	1.0006	0.8783	0.8664	1.0283	0.8806	0.9027	0.8497	0.9477	0.9493	0.9202	0.9759	0.9277	0.8876	0.8936	0.8243	0.9385	1.0334	1.1049	1.0070	1.0276	1.1338	0.9073	0.8163
1-palmitoyl-2-oleoyl-GPE (16:0/18:1)	Lipid	Phosphatidylethanolamine (PE)	19263	LC/MS Pos Late	1526	1.4583	1.2347	1.7960	2.4628	2.3055	1.6436	0.9331	0.7469	0.8916	1.0451	0.8564	0.7083	1.0201	0.8295	1.0947	1.1229	0.8630	1.0041	1.0133	0.9959	1.3727	1.6250	1.1785	1.4455	1.0102	0.9418	0.9163	0.8878	0.9638	0.8594
1-palmitoyl-2-oleoyl-GPG (16:0/18:1)	Lipid	Phosphatidylglycerol (PG)	52448	LC/MS Neg	100000641	0.6711	0.9453	0.8574	0.6835	0.7755	0.9696	1.2010	1.3716	1.0172	0.8028	1.0846	0.8591	1.0243	0.7168	0.9295	1.0077	1.1521	1.1647	0.9406	1.1815	0.8881	0.7869	0.8920	0.9973	1.2605	1.0932	1.1302	1.4296	1.3163	1.0027
1-palmitoyl-2-palmitoleoyl-GPC (16:0/16:1)*	Lipid	Phosphatidylcholine (PC)	52470	LC/MS Pos Late	100008984	1.0690	0.9670	0.9600	0.9764	0.9980	1.0018	0.9749	0.9245	0.8851	0.8847	0.8588	0.6805	1.6257	1.5030	2.2159	1.8276	1.4843	1.4807	1.7806	1.6750	1.6372	1.1376	1.3674	1.4585	0.8989	0.9590	0.9982	0.9656	0.8971	1.4572
1-palmitoyl-2-stearoyl-GPC (16:0/18:0)	Lipid	Phosphatidylcholine (PC)	52616	LC/MS Pos Late	100008921	1.0102	1.1035	1.0225	0.9294	0.7403	0.9088	1.0611	1.0032	1.0689	1.1190	0.9973	0.9908	0.8188	0.6509	0.6251	0.6998	0.6603	0.5231	0.8048	0.9304	0.6819	0.6421	0.6656	0.4840	1.0304	1.1260	0.9425	0.8080	1.0230	0.6270
1-palmitoylglycerol (16:0)	Lipid	Monoacylglycerol	21127	LC/MS Neg	100000827	1.8079	2.6949	2.1229	2.3490	2.6020	2.3077	1.2672	2.5808	0.7875	33.7734	1.2058	0.8291	0.6445	0.8256	0.7134	1.0974	0.6740	0.5956	0.6747	0.8109	2.0383	0.9930	0.5393	1.1417	0.9364	0.9149	1.2060	0.9120	1.1349	0.6389
1-palmitoyl-GPC (16:0)	Lipid	Lysophospholipid	33955	LC/MS Pos Late	100001263	0.9882	1.2477	0.9758	0.6976	0.7978	1.2740	1.0741	1.3146	0.8350	1.2063	1.1120	0.8635	0.9353	0.7400	1.0118	0.8990	0.7554	0.8254	0.8062	0.8723	0.9256	0.4454	0.8373	0.8723	1.2769	1.1079	1.2389	1.1891	1.2796	0.8869
1-palmitoyl-GPE (16:0)	Lipid	Lysophospholipid	35631	LC/MS Pos Late	100001567	0.7252	0.9812	0.8222	0.6182	0.6445	0.9689	1.0741	1.1223	0.8713	1.1414	1.3069	0.9048	0.9054	0.7805	1.0890	0.8981	0.8322	0.8927	0.8775	0.8741	1.3734	0.6278	0.8184	0.9565	1.2740	1.2229	1.1317	1.2069	1.2651	0.8936
1-palmitoyl-GPG (16:0)*	Lipid	Lysophospholipid	45970	LC/MS Neg	100005717	0.8562	1.5443	1.2892	0.5127	0.9169	2.1296	1.4199	1.3256	0.7668	1.6189	1.4394	0.7152	0.8520	0.6943	0.9827	0.9714	0.7473	1.0000	0.7229	0.6546	2.4185	0.6098	0.9116	0.6758	1.6857	1.3968	0.6841	1.2656	1.7849	1.2063
1-palmitoyl-GPI* (16:0)	Lipid	Lysophospholipid	35305	LC/MS Neg	100001655	0.8578	0.7562	0.7182	0.9485	1.3070	1.0616	1.8096	1.5039	0.9554	1.2235	1.5759	1.3459	1.0161	0.7656	1.4097	1.1391	0.6982	0.6595	0.9216	1.1076	1.0319	0.6301	0.7955	0.8597	1.9880	1.5446	0.9831	1.6530	2.0086	0.7665
1-palmitoyl-GPS (16:0)*	Lipid	Lysophospholipid	46130	LC/MS Neg	100005805	0.7425	1.4236	1.3684	0.7024	0.6151	2.1192	0.9877	1.0123	0.8787	0.6930	1.1859	0.9434	0.7311	0.5997	0.7959	0.6103	0.5598	0.5436	0.6902	0.7423	2.8680	0.7677	0.5694	0.6973	1.4725	1.0984	1.1054	1.2510	1.2706	0.5245
1-ribosyl-imidazoleacetate*	Amino Acid	Histidine Metabolism	61868	LC/MS Pos Early	100001668	2.1819	2.2016	3.4129	4.1408	3.8136	3.9350	0.9541	0.8233	0.5179	1.0459	0.7719	0.6968	1.5928	1.8650	2.4953	3.3794	3.8888	3.7062	3.7614	3.5281	8.1940	7.0489	5.4982	4.3652	1.5365	0.8862	0.4865	0.6187	0.8831	2.3357
1-stearoyl-2-arachidonoyl-GPC (18:0/20:4)	Lipid	Phosphatidylcholine (PC)	42450	LC/MS Pos Late	100001869	0.9433	0.9609	0.9730	0.9924	0.9924	1.0299	1.0612	1.0962	1.2038	0.9337	1.1395	1.1007	1.1165	1.0282	0.9173	0.9231	1.0676	1.0158	1.1414	0.8402	0.9521	0.7776	0.7993	0.9418	0.9539	1.0729	1.0697	0.9000	1.0515	0.8962
1-stearoyl-2-arachidonoyl-GPE (18:0/20:4)	Lipid	Phosphatidylethanolamine (PE)	52447	LC/MS Pos Late	100008977	0.8882	1.0460	0.9576	0.8741	0.9285	0.9268	0.9590	1.0809	1.0114	0.9217	1.1114	1.0393	0.9841	1.0029	0.9937	1.0199	1.0912	1.1421	0.9224	1.0089	0.7462	0.9201	1.0264	1.0260	1.0752	1.0143	0.8549	1.1133	0.9528	0.9428
1-stearoyl-2-arachidonoyl-GPI (18:0/20:4)	Lipid	Phosphatidylinositol (PI)	52449	LC/MS Pos Late	100000616	0.9006	1.0120	0.9040	0.7832	0.8318	0.9469	0.9388	1.0441	0.9356	0.7459	1.0737	0.9980	0.9995	0.8932	0.9960	1.0532	1.0624	1.0958	1.0005	1.0247	0.8759	0.8280	1.0242	1.0156	1.0638	1.0123	0.9720	1.0700	1.0344	0.9514
1-stearoyl-2-arachidonoyl-GPS (18:0/20:4)	Lipid	Phosphatidylserine (PS)	52235	LC/MS Pos Late	100001872	1.1067	1.1405	0.8915	0.8205	0.8865	1.0029	1.0983	1.2260	1.1749	0.8761	1.2449	1.1316	0.9146	0.7906	0.8057	0.8677	0.8443	0.8059	0.7623	0.9718	0.5604	0.5707	0.7717	0.8253	1.1430	1.2382	0.9781	1.1415	0.9998	0.7626
1-stearoyl-2-docosahexaenoyl-GPC (18:0/22:6)	Lipid	Phosphatidylcholine (PC)	52611	LC/MS Pos Late	100008916	1.0673	0.8530	0.8329	0.8970	0.8723	1.0246	1.0006	1.1165	0.9510	1.0886	1.0914	1.2206	1.0609	0.9462	0.9709	1.1789	1.0529	1.0882	0.9294	1.1336	0.7750	0.7775	0.9666	0.8892	0.9186	1.1753	0.9574	1.0165	1.0160	0.9232
1-stearoyl-2-docosahexaenoyl-GPE (18:0/22:6)*	Lipid	Phosphatidylethanolamine (PE)	52466	LC/MS Pos Late	100008992	0.8380	0.9456	0.7046	0.6431	0.6277	0.7719	0.8778	0.9338	0.9944	1.0190	1.0872	1.0505	1.0262	1.0658	0.9433	1.0583	1.1786	1.1830	0.9642	0.9770	0.5808	0.7632	0.9960	0.9934	1.2045	1.0642	0.9739	1.1296	1.0192	0.9238
1-stearoyl-2-linoleoyl-GPC (18:0/18:2)*	Lipid	Phosphatidylcholine (PC)	52452	LC/MS Pos Late	100008980	1.6062	1.7086	1.6549	1.6610	1.6707	1.6315	0.7881	0.9694	0.7668	0.9415	1.0779	1.1522	1.1222	1.0885	1.0819	1.1971	1.3462	1.2930	1.1302	1.1305	1.2434	1.2027	1.2749	1.3361	0.8128	0.8633	0.7563	0.8434	0.7549	1.0153
1-stearoyl-2-linoleoyl-GPE (18:0/18:2)*	Lipid	Phosphatidylethanolamine (PE)	52446	LC/MS Pos Late	100008976	1.8069	1.9583	1.7469	2.0845	2.0184	1.8450	0.6929	0.7201	0.8185	0.9365	1.1075	0.9046	1.5450	1.3667	1.5448	1.5608	1.7180	1.8438	1.5989	1.3641	1.5367	1.6300	1.7799	1.9149	0.8104	0.8868	0.7679	0.8432	0.8283	1.3932
1-stearoyl-2-linoleoyl-GPI (18:0/18:2)	Lipid	Phosphatidylinositol (PI)	52468	LC/MS Polar	100008994	2.5966	2.8667	3.2579	2.8940	2.7942	2.8811	0.8411	0.9790	0.9274	1.1707	1.1539	0.8060	1.1349	0.9535	1.2331	1.1576	1.1425	0.9356	1.0332	0.9042	1.0125	1.0859	1.6206	1.5907	0.8507	0.8573	1.3098	0.8325	0.6595	1.0911
1-stearoyl-2-linoleoyl-GPS (18:0/18:2)	Lipid	Phosphatidylserine (PS)	53009	LC/MS Pos Late	100008642	3.2960	2.7201	3.8769	4.4413	4.9156	4.8168	0.6583	0.7080	0.8303	1.3097	0.8181	0.9269	1.0297	0.8161	0.7856	1.0678	1.2259	1.2741	1.2631	0.9703	1.5266	1.1936	1.4121	1.5075	0.7464	0.8941	0.7571	0.7601	0.8248	0.8620
1-stearoyl-2-oleoyl-GPC (18:0/18:1)	Lipid	Phosphatidylcholine (PC)	52438	LC/MS Pos Late	100008904	1.7449	1.7730	1.7320	1.8685	1.9622	2.0616	0.7240	0.9013	0.7742	0.8824	1.0200	0.9379	1.2092	0.9366	1.2046	1.4586	1.5131	1.3470	1.0693	1.1078	1.0429	1.0518	1.1607	1.3504	0.7753	0.9108	0.7542	0.7489	0.7545	0.9979
1-stearoyl-2-oleoyl-GPE (18:0/18:1)	Lipid	Phosphatidylethanolamine (PE)	42448	LC/MS Pos Late	100001856	2.9120	2.5867	3.3757	7.1073	6.0055	4.4780	0.5734	0.5937	0.7630	1.3749	0.8184	0.5763	1.3483	0.9483	1.2167	1.5993	1.1466	1.6714	1.1705	0.9452	1.8017	2.5678	1.7478	2.3652	0.7732	0.8269	0.6366	0.5943	0.7309	0.9978
1-stearoyl-2-oleoyl-GPS (18:0/18:1)	Lipid	Phosphatidylserine (PS)	19265	LC/MS Pos Late	100000639	0.8019	1.1137	0.9996	1.3125	1.3186	1.0997	0.7544	1.0741	1.0549	0.8055	1.0637	0.8611	0.8027	0.6662	0.9222	1.0739	0.8667	0.9063	0.8565	0.9140	0.6645	0.7013	0.8787	0.8363	1.0441	1.0646	0.7776	0.8988	1.1406	0.8958
1-stearoyl-GPC (18:0)	Lipid	Lysophospholipid	33961	LC/MS Polar	100001271	1.8683	1.7913	1.4862	1.3047	1.4749	1.9945	0.8204	1.0644	0.7917	1.4024	1.1224	0.9943	1.2496	1.0474	1.1860	1.1607	1.0813	1.1488	0.8918	1.0595	0.8795	0.7441	0.9982	0.9914	1.0018	0.9842	0.9036	0.8354	1.0832	1.1201
1-stearoyl-GPE (18:0)	Lipid	Lysophospholipid	42398	LC/MS Pos Late	100001461	1.0949	1.6344	1.2117	0.9342	0.9651	1.6468	0.7991	0.9533	0.7276	1.2476	1.2701	0.7965	1.1402	0.9508	1.2164	1.1389	1.1062	1.1842	0.9675	0.9348	1.4928	0.7945	1.0117	1.0147	1.0123	0.9696	0.9216	0.9179	0.9761	1.0869
1-stearoyl-GPI (18:0)	Lipid	Lysophospholipid	19324	LC/MS Pos Late	100000656	0.5776	1.3558	0.9390	0.5971	0.4133	1.0221	0.9402	1.2595	1.0044	0.4535	1.5324	1.1826	1.0281	0.9956	0.7026	1.2289	1.2107	1.2717	0.9376	1.1870	0.4867	0.7091	1.0892	1.1522	1.2533	1.0085	1.0138	1.2356	0.6967	0.8593
1-stearoyl-GPS (18:0)*	Lipid	Lysophospholipid	45966	LC/MS Neg	100004327	1.3834	2.4274	2.4845	0.9404	0.9679	3.3539	0.7899	0.9646	0.8918	0.8235	1.7830	1.1306	1.0308	0.5967	0.9504	0.8990	0.7924	0.7872	0.8032	0.8000	3.4744	1.0907	0.6892	0.7076	1.1230	0.9566	0.9007	0.9315	1.1982	0.6288
2,3-dihydroxyisovalerate	Xenobiotics	Food Component/Plant	38276	LC/MS Polar	100002417	1.5990	0.8910	1.5880	0.9569	1.2692	0.7547	1.2075	0.9500	0.8724	0.6549	1.5942	1.6046	0.4694	0.4694	0.5649	0.4694	0.4976	0.8811	0.8316	0.4694	0.7796	0.4694	0.4694	0.4694	2.3273	0.4694	0.8139	1.4172	1.4210	0.4694
2,3-diphosphoglycerate	Carbohydrate	colysis, Gluconeogenesis, and Pyruvate Metabo	15664	LC/MS Neg	1227	0.7564	1.0201	0.7444	0.6943	0.2838	0.4629	1.3181	0.4692	0.6373	0.4718	0.2838	0.9511	1.7981	0.2838	1.0511	0.8770	0.6377	0.5567	2.1171	1.7993	2.2134	1.9580	0.9564	0.2838	0.2838	1.2438	1.0575	0.2838	0.7302	1.0422
2,4-dihydroxybutyrate	Lipid	Fatty Acid, Dihydroxy	62951	LC/MS Polar	100002285	1.3332	1.6904	3.2659	1.7053	2.3469	1.9976	0.6377	0.7448	0.7865	1.0770	0.9777	0.7512	1.1175	0.9033	1.3461	0.9359	1.1341	0.9718	1.0486	0.9740	1.1992	0.8359	1.4966	1.4594	1.0223	0.7516	0.8854	0.5492	0.7220	1.3646
2-aminoadipate	Amino Acid	Lysine Metabolism	6146	LC/MS Polar	381	3.3710	9.8215	10.7198	17.4916	20.5993	6.7175	0.2982	0.8868	0.9515	0.5089	0.4123	1.8756	1.3865	0.7615	0.4803	3.7434	0.4011	0.3490	0.9748	1.3246	0.1175	0.1384	0.5238	0.9044	1.0252	6.2570	4.7089	1.5904	0.5360	0.4534
2-aminobutyrate	Amino Acid	Glutathione Metabolism	42374	LC/MS Polar	1128	0.4223	1.2562	0.9631	1.9030	1.3106	0.7386	1.0436	1.2466	1.5510	0.8025	1.0042	0.8676	0.0491	0.0491	0.0491	0.2252	0.3017	0.1915	0.1425	0.2429	0.0491	0.0491	0.1980	0.1248	0.4143	1.0426	1.3484	1.2029	0.9056	0.1154
2-aminoheptanoate	Lipid	Fatty Acid, Amino	43761	LC/MS Pos Early	100004542	0.9776	0.5032	0.9914	0.8470	0.7118	0.5032	0.7224	0.9679	1.4397	0.5032	0.7006	0.9813	1.0804	0.9413	1.3180	0.9715	0.5972	1.6126	1.9526	0.7999	2.4449	1.8425	1.4760	2.9769	0.9208	1.4929	1.3137	0.5032	1.2238	1.3222
2-aminooctanoate	Lipid	Fatty Acid, Amino	43343	LC/MS Neg	100004227	8.9071	6.8663	9.8010	12.1223	10.7316	9.5551	0.8413	0.8515	0.5753	1.2445	1.2269	0.8886	1.8215	1.2784	1.8051	1.6797	1.1040	0.6513	2.0785	1.4792	0.8268	0.5753	1.0000	0.5753	0.9633	1.5744	0.5753	0.5753	0.8543	0.7774
2-arachidonoylglycerol (20:4)	Lipid	Monoacylglycerol	19266	LC/MS Neg	100000584	0.4626	0.9847	0.8150	0.4455	0.5464	0.3063	0.9143	2.7083	1.2885	20.3734	1.7886	1.2741	0.3063	0.7652	0.3063	0.3063	0.4906	0.5882	0.3063	1.1643	0.4162	0.3604	0.6185	0.4465	2.5327	1.9829	1.0867	1.7680	1.5337	0.4812
2-butenoylglycine	Lipid	Fatty Acid Metabolism(Acyl Glycine)	61785	LC/MS Neg	100016110	0.4587	0.7719	0.7334	1.0155	1.4013	0.4528	1.4139	1.2798	0.9578	1.3467	1.1533	0.5092	0.4528	0.4528	0.4528	0.4528	0.4528	0.4528	0.4528	0.4528	0.4528	0.4528	0.4528	0.4528	4.6924	1.0000	1.0819	0.8575	0.9737	0.4528
2'-deoxyadenosine	Nucleotide	Purine Metabolism, Adenine containing	1553	LC/MS Pos Early	211	1.0656	0.8994	0.6881	1.0000	0.7483	1.2082	0.4276	0.4276	0.8953	0.5757	0.4276	0.8058	1.0238	0.4276	1.0852	0.7329	0.6275	0.7281	0.4276	1.7013	0.4276	0.4276	0.4276	0.4276	1.3591	1.1493	0.4276	0.4276	1.8374	1.0989
2'-deoxyadenosine 5'-monophosphate	Nucleotide	Purine Metabolism, Adenine containing	46333	LC/MS Neg	100000485	1.2487	1.2088	0.9055	1.1764	1.1020	1.3360	1.1277	1.0380	1.1468	0.0882	1.0492	1.1564	1.3158	0.7345	0.7635	0.8477	0.7296	0.7184	0.7862	1.3525	0.6353	0.3779	0.6607	0.6193	1.4546	1.3125	0.9106	1.3642	1.4266	0.7588
2'-deoxycytidine	Nucleotide	Pyrimidine Metabolism, Cytidine containing	15949	LC/MS Pos Early	100000125	2.1265	2.5026	2.3647	2.3944	2.3172	2.4110	1.0194	0.9273	1.1313	0.4351	0.9985	0.9625	1.0440	0.6141	0.7329	0.6531	0.8633	0.6491	0.6818	1.1875	0.6530	0.5528	0.6462	0.5660	1.2026	1.1667	0.8369	1.1631	1.5392	0.6719
dCMP	Nucleotide	Pyrimidine Metabolism, Cytidine containing	533	LC/MS Pos Early	298	1.1748	1.2024	0.9856	1.1372	1.2446	1.4118	1.1594	1.0434	1.0144	0.4202	1.0199	1.1050	1.3331	0.6180	0.7284	0.8820	0.6493	0.7000	0.7605	1.5184	0.6210	0.3899	0.6354	0.6901	1.3547	1.2422	0.9115	1.3159	1.4338	0.7155
2'-deoxyguanosine	Nucleotide	Purine Metabolism, Guanine containing	1411	LC/MS Neg	348	0.9692	1.0666	0.8708	1.2492	0.9438	1.1666	1.0104	1.0719	1.1308	2.0892	0.9872	0.7653	0.9373	0.5791	0.8045	1.0482	1.0636	1.2259	0.7769	1.1376	0.7169	0.6441	0.9147	0.9896	1.3529	1.0456	0.8979	1.0695	1.3509	0.8967
2'-deoxyguanosine 5'-monophosphate (dGMP)	Nucleotide	Purine Metabolism, Guanine containing	35662	LC/MS Neg	100001546	1.2296	1.0869	0.8961	1.1774	0.9626	1.3605	1.2497	1.2736	1.0482	0.0822	1.1666	1.1865	1.2927	0.7377	0.7421	0.8416	0.8436	0.7932	0.8586	1.4768	0.5433	0.4027	0.7117	0.7233	1.5862	1.3134	1.0374	1.3748	1.4313	0.7866
2'-deoxyinosine	Nucleotide	ne Metabolism, (Hypo)Xanthine/Inosine contai	15076	LC/MS Neg	100000135	1.2416	1.4012	0.9155	1.1313	1.0074	1.1924	1.0278	0.9326	0.9926	1.1823	0.9742	1.0682	1.0545	0.5477	0.8255	0.8070	0.8687	0.6918	0.6049	1.1426	0.5477	0.4341	0.6617	0.6346	1.4603	1.2533	1.0127	1.1575	1.4507	0.6994
2'-deoxyuridine	Nucleotide	Pyrimidine Metabolism, Uracil containing	52602	LC/MS Neg	536	0.8343	1.1883	0.9206	1.1558	0.9782	0.9875	0.8783	1.1349	1.4917	0.6036	1.0671	1.0080	0.9920	0.5643	0.5859	0.8041	0.6397	0.5306	0.9000	1.6429	1.3632	1.2418	0.6324	0.8350	1.5096	1.0438	0.7544	1.1330	1.3407	0.6424
2-dihomo-linoleoylglycerol (20:2)*	Lipid	Monoacylglycerol	62450	LC/MS Neg	100020771	0.5161	0.5161	0.5161	0.5914	0.5161	0.5161	1.0724	1.9010	0.5161	20.4814	0.5161	0.5161	0.5161	0.5161	0.5161	0.5161	0.5161	0.5161	0.5161	0.5161	0.5161	0.5161	0.5161	0.5161	0.5161	0.5161	0.7573	0.5161	1.0000	0.5161
2-docosahexaenoylglycerol (22:6)*	Lipid	Monoacylglycerol	48675	LC/MS Neg	100006352	0.3770	0.5901	0.9997	0.2643	0.2643	0.3823	0.8247	3.0887	0.5542	22.2258	1.2930	1.0000	0.2643	0.2643	0.2643	0.3184	2.1301	0.8756	0.3222	1.7850	0.7441	0.4809	4.5594	0.4491	1.9594	1.1743	1.0663	1.2748	0.9515	0.2643
2-heptadecenoylglycerol (17:1)*	Lipid	Monoacylglycerol	62448	LC/MS Neg	100020770	0.7497	0.7497	0.7497	0.7497	0.7497	0.7497	1.0906	0.7497	0.7497	1.0000	0.7497	0.7497	0.7497	0.7497	0.7497	0.7497	0.7497	0.7497	0.7497	0.7497	0.7497	0.7497	0.7497	0.7497	0.7497	0.7497	0.7497	0.7497	0.7497	0.7497
2-hydroxy-3-methylvalerate	Amino Acid	Leucine, Isoleucine and Valine Metabolism	36746	LC/MS Neg	100001541	0.7654	0.7098	1.8159	1.5183	1.6254	1.1669	1.4952	1.5029	1.0671	0.4898	1.0901	1.5341	0.2796	0.2383	1.0508	0.2514	0.3393	0.4125	0.7075	0.8735	0.5560	0.4246	0.9860	0.6434	0.8123	0.9854	0.7807	0.6173	0.9396	0.2723
2-hydroxyadipate	Lipid	Fatty Acid, Dicarboxylate	31934	LC/MS Polar	100001153	6.1948	10.4579	16.8608	32.4568	35.6513	8.4332	0.9001	0.8470	0.8731	0.8048	0.8808	1.3849	1.4648	0.9842	1.0158	2.6319	0.6566	0.8513	1.3874	1.6779	1.1350	0.7222	1.5046	1.3032	1.8266	7.2331	4.9270	3.0107	1.3768	0.7052
2-hydroxybutyrate/2-hydroxyisobutyrate	Amino Acid	Glutathione Metabolism	52281	LC/MS Neg	100008928	0.5579	1.2802	1.4100	2.6839	1.8483	0.8568	1.2968	1.2966	1.1363	0.7600	1.0047	1.6144	0.3579	0.2427	0.2704	0.2645	0.2909	0.2976	0.2933	0.3807	0.4846	0.4275	0.7866	0.3502	0.7086	0.9953	1.7781	0.9727	0.7511	0.3304
2-hydroxyglutarate	Lipid	Fatty Acid, Dicarboxylate	37253	LC/MS Neg	100002070	1.8118	1.8122	1.7363	1.2699	1.5608	1.5190	0.9281	0.9269	0.8323	1.3939	1.4045	1.1749	0.7226	0.4112	0.6414	0.4629	0.4776	0.5138	0.4713	0.5271	0.3581	0.3353	0.6319	0.5545	1.3673	1.2736	1.5435	1.1796	1.0677	0.8151
2-hydroxyheptanoate*	Lipid	Fatty Acid, Monohydroxy	61827	LC/MS Neg	100019960	0.8325	0.8338	0.7806	0.6505	1.0204	0.5775	0.6124	0.6891	0.8267	0.4436	0.9728	0.9782	1.3311	1.2680	2.0492	1.2878	1.7171	1.7105	1.2552	0.9929	3.0231	1.4576	1.7502	2.2895	1.0017	1.5985	1.2136	0.9722	1.0142	1.7008
2-hydroxypalmitate	Lipid	Fatty Acid, Monohydroxy	35675	LC/MS Neg	100001579	1.3150	1.2721	1.5331	2.4117	2.3979	1.2712	1.0154	0.8654	0.9329	0.3272	0.9737	1.0103	0.9714	0.9433	0.9609	1.1590	1.0254	0.9822	1.0945	1.2621	1.2126	1.1456	0.9856	0.9536	1.3039	1.3128	0.9819	1.0681	1.2134	0.9765
2-hydroxyphenylacetate	Amino Acid	Phenylalanine Metabolism	1432	LC/MS Neg	235	1.4807	1.8359	1.6502	1.9864	3.1128	1.9587	1.7942	1.8774	3.0213	0.4600	2.0279	0.7624	1.3515	0.7727	0.7572	0.9991	1.3347	1.2790	0.9670	1.7320	0.8260	0.9411	0.9969	1.3005	1.1063	0.9929	1.0009	1.4453	1.9968	0.7769
2-ketogulonate	Carbohydrate	Fructose, Mannose and Galactose Metabolism	15306	LC/MS Neg	100000092	4.2803	4.6626	2.4766	2.2776	2.5715	4.7310	0.6803	0.7454	0.7736	1.0265	0.9997	0.7133	0.5439	0.5900	0.8586	0.9481	0.6961	0.8434	1.0123	1.1762	1.1268	0.5888	1.0349	0.9046	1.2045	1.4442	1.2555	1.6648	1.2229	0.6305
2-linoleoylglycerol (18:2)	Lipid	Monoacylglycerol	32506	LC/MS Neg	100000987	1.0106	1.9459	1.2608	1.2595	1.2182	0.7503	2.0414	5.3840	0.9131	40.2386	1.8130	0.9894	0.3845	0.5174	0.2575	0.4721	0.3320	0.4304	0.5154	1.0291	2.3311	0.5561	0.6001	0.5288	1.2427	1.2599	1.8675	1.3344	1.1633	0.3364
2-methylbutyrylcarnitine (C5)	Amino Acid	Leucine, Isoleucine and Valine Metabolism	45095	LC/MS Pos Early	100001509	0.5507	0.7177	0.3953	0.7887	0.7655	0.5171	1.2592	1.2793	1.3822	0.7673	1.1490	1.1239	0.9351	0.4495	0.4190	0.5437	0.4239	0.2901	0.4708	0.7173	0.4439	0.1931	0.3316	0.3879	1.0649	1.1289	1.2492	1.0696	1.4080	0.2354
2-methylbutyrylglycine (C5)	Amino Acid	Leucine, Isoleucine and Valine Metabolism	31928	LC/MS Neg	100001155	0.6636	0.7840	0.6703	1.0000	1.1023	0.7281	1.3208	1.3191	1.0276	0.7677	0.4814	1.0458	0.8543	0.5863	0.4531	0.5650	0.5025	0.3658	0.6816	0.8870	0.9774	0.4401	1.3469	0.3658	1.5039	0.8138	1.4123	0.7415	0.7338	0.5537
2-methylcitrate/homocitrate	Energy	TCA Cycle	52282	LC/MS Neg	100008929	1.4734	1.4370	1.2459	0.7069	1.2719	1.2100	1.7028	1.5955	1.4957	1.7886	1.9827	1.2804	0.4999	0.3287	0.3814	0.4223	0.3904	0.5606	0.3498	0.6960	0.2713	0.2477	0.4297	0.3391	2.5845	2.3947	1.8793	1.8971	1.9328	0.4357
2-myristoylglycerol (14:0)	Lipid	Monoacylglycerol	34383	LC/MS Neg	100001408	0.1946	0.1946	0.1946	0.1946	0.1946	0.1946	0.1946	0.1946	0.1946	1.8054	0.1946	0.1946	0.1946	0.1946	0.1946	0.1946	0.1946	0.1946	0.1946	0.1946	0.1946	0.1946	0.1946	0.1946	0.1946	0.1946	0.1946	0.1946	0.1946	0.1946
2-oleoylglycerol (18:1)	Lipid	Monoacylglycerol	21232	LC/MS Neg	100000943	1.0975	2.4338	1.4926	1.5953	1.6709	1.3946	2.6603	7.4106	0.6065	99.5746	1.4058	0.4695	0.3577	0.4948	0.3891	0.7471	0.3536	0.4061	0.4111	2.3704	2.7550	0.4233	0.5045	0.5283	1.0619	0.9938	1.9148	1.0062	1.0528	0.3920
2'-O-methyluridine	Nucleotide	Pyrimidine Metabolism, Uracil containing	57655	LC/MS Neg	100010896	1.4896	1.3868	0.5556	0.7088	0.4393	1.5074	1.4003	1.2122	1.0518	1.2400	1.5463	1.1349	0.7307	0.4509	0.6770	0.9391	1.0491	0.8206	0.4915	1.6907	0.2344	0.2344	0.4746	0.4341	1.1530	1.1389	0.9061	1.5494	1.2936	0.5920
2-oxoarginine*	Amino Acid	Urea cycle; Arginine and Proline Metabolism	55072	LC/MS Pos Early	100002784	1.0803	0.9838	1.4037	1.8245	1.6600	1.3656	0.5218	0.7645	0.4779	0.4057	0.5619	0.5787	1.1088	1.2806	1.0529	1.8061	2.1499	3.3612	1.0162	1.5315	3.9422	4.7976	3.4430	2.8856	0.7521	0.6166	0.6440	0.7232	0.7906	1.4401
2-palmitoleoylglycerol (16:1)*	Lipid	Monoacylglycerol	52432	LC/MS Neg	100008953	0.3394	0.3394	0.3394	0.3394	0.3394	0.3394	0.8821	2.1649	0.3394	25.6265	0.9240	0.3394	0.3394	0.3394	0.5744	0.3394	0.3394	0.3394	1.0000	0.4262	0.3394	2.2851	0.3394	0.3394	0.3394	0.3394	0.3394	0.3394	0.3394	0.3394
2-palmitoleoyl-GPC* (16:1)*	Lipid	Lysophospholipid	35819	LC/MS Pos Late	100001561	1.0494	1.0639	0.8045	0.6386	0.8233	1.1625	0.8932	0.8193	0.6396	0.7224	0.7058	0.6620	1.8137	1.4926	2.2068	1.9468	1.4641	1.4289	1.9816	1.6933	1.3968	0.8890	1.2141	1.1982	0.9687	0.9334	0.9196	1.1273	1.0880	1.3918
2-palmitoylglycerol (16:0)	Lipid	Monoacylglycerol	33419	LC/MS Neg	100001048	0.2824	0.9295	0.5418	0.2824	0.2824	0.2824	1.0705	0.2824	9.5508	0.2824	0.2824	0.2824	0.2824	0.2824	0.2824	0.2824	0.2824	0.2824	0.2824	0.5024	0.2824	0.2824	0.2824	0.2824	0.2824	0.2824	0.2824	0.2824	0.2824	0.2824
2-palmitoyl-GPC* (16:0)*	Lipid	Lysophospholipid	35253	LC/MS Pos Late	100001562	1.0622	1.1931	0.6862	0.6855	0.9604	1.4549	1.2737	1.3928	0.7928	1.0191	0.8893	0.8340	1.0852	0.7930	1.3956	1.1480	0.6007	0.5995	0.9577	0.8682	1.1259	0.4368	0.6292	0.7676	1.0006	1.0598	1.0073	1.1691	1.7714	0.9994
2-phosphoglycerate	Carbohydrate	colysis, Gluconeogenesis, and Pyruvate Metabo	35629	LC/MS Neg	100000093	0.9325	0.7657	0.8565	0.6591	0.5820	0.6008	1.3365	0.8133	0.9068	0.9898	0.8782	1.2392	1.3142	1.5168	0.9702	1.0958	1.1659	0.9322	1.5782	1.2035	1.6908	1.3998	1.0865	0.7878	0.7757	1.0412	1.1680	0.7904	0.9527	1.4016
2R,3R-dihydroxybutyrate	Lipid	Fatty Acid, Dihydroxy	63109	LC/MS Neg	100002367	0.8029	0.9720	1.5167	1.1779	1.2307	1.0620	0.8300	0.8582	0.9706	1.0277	0.9302	0.6103	0.8835	0.6892	0.6935	0.7189	1.1080	1.2499	0.4785	0.7628	1.4496	1.1801	1.1539	1.3494	1.3207	0.9125	0.8328	0.6224	1.2448	0.4899
2S,3R-dihydroxybutyrate	Lipid	Fatty Acid, Dihydroxy	62950	LC/MS Neg	100002284	0.0697	1.7388	2.0397	1.0222	1.3863	1.4967	1.1065	1.1205	1.1602	0.7796	0.8846	1.1547	0.4288	0.2836	0.3440	0.2885	0.4838	0.3838	0.6087	0.3209	0.2873	0.3186	0.4940	0.4120	1.2643	0.8236	1.0567	0.9778	0.6967	0.2441
2-stearoyl-GPE (18:0)*	Lipid	Lysophospholipid	41220	LC/MS Neg	100003901	1.0364	1.5566	1.4591	0.6406	1.0491	2.4622	0.6570	1.0605	0.5741	1.3100	1.8976	1.0785	1.0085	1.0804	1.8277	1.0043	0.9709	1.2399	0.7418	0.9115	1.2442	0.7800	1.1388	0.9660	1.1789	0.8414	0.4507	0.7528	1.0645	0.9957
3-(4-hydroxyphenyl)lactate (HPLA)	Amino Acid	Tyrosine Metabolism	32197	LC/MS Neg	240	0.7389	1.2030	1.0780	4.1830	2.6861	1.0398	1.7078	1.0125	0.8412	0.6876	0.6343	1.1538	0.7141	0.5489	0.6696	0.8142	0.7943	0.8036	0.8992	1.2106	1.1685	1.1398	1.2036	0.6591	1.0453	0.8678	0.9875	0.8955	0.7554	0.7648
3-amino-2-piperidone	Amino Acid	Urea cycle; Arginine and Proline Metabolism	62853	LC/MS Pos Early	100020361	0.6502	0.6991	0.7543	0.7554	0.6298	0.6085	0.9122	0.9694	0.9326	0.9142	1.0564	0.7930	0.9577	0.6408	0.8318	1.2266	1.1784	1.0720	0.9314	0.9270	1.3913	1.1478	1.4109	1.4611	1.0306	0.8745	1.3489	1.0665	1.1970	0.7557
3-aminoisobutyrate	Nucleotide	Pyrimidine Metabolism, Thymine containing	1566	LC/MS Pos Early	1114	0.9059	1.6323	1.5279	1.8600	2.1617	1.3805	0.8473	1.0131	1.1042	0.6058	0.9671	1.1113	0.4675	0.3075	0.4133	0.5461	0.6187	0.4797	0.6272	0.8152	0.8059	0.8728	0.7987	0.6532	1.0584	0.9869	0.8875	0.9209	0.9016	0.6055
3-dehydrocholate	Lipid	Secondary Bile Acid Metabolism	43835	LC/MS Neg	100001062	0.3670	0.4098	0.4122	0.2843	0.2843	0.2843	1.2826	1.6701	1.3677	0.2843	0.6375	0.8609	1.1490	0.3455	1.0378	2.5944	0.6158	2.0355	1.2240	0.5532	1.3927	0.4402	1.1527	0.7720	0.7522	1.9831	1.2755	1.0000	1.2790	1.0744
3'-dephospho-CoA-glutathione*	Amino Acid	Glutathione Metabolism	62807	LC/MS Neg	100020899	0.7268	1.0601	1.2425	0.6901	0.9109	0.9423	0.7251	0.6954	0.5566	0.3066	0.6960	0.7040	1.5083	1.0448	1.3725	1.5998	1.4820	1.7098	1.4638	1.2096	1.2763	1.9407	1.8791	1.5016	0.8454	0.9562	0.9886	0.9190	0.9660	1.3671
3-formylindole	Xenobiotics	Food Component/Plant	62863	LC/MS Neg	100020219	0.4091	0.5827	0.5987	0.6034	0.5152	0.4091	1.2314	0.8507	1.0213	0.4091	1.0227	0.9118	1.0558	0.7859	1.0003	0.7749	0.6761	0.8489	1.5617	0.8868	11.2125	0.9580	0.6594	0.4091	1.1098	0.8610	1.0208	0.9997	1.0897	0.4091
3-hydroxy-3-methylglutarate	Lipid	Mevalonate Metabolism	531	LC/MS Neg	112	1.3514	1.3334	1.4813	2.1963	2.3636	1.5587	1.0289	1.4885	1.5251	1.4476	1.9082	0.8823	0.1186	0.0841	0.1074	0.1052	0.1154	0.1784	0.0996	0.1384	0.0660	0.0632	0.1310	0.1008	1.0345	1.3065	1.4132	1.4141	1.2611	0.1113
3-hydroxyadipate*	Lipid	Fatty Acid, Dicarboxylate	62069	LC/MS Neg	100004396	1.6531	1.3433	2.3986	3.6786	3.2515	2.2301	0.8576	1.1473	1.1233	0.7653	1.3534	0.7539	1.1808	0.8070	1.0833	0.6652	0.7614	0.7977	1.1920	1.1384	1.1830	0.8544	1.0059	0.9941	0.7096	0.9170	1.6253	1.2333	0.9938	0.9170
3-hydroxybutyrate (BHBA)	Lipid	Ketone Bodies	542	LC/MS Polar	254	0.4598	0.6174	0.7431	0.8391	0.8798	0.3717	0.9734	1.6354	1.4415	0.8374	1.4338	1.2608	0.1168	0.0835	0.1181	0.1051	0.1237	0.1046	0.1252	0.1298	0.0902	0.0687	0.0967	0.0881	1.2488	1.5236	1.2149	1.3761	1.0802	0.0971
3-hydroxybutyrylglycine	Lipid	Fatty Acid Metabolism(Acyl Glycine)	62064	LC/MS Neg	100020211	0.6099	0.8967	0.7152	0.9264	0.8502	0.3577	1.5563	2.0848	1.5944	1.2686	1.8276	0.8362	0.1676	0.1024	0.1378	0.2069	0.1186	0.1057	0.0724	0.0563	0.1205	0.1232	0.1067	0.0578	1.5512	1.0996	1.7276	1.2050	1.0297	0.1151
3-hydroxydecanoate	Lipid	Fatty Acid, Monohydroxy	22053	LC/MS Neg	100000997	1.5879	1.4771	1.1398	1.3340	1.0169	1.0365	0.9695	0.8064	1.0055	0.8615	0.7290	0.8605	0.6796	0.8125	1.1554	1.0090	0.8371	1.0164	1.1235	0.9945	1.3545	0.8480	0.8750	0.8979	0.8476	0.9258	0.8006	0.8700	0.8699	0.9627
3-hydroxydodecanedioate*	Lipid	Fatty Acid, Dicarboxylate	62915	LC/MS Neg	100001454	4.6251	4.1602	14.0452	32.4676	26.4485	5.8949	1.1488	0.6276	0.5711	0.4648	0.4302	0.2888	1.4241	1.0469	1.1671	1.2610	0.9139	0.9757	1.0660	1.9126	1.6746	1.2549	0.9135	1.4722	0.4474	0.5667	1.0357	0.7861	0.6523	1.4991
3-hydroxyglutarate	Amino Acid	Lysine Metabolism	32373	LC/MS Neg	100001146	1.7309	1.8322	2.7640	4.5113	5.3581	3.0395	0.7728	1.2210	1.0966	0.8335	1.1270	1.1435	0.4125	0.2404	0.3561	0.5095	0.2919	0.3738	0.3936	0.3044	0.5565	0.4058	0.5996	0.4443	1.0167	1.2430	1.2526	1.3983	0.9833	0.3078
3-hydroxyhexanoate	Lipid	Fatty Acid, Monohydroxy	53230	LC/MS Neg	100006367	2.1962	1.8633	2.8920	5.0158	4.0837	3.0676	0.9004	0.6827	0.7992	0.4740	0.8088	0.7633	0.8188	0.8468	0.7139	0.4740	0.5151	0.5814	0.4740	0.6382	1.1162	0.9849	1.0835	1.1942	3.3596	1.0151	0.9429	0.9417	1.1939	0.6269
3-hydroxyhexanoylcarnitine (1)	Lipid	Fatty Acid Metabolism(Acyl Carnitine)	62557	LC/MS Pos Early	100020825	1.1296	1.1367	1.2107	1.5174	1.3626	1.1490	0.7861	0.5498	0.9872	0.3431	0.5462	0.9263	0.7656	1.0289	0.4281	0.6664	0.9658	0.9751	0.2856	0.8284	2.0020	1.0995	0.8867	1.3015	1.1729	0.9293	1.2729	1.0052	1.0778	0.5168
3-hydroxylaurate	Lipid	Fatty Acid, Monohydroxy	32457	LC/MS Neg	100001112	1.9409	1.8566	1.7549	1.9597	1.9689	1.3763	1.0339	0.9490	1.1381	0.6385	0.8911	0.6996	0.6931	1.0484	1.1125	0.9152	0.8094	1.1157	1.1329	0.7791	1.2669	0.7224	0.8188	0.8453	0.8061	0.8505	0.9574	0.9184	0.7912	1.3842
3-hydroxyoctanoate	Lipid	Fatty Acid, Monohydroxy	22001	LC/MS Neg	100001173	1.3421	1.3827	0.8817	1.1667	1.1970	1.0848	1.0118	0.7524	0.6713	0.7090	0.7071	0.7923	0.8264	0.6828	0.8672	0.5663	0.6058	1.0000	0.8481	2.0740	1.4488	0.5663	0.9301	1.0677	0.8187	0.6615	0.9454	0.9773	0.6702	1.0438
3-hydroxyoleate*	Lipid	Fatty Acid, Monohydroxy	61843	LC/MS Neg	100019966	0.8071	0.8620	1.0345	1.2758	1.7238	1.0966	0.6726	0.6381	0.5176	0.3465	0.7289	0.5330	1.1632	1.9249	2.3523	1.0991	1.0144	1.2300	1.4550	0.9609	1.4173	0.7307	1.5120	1.1759	0.8867	0.5857	0.5929	0.5857	0.6233	2.3969
3-hydroxyoleoylcarnitine	Lipid	Fatty Acid Metabolism(Acyl Carnitine)	61840	LC/MS Pos Late	100019801	0.5712	0.6924	0.9856	0.8616	0.7247	0.4082	1.2472	1.1476	0.9247	0.5478	1.6038	1.4935	1.4238	1.0144	1.2434	0.5882	1.2505	1.0579	1.0599	1.3722	1.4354	0.6968	0.8971	1.0732	0.5938	0.6067	0.9145	0.5388	0.4004	0.8458
3-hydroxypalmitate	Lipid	Fatty Acid, Monohydroxy	27503	LC/MS Neg	100000983	0.9120	1.1022	1.0647	1.3502	1.6291	1.1525	0.6716	0.7207	0.5062	0.4800	1.0440	0.8001	1.2835	2.0910	2.6188	1.2095	1.3808	1.5338	1.7708	0.7594	1.9568	1.1658	0.9365	1.2042	0.8361	0.7518	0.5938	0.8548	0.7025	2.2469
3-hydroxypalmitoylcarnitine	Lipid	Fatty Acid Metabolism(Acyl Carnitine)	61839	LC/MS Pos Late	100019800	0.7272	0.9655	1.1190	1.5301	1.3366	0.9496	0.7917	0.8832	0.8683	1.3964	1.6034	1.3184	1.1431	1.0092	1.6482	0.5733	0.9688	1.0129	1.0000	1.4372	2.0449	1.0395	0.7457	1.2450	0.7298	0.6845	0.7811	0.5610	0.4946	0.6933
3-hydroxysebacate	Lipid	Fatty Acid, Dicarboxylate	31943	LC/MS Neg	100001145	0.9821	0.9318	1.7730	5.3650	2.9550	1.0505	1.0179	0.6395	1.1105	0.4515	0.6463	0.3801	1.5207	0.9742	1.1078	0.9346	0.8688	0.8768	1.2441	1.7166	1.9762	1.3412	1.0292	1.9120	0.8505	0.6324	1.0761	0.8616	0.6857	1.1248
3-hydroxysuberate	Lipid	Fatty Acid, Dicarboxylate	31936	LC/MS Neg	100001143	2.1821	1.4178	2.4148	5.1491	3.9759	2.0238	0.5635	0.6865	0.6039	0.7018	0.9748	0.6469	3.7126	2.8175	2.9548	2.0449	2.5887	2.4311	3.3270	4.0940	2.1458	1.6003	2.1873	2.8332	0.5721	0.6211	0.9991	1.0009	0.7629	2.5571
3-hydroxytetradecanedioate	Lipid	Fatty Acid, Dicarboxylate	31930	LC/MS Neg	100001147	3.9242	4.1428	9.1953	44.3592	21.1870	4.4168	0.9319	0.6118	0.5112	0.2200	0.5392	0.3153	2.6784	1.7204	2.5048	1.6021	1.0830	1.1972	1.9169	2.8799	2.1705	1.3188	0.8270	1.2989	0.3420	0.3867	0.8909	0.4972	0.6265	3.4071
3-indoxyl sulfate	Amino Acid	Tryptophan Metabolism	27672	LC/MS Neg	100000467	0.4684	0.5438	1.9789	1.2786	1.8084	0.4760	1.3405	1.0022	0.7520	1.7135	1.3458	1.1386	0.4745	0.3627	0.3983	0.6879	0.7324	0.3918	0.8421	0.3077	0.1277	0.1194	0.5853	0.4295	3.8295	1.9341	0.9395	0.8338	1.3345	0.2446
3-methylcrotonylglycine	Amino Acid	Leucine, Isoleucine and Valine Metabolism	31940	LC/MS Pos Early	100001149	0.9071	1.2278	0.8326	1.5887	1.1953	1.0990	1.4261	1.3890	0.9095	0.4688	1.1873	0.8053	1.3098	0.6657	0.5713	0.7267	0.7811	0.9342	1.0597	1.5640	1.1796	0.6568	1.0255	0.8574	1.3530	1.1080	0.8559	1.1051	1.1107	0.7554
3-methylcytidine	Nucleotide	Pyrimidine Metabolism, Cytidine containing	35132	LC/MS Pos Early	100001466	0.8713	1.6875	1.4425	1.3401	1.3621	1.4381	0.8703	0.9881	0.6886	0.5713	0.6275	0.8404	1.2067	1.0695	1.3128	1.1957	1.3212	0.9992	1.4942	1.9305	1.6654	1.6587	1.4380	1.1967	0.9557	0.8226	0.7842	0.9792	1.0008	0.9921
3-methylglutaconate	Amino Acid	Leucine, Isoleucine and Valine Metabolism	57747	LC/MS Neg	100002458	0.3459	0.7445	0.9012	0.9335	1.0693	0.7014	1.3309	1.4732	1.1814	1.5042	1.8952	1.1844	0.5837	0.3929	0.5159	0.3459	0.3459	0.5212	0.4750	0.4128	0.4166	0.4407	0.4131	0.3878	2.9566	1.9330	1.2239	1.7046	1.3561	0.3875
3-methylglutarate/2-methylglutarate	Lipid	Fatty Acid, Dicarboxylate	54676	LC/MS Neg	100010834	0.6342	1.5303	2.0250	2.6293	2.8566	2.3959	0.6435	0.6571	1.2812	0.4135	1.1832	0.4135	1.8160	0.9798	1.9447	1.9260	0.6495	1.7422	0.8650	1.3439	2.2973	2.5219	2.3599	4.7125	0.6745	0.7219	0.5970	0.4135	0.5830	0.8512
3-methylhistidine	Amino Acid	Histidine Metabolism	15677	LC/MS Pos Early	100000042	0.3829	1.0046	0.2925	0.3621	0.2925	0.3116	1.3613	1.3054	1.3278	1.4993	0.9778	0.6908	1.0954	0.8105	0.8964	1.1353	0.9759	0.4585	0.9954	0.4652	0.4505	0.4715	0.6582	0.2925	1.0629	1.0529	1.2855	0.9063	0.8167	0.5604
3-phosphoglycerate	Carbohydrate	colysis, Gluconeogenesis, and Pyruvate Metabo	1414	LC/MS Neg	132	0.7625	0.7258	0.6909	0.5774	0.4514	0.5552	1.2140	0.8205	0.9432	0.8396	0.9057	1.0435	1.2345	1.1208	0.9837	0.9910	1.0877	0.8755	1.3339	1.2649	1.5556	1.3091	1.1487	0.7369	0.8575	1.1028	1.0606	0.8740	1.0144	1.1381
3-ureidopropionate	Nucleotide	Pyrimidine Metabolism, Uracil containing	3155	LC/MS Pos Early	1053	0.7415	0.4518	0.5697	0.4701	1.2370	1.1427	0.5945	0.4517	0.4713	0.9911	1.0089	0.6317	2.3152	2.6941	1.3103	2.5998	1.3467	1.4097	1.5888	1.4888	7.5493	5.8133	0.9529	6.4419	0.6873	1.0749	0.4361	0.8347	0.4129	2.1385
4-cholesten-3-one	Lipid	Sterol	38125	LC/MS Pos Late	100002227	0.7289	0.8420	0.4594	0.6677	0.4996	0.7976	0.7114	0.4594	0.8282	0.4897	0.8519	0.5278	1.6290	1.6665	1.8430	1.8732	1.9172	1.6599	1.4181	0.6523	1.3120	1.1697	1.1628	1.1293	0.8486	1.3685	0.7334	0.5758	0.8473	2.2663
4-guanidinobutanoate	Amino Acid	Guanidino and Acetamido Metabolism	15681	LC/MS Pos Early	100000096	6.7093	2.5581	2.3519	2.8179	3.0981	2.3793	0.5509	0.5165	0.6016	0.4093	0.5157	0.4522	1.2368	0.6177	1.0510	2.2131	1.6604	2.4277	1.6920	2.9546	2.5475	1.7051	2.8658	2.0483	1.5889	1.3102	0.9490	0.7831	0.6187	2.7131
4-hydroxycinnamate sulfate	Amino Acid	Tyrosine Metabolism	48410	LC/MS Neg	100006095	4.2435	9.3119	7.8213	0.9168	0.2368	7.1637	0.1684	0.1684	0.1684	0.1684	1.5616	0.2645	0.2574	0.1684	0.1684	0.1791	1.2262	0.1684	0.2120	0.1684	1.4410	0.1684	1.1077	0.9202	1.0798	0.5216	0.1684	0.1684	0.1684	0.1684
4-hydroxyglutamate	Amino Acid	Glutamate Metabolism	40499	LC/MS Pos Early	100002544	1.3815	1.7562	1.3971	0.7143	1.0297	1.9718	0.3876	0.7996	0.9699	1.5693	0.8137	0.3876	0.9981	0.8095	1.0731	1.5626	0.6767	0.6877	1.0251	1.0019	0.6053	0.5835	0.8330	1.2336	0.6148	1.5842	0.3876	0.7379	0.3876	0.8437
4-hydroxynonenal	Lipid	Fatty Acid, Oxidized	143	LC/MS Pos Early	100000449	0.8939	1.2564	0.5534	0.5322	0.5534	0.4814	0.1939	1.2914	0.3673	1.2343	0.3673	0.7132	0.5663	0.9282	1.0454	1.1103	0.9450	1.4625	1.4976	16.0900	0.9540	1.9440	1.5662	1.2760	0.8933	1.8956	1.4117	1.5966	1.5779	0.7318
4-hydroxy-nonenal-glutathione	Amino Acid	Glutathione Metabolism	48487	LC/MS Neg	100006240	0.7915	0.7377	0.8282	0.2734	0.9830	0.8493	0.4703	0.4221	0.5216	0.0437	0.8438	0.8629	1.1039	0.8069	1.4325	1.3124	1.4063	1.9167	1.3738	0.5785	0.8278	1.1118	1.5333	3.0550	1.0984	1.4706	1.0000	0.8375	0.9337	1.9440
4-hydroxyphenylpyruvate	Amino Acid	Tyrosine Metabolism	1669	LC/MS Neg	1141	0.5064	0.6311	0.5064	0.9124	0.5064	1.4568	0.5064	1.4138	0.6885	0.5064	0.5064	0.5064	1.5328	0.5064	0.5736	0.5064	1.3090	0.6067	0.5064	0.5064	0.6314	1.0241	1.4483	0.5064	1.3105	0.8152	1.5360	1.1847	1.0000	0.5064
4-imidazoleacetate	Amino Acid	Histidine Metabolism	32349	LC/MS Pos Early	100001207	0.7723	1.0824	1.2075	2.1766	1.8276	1.2705	1.0391	1.0036	0.8674	1.1272	0.9579	0.7370	0.6451	0.6377	0.8698	0.6811	1.2231	1.2917	1.2810	0.8677	1.9575	1.6848	1.2591	1.4379	1.8091	0.9964	0.6597	1.0281	0.7554	0.8436
4-methylhexanoylglycine	Lipid	Fatty Acid Metabolism(Acyl Glycine)	62736	LC/MS Neg	100020632	0.1895	0.6841	0.4271	0.4641	0.5400	0.4532	1.6795	0.9313	1.1061	1.2069	0.8619	1.2393	0.2905	0.1895	0.3734	0.5147	0.1895	0.3889	0.5492	0.5661	0.1895	0.1895	0.8093	0.1895	3.3456	1.3640	1.5788	1.5730	0.7358	0.1895
5-(galactosylhydroxy)-L-lysine	Amino Acid	Lysine Metabolism	43582	LC/MS Pos Early	100002462	1.3795	1.4918	1.4083	1.1738	1.0133	1.3500	0.8601	0.9965	0.7822	1.2982	1.0852	0.9073	1.3026	0.7547	1.1525	1.3478	0.8742	1.0016	0.9205	1.1165	1.0721	1.1718	0.9388	0.9702	1.0651	0.9984	0.9259	0.7628	0.8811	0.8586
5,6-dihydrouridine	Nucleotide	Pyrimidine Metabolism, Uracil containing	61833	LC/MS Neg	100001425	1.0147	1.5186	1.6109	1.4369	1.4114	1.7979	1.2338	1.1281	0.7690	1.3781	0.8163	0.6794	1.0846	0.7801	1.0703	1.1526	0.8432	0.7744	0.9037	1.3137	0.6759	0.5057	0.6563	0.6356	2.4245	1.2144	0.6540	0.9853	1.1226	0.6372
5-aminovalerate	Amino Acid	Lysine Metabolism	18319	LC/MS Pos Early	100000454	0.8813	0.2952	0.8113	0.9934	1.1061	0.6908	0.8601	0.9644	1.2110	0.3613	1.0353	0.9029	0.9275	0.7623	1.1227	0.7781	0.9792	1.1843	1.0765	0.5913	0.8606	1.1452	1.6179	1.7740	1.0469	1.1585	0.9989	0.9294	1.0011	0.9878
5-dodecenoate (12:1n7)	Lipid	Medium Chain Fatty Acid	33968	LC/MS Neg	100001232	0.9330	1.0635	0.6299	0.2847	0.3710	0.6418	0.7962	0.9006	0.8150	4.5577	1.1382	0.5984	1.6906	1.4607	2.2749	1.6787	1.5573	1.6384	2.2893	1.5586	1.9462	1.1865	1.5359	2.0012	0.4543	0.5333	0.9152	0.7803	0.5534	1.3332
5-hydroxyhexanoate	Lipid	Fatty Acid, Monohydroxy	31938	LC/MS Neg	100001148	1.6554	1.8128	2.4398	3.6525	3.0796	1.9107	0.7076	0.9105	0.9848	0.4806	0.8625	2.3389	0.7430	0.8214	0.8875	0.5091	0.4806	0.2960	0.7920	1.0464	1.1170	1.0225	0.7919	0.7263	0.9827	1.2343	1.0144	0.4806	0.9150	0.8323
5-hydroxyindoleacetate	Amino Acid	Tryptophan Metabolism	437	LC/MS Pos Early	71	0.9562	1.1282	0.7144	0.8473	0.2354	0.3920	1.3551	0.8950	0.7561	1.1504	1.0000	0.9220	1.1381	1.2356	1.1897	0.9577	1.1830	0.7745	2.1674	1.2298	0.2354	1.3073	1.7329	0.9204	0.8622	0.8614	0.7887	0.9296	0.8428	1.1510
5-methyl-2'-deoxycytidine	Nucleotide	Pyrimidine Metabolism, Cytidine containing	38159	LC/MS Pos Early	100002300	1.1675	1.3115	1.3587	1.6254	1.6031	1.5660	0.9420	1.0050	1.1323	0.2334	1.0231	0.8940	1.0885	0.4147	0.6771	0.7469	0.7620	0.6030	0.6242	1.3054	0.5324	0.4542	0.6448	0.5429	1.4578	1.0898	0.9699	1.0060	1.4604	0.6964
5-methylcytidine	Nucleotide	Pyrimidine Metabolism, Cytidine containing	22119	LC/MS Pos Early	100000763	2.7350	2.3552	2.7631	3.5003	3.3603	3.1568	1.5560	1.4768	1.4658	1.0550	1.1734	1.2980	0.7293	0.7326	0.6172	0.7445	0.7235	0.5972	0.6659	1.0156	0.5515	0.6248	0.6070	0.6143	1.3414	1.2736	1.1258	1.4872	1.6993	0.5045
5-methyltetrahydrofolate (5MeTHF)	factors and Vitami	Folate Metabolism	18330	LC/MS Neg	1244	0.2663	0.5392	0.6332	0.3717	0.9614	1.0000	0.4994	1.0479	0.4217	1.6832	1.5668	1.3711	0.6741	0.3199	0.5507	1.6118	0.6772	1.6686	0.1056	1.4271	0.1056	0.1132	1.2830	2.9102	3.6325	2.2138	2.2431	2.1184	2.2289	1.0875
5-methylthioadenosine (MTA)	Amino Acid	Polyamine Metabolism	1419	LC/MS Pos Early	212	0.4308	0.4071	0.6093	0.5789	0.5451	0.5181	0.6675	0.7487	0.6083	0.8967	1.0775	0.9545	0.9080	0.8799	2.2625	1.2525	0.9486	1.0602	1.5083	0.7809	0.8492	0.9193	0.9911	1.4809	1.4705	1.2139	1.2361	1.0505	1.0089	1.3699
5-methyluridine (ribothymidine)	Nucleotide	Pyrimidine Metabolism, Uracil containing	35136	LC/MS Neg	100001446	1.1474	1.3998	0.7083	0.7485	0.6547	1.2309	1.2610	1.2167	0.8551	0.8364	1.1802	1.2376	0.8601	0.6640	0.7256	0.7723	0.7775	0.6983	0.7362	0.8821	0.9114	0.8657	0.8002	0.5853	1.6199	1.4014	1.1179	1.5427	1.7734	0.6471
5-oxoproline	Amino Acid	Glutathione Metabolism	1494	LC/MS Neg	1021	0.9668	1.0988	0.9424	0.6874	0.8189	0.8806	1.0527	1.0287	1.0500	1.2093	1.1418	1.0595	0.8112	0.5844	0.7885	0.7808	0.7538	0.7343	0.7938	0.9933	0.5688	0.4498	0.7695	0.7331	1.0026	1.1798	1.2017	1.1807	0.9974	0.6891
5-phosphoribosyl diphosphate (PRPP)	Carbohydrate	Pentose Phosphate Pathway	36840	LC/MS Neg	166	0.3623	1.1175	0.5363	0.3623	0.5263	0.9039	1.6103	0.7153	0.9411	0.3623	0.6263	1.4100	1.2582	1.0183	0.3623	0.3623	0.5868	0.5063	1.1229	1.2751	1.2866	1.8382	0.8614	0.3623	0.9157	0.9570	0.9150	0.8191	1.3720	0.5672
6-beta-hydroxylithocholate	Lipid	Secondary Bile Acid Metabolism	36807	LC/MS Neg	100001625	0.0522	0.8539	0.2283	0.0542	0.0741	0.0342	3.9939	1.2403	1.5586	0.3519	0.4485	1.7298	1.2497	0.3462	0.7719	0.8703	0.4545	1.7330	1.9270	0.2093	2.1313	0.5457	1.2699	1.3801	1.5734	3.3212	1.5528	0.8756	1.7124	0.8755
6-keto prostaglandin F1alpha	Lipid	Eicosanoid	20476	LC/MS Pos Late	100000571	0.7409	1.3333	0.8748	1.4808	0.4065	0.4065	1.2724	1.7501	2.0307	1.2584	0.9318	0.8647	2.5341	0.5149	0.7711	0.6704	0.5191	0.4065	0.4065	0.7506	0.5992	0.4305	0.6392	0.4065	1.5666	1.0989	1.0946	1.6218	2.5570	0.4786
6-oxolithocholate	Lipid	Secondary Bile Acid Metabolism	46336	LC/MS Neg	100001068	0.1510	0.2446	0.1510	0.1510	0.1510	0.1510	5.1597	0.8275	1.4376	0.1832	0.3107	0.7716	0.4452	0.1510	0.2943	0.6621	0.4025	1.4495	1.5612	0.5110	3.7551	0.3801	1.5069	0.8070	1.1452	3.3700	2.0087	1.0000	1.9241	0.5593
6-oxopiperidine-2-carboxylate	Amino Acid	Lysine Metabolism	43231	LC/MS Pos Early	100004499	6.2401	7.1163	19.4816	27.2658	21.1825	11.0273	0.3393	0.3892	0.4679	0.3755	0.8153	0.6731	0.3314	2.4114	0.9683	5.6489	0.9945	1.1636	2.2798	2.1513	0.3996	0.3320	1.5615	1.7938	0.6008	1.2877	1.0000	0.6849	0.7474	1.2058
6-phosphogluconate	Carbohydrate	Pentose Phosphate Pathway	15442	LC/MS Neg	100000341	1.0759	1.0495	1.3200	1.2876	1.2729	1.4256	0.8328	1.0826	0.8152	0.5572	1.1422	1.3635	0.8214	0.5483	0.7081	1.1394	0.9537	0.7990	0.6367	1.1574	0.6343	0.5715	0.8475	0.9677	1.6898	1.6847	1.1112	1.1808	1.6128	0.7640
7,8-dihydrofolate	factors and Vitami	Folate Metabolism	1708	LC/MS Neg	173	0.6321	0.8947	1.4494	0.8290	1.0558	0.8028	1.7021	1.5106	1.1866	0.6741	1.7519	1.6295	0.9861	0.8636	1.0000	1.3982	1.1616	1.0834	0.6675	1.1995	0.2201	0.8005	0.7611	0.7164	1.2027	0.8762	1.3808	2.1851	1.7052	0.9143
7-HOCA	Lipid	Sterol	36776	LC/MS Neg	100001609	0.8824	0.9756	1.9423	0.9849	0.3507	1.3785	1.1295	0.7861	1.6884	0.3208	1.7462	0.8441	0.4171	0.3231	0.6617	1.5427	0.7690	0.7940	0.6918	1.0285	0.2957	0.2957	0.5552	0.5345	1.2490	1.3489	1.1352	1.2727	1.6455	0.7420
7-hydroxycholesterol (alpha or beta)	Lipid	Sterol	47890	LC/MS Pos Late	100005999	0.7358	0.6216	0.5082	0.5005	0.4354	0.3513	0.7425	0.4789	0.8606	0.3513	0.9776	0.9282	0.8743	0.9843	1.0760	0.8233	1.0115	1.0913	1.4576	0.5560	2.4200	1.4059	1.0956	1.7355	1.4611	2.1088	1.0691	1.4160	1.2088	0.8263
7-ketodeoxycholate	Lipid	Secondary Bile Acid Metabolism	31904	LC/MS Neg	100001063	0.2471	0.9171	0.4984	0.4214	0.2788	3.1815	1.9518	1.8653	1.7863	0.6831	0.3497	1.4658	1.3154	0.1628	1.0161	2.9726	0.3446	1.8504	2.1318	0.7024	0.3135	0.3660	2.9701	0.7649	1.7977	1.3399	0.9839	1.2952	1.6382	0.4102
7-methylguanine	Nucleotide	Purine Metabolism, Guanine containing	35114	LC/MS Pos Early	100001456	1.0676	1.4344	1.4415	1.7455	1.9366	1.4969	1.2620	1.0021	1.0727	2.0766	0.7476	0.8900	0.8814	0.7377	0.8563	0.7094	0.8694	0.5132	0.6556	0.8385	0.8090	0.5132	0.5694	0.9687	2.6389	0.8552	0.7656	1.4450	1.2819	0.7123
9,10-DiHOME	Lipid	Fatty Acid, Dihydroxy	38399	LC/MS Neg	179	4.5809	3.5692	3.5932	4.6260	4.0814	3.6117	0.8918	0.9852	0.6392	2.5370	0.8988	0.5976	1.0082	0.8330	1.1468	0.9918	1.1136	1.1133	1.1589	1.2162	1.8725	1.2442	1.2063	1.7835	0.6000	0.6151	1.1420	0.6840	0.7743	0.9150
acetylcarnitine (C2)	Lipid	Fatty Acid Metabolism(Acyl Carnitine)	32198	LC/MS Pos Early	100000802	0.8600	0.5812	0.8341	1.2942	0.8509	0.4998	0.9934	1.2490	1.4832	5.6255	1.3855	1.4654	1.7350	1.0777	0.7220	1.1086	1.0066	0.7416	0.7325	1.6896	0.9050	1.0304	0.8454	0.9141	0.9893	1.3420	0.6520	0.8642	0.8081	0.4559
acetylphosphate	Energy	Oxidative Phosphorylation	15488	LC/MS Polar	1211	1.3722	0.6399	0.9090	1.0000	1.4607	1.0869	1.3634	0.4591	0.8840	3.5038	0.5393	0.9957	1.2000	1.2505	0.8537	1.4056	1.1753	0.7276	1.2719	0.9884	1.4039	0.4591	0.5134	1.0451	0.5829	0.7913	1.4433	1.0027	0.9637	1.2198
aconitate [cis or trans]	Energy	TCA Cycle	46173	LC/MS Polar	100001359	33.8297	23.7049	47.5206	94.7948	54.1529	64.6106	0.7311	0.4342	0.6693	2.7661	2.3565	0.4437	0.8571	0.3406	0.5815	0.7070	1.3856	0.3855	3.5468	0.7384	13.8625	10.0872	3.5388	0.5905	0.7011	1.0011	0.5226	0.6917	0.7308	0.8180
adenine	Nucleotide	Purine Metabolism, Adenine containing	554	LC/MS Pos Early	880	0.6218	0.6428	0.5689	0.3963	0.4355	0.6404	0.8975	0.8486	0.6646	0.7840	0.6835	1.0849	1.0690	1.1102	1.4197	0.9308	0.9159	0.9123	1.1489	1.2263	0.9919	1.3054	1.4057	0.6906	1.2711	1.0786	1.0081	1.2140	1.0281	1.4509
adenosine	Nucleotide	Purine Metabolism, Adenine containing	555	LC/MS Pos Early	798	1.1188	0.9659	0.9842	0.8862	0.9342	1.2642	1.0569	1.0480	0.7631	0.6752	1.0916	0.8353	1.2149	0.7235	1.2440	0.8459	0.7845	1.0336	1.2061	1.6508	0.6333	0.5735	1.0158	0.4286	1.2101	1.7045	0.7311	1.0393	1.6098	1.1435
2'-AMP	Nucleotide	Purine Metabolism, Adenine containing	36815	LC/MS Neg	100001694	0.9212	0.8166	0.8796	0.5589	0.8673	0.6803	0.7779	0.6120	0.7035	1.0387	0.6292	0.8456	1.4009	1.0231	1.3151	1.4559	1.4025	1.1192	1.7152	1.3382	1.6853	1.6221	1.2490	1.2112	0.9225	0.9159	0.7041	0.6725	0.7371	1.1332
adenosine 3',5'-cyclic monophosphate (cAMP)	Nucleotide	Purine Metabolism, Adenine containing	2831	LC/MS Neg	207	0.8696	1.0903	0.5652	0.7661	1.0782	0.9216	0.8780	0.8868	0.6729	1.3891	1.0379	1.2664	0.9487	0.5652	0.7353	1.0410	1.0088	0.8484	0.6689	0.6208	0.9930	0.6490	0.7993	0.6600	1.3428	1.5251	0.9735	1.1988	1.0995	0.5652
adenosine 3',5'-diphosphate	Nucleotide	Purine Metabolism, Adenine containing	15691	LC/MS Neg	1214	1.4053	0.6143	2.2196	0.7312	2.1806	0.5279	0.2678	0.2765	0.1416	0.1989	0.2098	0.8961	1.1566	1.2567	1.3333	1.0452	0.8294	0.6307	3.1064	0.2845	2.4966	2.2262	2.7314	1.9669	0.9071	1.1729	0.8937	0.1987	0.5085	2.5147
3'-AMP	Nucleotide	Purine Metabolism, Adenine containing	35142	LC/MS Neg	100001449	0.9060	0.6847	0.7292	0.4405	0.9764	0.8115	0.8277	0.6700	0.6957	18.6391	1.0000	1.0324	2.2666	2.4015	2.7526	2.3770	1.1692	1.0000	1.7172	1.1012	1.8558	1.1917	1.4430	1.5272	0.9795	0.9044	0.9579	0.8856	0.7992	1.7008
ADP	Nucleotide	Purine Metabolism, Adenine containing	3108	LC/MS Neg	208	0.8777	0.8814	1.1274	0.5786	1.0628	1.0100	1.1683	0.9945	0.7833	0.3108	0.8671	0.8139	1.3572	0.8888	0.9209	1.3612	1.3661	1.3593	0.9729	1.2549	1.0055	0.6911	0.9769	1.3335	1.3563	0.7143	0.5708	1.1491	1.2637	0.9449
adenosine 5'-diphosphoribose (ADP-ribose)	factors and Vitami	Nicotinate and Nicotinamide Metabolism	558	LC/MS Pos Early	215	0.1754	0.1754	0.1754	0.1754	0.1754	0.1754	0.8837	1.0000	0.8566	0.1754	1.1327	1.2298	0.5433	0.6738	1.3466	0.7854	0.6510	0.5746	0.7233	0.1754	0.1754	0.1754	0.5229	0.1930	1.7813	1.6960	0.1754	1.6980	0.8621	0.7144
AMP	Nucleotide	Purine Metabolism, Adenine containing	32342	LC/MS Neg	209	0.6547	0.6024	0.5774	0.4573	0.5868	0.6962	1.3060	1.1459	0.8921	1.1434	1.0637	1.2633	1.0815	0.8325	0.8272	0.9818	0.9560	0.9802	1.1654	1.0828	1.0539	0.8235	1.1163	1.2991	1.2189	1.1018	1.3168	1.5676	1.0182	1.0713
adenosine-5'-diphosphoglucose	Carbohydrate	Nucleotide Sugar	35652	LC/MS Neg	100001644	0.2395	0.6036	0.9454	0.2395	0.2395	0.2395	0.2395	0.2395	0.2395	1.7115	0.2395	0.2395	1.0359	0.8059	1.8906	0.9641	0.5439	0.8277	2.3274	0.2395	2.4121	2.3660	1.5003	0.5268	0.2395	0.4044	0.5979	0.2395	0.2395	0.8538
adipate	Lipid	Fatty Acid, Dicarboxylate	21134	LC/MS Polar	100000863	1.6322	2.4402	3.3089	2.9071	2.3643	1.8461	0.6267	0.8208	0.7610	0.6769	1.0047	0.5623	1.8493	1.1890	1.1761	1.3789	1.3603	1.2440	0.9953	1.4399	1.1064	0.9876	1.1507	1.4241	0.6042	0.8966	1.1607	1.0534	0.7867	1.4634
adipoylcarnitine (C6-DC)	Lipid	Fatty Acid Metabolism(Acyl Carnitine)	52988	LC/MS Pos Early	100006614	0.0349	0.0639	0.0878	0.1002	0.0789	0.0563	0.2788	0.4960	0.5374	0.3932	0.7591	0.7233	2.7819	2.3456	2.1464	1.9822	2.8177	2.4839	1.9094	2.4689	1.1277	1.2347	1.5169	2.5145	1.0619	1.3983	1.7154	1.3382	1.1241	2.8050
adrenate (22:4n6)	Lipid	Polyunsaturated Fatty Acid (n3 and n6)	32980	LC/MS Neg	100001193	0.7789	1.6807	1.7543	1.5199	2.1477	1.9985	2.0818	3.2026	0.7425	8.2642	1.2166	0.8129	0.6548	0.5543	0.8400	1.0187	0.6785	0.8316	0.5809	0.5004	0.9604	0.4992	0.6069	0.7355	1.0408	0.9813	1.1001	1.7303	2.0312	0.5808
alanine	Amino Acid	Alanine and Aspartate Metabolism	1126	LC/MS Pos Early	811	0.7200	1.0265	0.8523	0.7527	0.6768	0.7159	0.8946	1.1714	0.9776	0.7303	1.0224	1.0526	0.8397	0.7462	0.7262	1.0629	1.1046	0.9297	0.8548	0.8651	0.8922	0.8728	0.9523	1.0974	1.0692	0.9382	0.9745	1.1544	1.0888	0.7877
allantoic acid	Nucleotide	ne Metabolism, (Hypo)Xanthine/Inosine contai	33434	LC/MS Polar	218	1.2400	0.5603	1.0542	0.5182	0.5013	1.0000	0.5013	1.7894	0.5013	0.5013	0.5013	0.5013	1.0912	0.5013	1.3120	0.7038	0.5013	0.6062	0.5013	1.4845	0.5013	0.5013	0.7626	0.5013	2.0180	0.5013	0.5013	0.5013	0.5013	0.9032

allantoin	Nucleotide	ne Metabolism, (Hypo)Xanthine/Inosine contai	1107	LC/MS Polar	1002	[illegible]
alpha-hydroxyisocaproate	Amino Acid	Leucine, Isoleucine and Valine Metabolism	22132	LC/MS Neg	100000706	[illegible]
alpha-hydroxyisovalerate	Amino Acid	Leucine, Isoleucine and Valine Metabolism	46537	LC/MS Polar	100001300	[illegible]
alpha-ketoglutaramate*	Amino Acid	Glutamate Metabolism	62101	LC/MS Polar	100002397	[illegible]
alpha-ketoglutarate	Energy	TCA Cycle	528	LC/MS Polar	93	[illegible]
alpha-muricholate	Lipid	Primary Bile Acid Metabolism	34092	LC/MS Neg	100001280	[illegible]
alpha-tocopherol	ofactors and Vitami	Tocopherol Metabolism	1561	LC/MS Pos Late	1105	[illegible]
anserine	Amino Acid	Histidine Metabolism	15747	LC/MS Pos Early	100000044	[illegible]
anthranilate	Amino Acid	Tryptophan Metabolism	4970	LC/MS Pos Early	1258	[illegible]
arabitol/xylitol	Carbohydrate	Pentose Metabolism	48885	LC/MS Polar	100006430	[illegible]
arabonate/xylonate	Carbohydrate	Pentose Metabolism	48255	LC/MS Polar	100006115	[illegible]
arachidate (20:0)	Lipid	Long Chain Fatty Acid	1118	LC/MS Neg	893	[illegible]
arachidonate (20:4n6)	Lipid	Polyunsaturated Fatty Acid (n3 and n6)	1110	LC/MS Neg	229	[illegible]
arachidonoylcarnitine (C20:4)	Lipid	Fatty Acid Metabolism(Acyl Carnitine)	57518	LC/MS Pos Late	100015837	[illegible]
arachidonoylcholine	Lipid	Fatty Acid Metabolism (Acyl Choline)	53261	LC/MS Pos Late	100009332	[illegible]
arachidoylcarnitine (C20)*	Lipid	Fatty Acid Metabolism(Acyl Carnitine)	57513	LC/MS Pos Late	100015833	[illegible]
argininate*	Amino Acid	Urea cycle; Arginine and Proline Metabolism	57461	LC/MS Pos Early	100002769	[illegible]
arginine	Amino Acid	Urea cycle; Arginine and Proline Metabolism	1638	LC/MS Pos Early	231	[illegible]
argininosuccinate	Amino Acid	Urea cycle; Arginine and Proline Metabolism	15497	LC/MS Pos Early	232	[illegible]
ascorbate (Vitamin C)	ofactors and Vitami	Ascorbate and Aldarate Metabolism	32354	LC/MS Pos Early	233	[illegible]
asparagine	Amino Acid	Alanine and Aspartate Metabolism	512	LC/MS Pos Early	917	[illegible]
aspartate	Amino Acid	Alanine and Aspartate Metabolism	443	LC/MS Pos Early	234	[illegible]
azelate (nonanedioate; C9)	Lipid	Fatty Acid, Dicarboxylate	18362	LC/MS Neg	2029	[illegible]
behenate (22:0)*	Lipid	Long Chain Fatty Acid	12125	LC/MS Neg	314	[illegible]
behenoyl dihydrosphingomyelin (d18:0/22:0)*	Lipid	Dihydrosphingomyelins	57331	LC/MS Pos Late	100009026	[illegible]
behenoyl sphingomyelin (d18:1/22:0)*	Lipid	Sphingomyelins	48492	LC/MS Pos Late	100006294	[illegible]
behenoylcarnitine (C22)*	Lipid	Fatty Acid Metabolism(Acyl Carnitine)	57514	LC/MS Pos Late	100015832	[illegible]
benzoate	Xenobiotics	Benzoate Metabolism	15778	LC/MS Neg	100000008	[illegible]
beta-alanine	Nucleotide	Pyrimidine Metabolism, Uracil containing	55	LC/MS Pos Early	244	[illegible]
beta-guanidinopropanoate	Xenobiotics	Food Component/Plant	35101	LC/MS Pos Early	100001411	[illegible]
beta-hydroxyisovalerate	Amino Acid	Leucine, Isoleucine and Valine Metabolism	12129	LC/MS Polar	1442	[illegible]
betaine	Amino Acid	Glycine, Serine and Threonine Metabolism	3141	LC/MS Pos Early	799	[illegible]
betaine aldehyde	Amino Acid	Glycine, Serine and Threonine Metabolism	15499	LC/MS Pos Early	1219	[illegible]
beta-muricholate	Lipid	Primary Bile Acid Metabolism	31885	LC/MS Neg	100001066	[illegible]
beta-sitosterol	Lipid	Sterol	27414	LC/MS Pos Late	100001033	[illegible]
bilirubin	ofactors and Vitami	Hemoglobin and Porphyrin Metabolism	43807	LC/MS Pos Late	1090	[illegible]
biliverdin	ofactors and Vitami	Hemoglobin and Porphyrin Metabolism	2137	LC/MS Pos Late	250	[illegible]
biopterin	ofactors and Vitami	Tetrahydrobiopterin Metabolism	12358	LC/MS Neg	1548	[illegible]
butyrylglycine (C4)	Lipid	Fatty Acid Metabolism (also BCAA Metabolism	31850	LC/MS Neg	100001151	[illegible]
cadaverine	Amino Acid	Lysine Metabolism	15308	LC/MS Pos Early	100000110	[illegible]
campesterol	Lipid	Sterol	33997	LC/MS Pos Late	100001269	[illegible]
caprate (10:0)	Lipid	Medium Chain Fatty Acid	1642	LC/MS Neg	888	[illegible]
caproate (6:0)	Lipid	Medium Chain Fatty Acid	32489	LC/MS Neg	926	[illegible]
caprylate (8:0)	Lipid	Medium Chain Fatty Acid	32492	LC/MS Neg	932	[illegible]
carboxyethyl-GABA	Amino Acid	Glutamate Metabolism	40007	LC/MS Pos Early	100003260	[illegible]
carnitine	Lipid	Carnitine Metabolism	15500	LC/MS Pos Early	100000007	[illegible]
carnosine	Amino Acid	Histidine Metabolism	1768	LC/MS Pos Early	249	[illegible]
carotene diol (1)	ofactors and Vitami	Vitamin A Metabolism	57635	LC/MS Pos Late	100015966	[illegible]
ceramide (d18:1/14:0, d16:1/16:0)*	Lipid	Ceramides	57432	LC/MS Pos Late	100015735	[illegible]
ceramide (d18:1/17:0, d17:1/18:0)*	Lipid	Ceramides	57434	LC/MS Pos Late	100015737	[illegible]
C-glycosyltryptophan	Amino Acid	Tryptophan Metabolism	48782	LC/MS Pos Early	100006379	[illegible]
chenodeoxycholate	Lipid	Primary Bile Acid Metabolism	1563	LC/MS Neg	1123	[illegible]
cholate	Lipid	Primary Bile Acid Metabolism	22842	LC/MS Neg	136	[illegible]
cholate sulfate	Lipid	Primary Bile Acid Metabolism	52973	LC/MS Neg	100006637	[illegible]
cholesterol	Lipid	Sterol	63	LC/MS Pos Late	266	[illegible]
choline	Lipid	Phospholipid Metabolism	15506	LC/MS Pos Early	1256	[illegible]
phosphocholine	Lipid	Phospholipid Metabolism	34396	LC/MS Pos Early	267	[illegible]
cinnamoylglycine	Xenobiotics	Food Component/Plant	38637	LC/MS Neg	100002253	[illegible]
cis-4-decenoate (10:1n6)*	Lipid	Medium Chain Fatty Acid	62873	LC/MS Neg	100002796	[illegible]
cis-4-decenoylcarnitine (C10:1)	Lipid	Fatty Acid Metabolism(Acyl Carnitine)	38178	LC/MS Pos Late	100002259	[illegible]
citrate	Energy	TCA Cycle	1564	LC/MS Neg	1124	[illegible]
citrulline	Amino Acid	Urea cycle; Arginine and Proline Metabolism	2132	LC/MS Pos Early	391	[illegible]
CoA-glutathione*	Amino Acid	Glutathione Metabolism	62804	LC/MS Neg	269	[illegible]
corticosterone	Lipid	Corticosteroids	5983	LC/MS Neg	272	[illegible]
creatine	Amino Acid	Creatine Metabolism	27718	LC/MS Pos Early	1221	[illegible]
creatinine	Amino Acid	Creatine Metabolism	513	LC/MS Pos Early	275	[illegible]
cystathionine	Amino Acid	ethionine, Cysteine, SAM and Taurine Metaboli	15705	LC/MS Pos Early	310	[illegible]
cysteine	Amino Acid	ethionine, Cysteine, SAM and Taurine Metaboli	1868	LC/MS Pos Early	800	[illegible]
cysteine-glutathione disulfide	Amino Acid	Glutathione Metabolism	35159	LC/MS Pos Early	100001437	[illegible]
cysteinylglycine	Amino Acid	Glutathione Metabolism	35637	LC/MS Pos Early	278	[illegible]
cystine	Amino Acid	ethionine, Cysteine, SAM and Taurine Metaboli	56	LC/MS Pos Early	279	[illegible]
cytidine	Nucleotide	Pyrimidine Metabolism, Cytidine containing	514	LC/MS Pos Early	827	[illegible]
cytidine 2'-monophosphate (2'-CMP)	Nucleotide	Pyrimidine Metabolism, Cytidine containing	57769	LC/MS Polar	100002303	[illegible]
3'-CMP	Nucleotide	Pyrimidine Metabolism, Cytidine containing	2959	LC/MS Polar	105	[illegible]
CDP-choline	Lipid	Phospholipid Metabolism	34418	LC/MS Polar	1220	[illegible]
CMP	Nucleotide	Pyrimidine Metabolism, Cytidine containing	2372	LC/MS Neg	282	[illegible]
CDP-ethanolamine	Lipid	Phospholipid Metabolism	34410	LC/MS Polar	100001407	[illegible]
cytosine	Nucleotide	Pyrimidine Metabolism, Cytidine containing	573	LC/MS Pos Early	881	[illegible]
daidzein	Xenobiotics	Food Component/Plant	32453	LC/MS Neg	100001219	[illegible]
dehydroascorbate	ofactors and Vitami	Ascorbate and Aldarate Metabolism	1659	LC/MS Polar	301	[illegible]
deoxycarnitine	Lipid	Carnitine Metabolism	36747	LC/MS Pos Early	100001662	[illegible]
deoxycholate	Lipid	Secondary Bile Acid Metabolism	1114	LC/MS Neg	302	[illegible]
diacylglycerol (12:0/18:1, 14:0/16:1, 16:0/14:1) [2]*	Lipid	Diacylglycerol	55001	LC/MS Pos Late	100010959	[illegible]
diacylglycerol (14:0/18:1, 16:0/16:1) [1]*	Lipid	Diacylglycerol	54953	LC/MS Pos Late	100010934	[illegible]
diacylglycerol (14:0/18:1, 16:0/16:1) [2]*	Lipid	Diacylglycerol	54954	LC/MS Pos Late	100010935	[illegible]
diacylglycerol (16:1/18:2 [2], 16:0/18:3 [1])*	Lipid	Diacylglycerol	54966	LC/MS Pos Late	100010940	[illegible]
dihomolinoleate (20:2n6)	Lipid	Polyunsaturated Fatty Acid (n3 and n6)	17805	LC/MS Neg	1231	[illegible]
dihomolinolenate (20:3n3 or 3n6)	Lipid	Polyunsaturated Fatty Acid (n3 and n6)	35718	LC/MS Neg	100001739	[illegible]
dihydrobiopterin	ofactors and Vitami	Tetrahydrobiopterin Metabolism	35129	LC/MS Neg	100001506	[illegible]
dihydroorotate	Nucleotide	Pyrimidine Metabolism, Orotate containing	601	LC/MS Polar	923	[illegible]
dihydroxyacetone phosphate (DHAP)	Carbohydrate	colysis, Gluconeogenesis, and Pyruvate Metabo	15522	LC/MS Neg	309	[illegible]
dimethylarginine (ADMA + SDMA)	Amino Acid	Urea cycle; Arginine and Proline Metabolism	36808	LC/MS Pos Early	100001810	[illegible]
dimethylglycine	Amino Acid	Glycine, Serine and Threonine Metabolism	5086	LC/MS Pos Early	806	[illegible]
docosadienoate (22:2n6)	Lipid	Polyunsaturated Fatty Acid (n3 and n6)	32415	LC/MS Neg	100001182	[illegible]
docosahexaenoate (DHA; 22:6n3)	Lipid	Polyunsaturated Fatty Acid (n3 and n6)	44675	LC/MS Neg	100000665	[illegible]
docosahexaenoylcarnitine (C22:6)*	Lipid	Fatty Acid Metabolism(Acyl Carnitine)	57523	LC/MS Pos Late	100015845	[illegible]
docosapentaenoate (DPA; 22:5n3)	Lipid	Polyunsaturated Fatty Acid (n3 and n6)	32504	LC/MS Neg	100001181	[illegible]
docosapentaenoate (n6 DPA; 22:5n6)	Lipid	Polyunsaturated Fatty Acid (n3 and n6)	37478	LC/MS Neg	100001580	[illegible]
dodecadienoate (12:2)*	Lipid	Fatty Acid, Dicarboxylate	62276	LC/MS Neg	100020478	[illegible]
dodecanedioate (C12)	Lipid	Fatty Acid, Dicarboxylate	32388	LC/MS Neg	100001102	[illegible]
dodecenedioate (C12:1-DC)*	Lipid	Fatty Acid, Dicarboxylate	61864	LC/MS Neg	100019972	[illegible]
eicosapentaenoate (EPA; 20:5n3)	Lipid	Polyunsaturated Fatty Acid (n3 and n6)	18467	LC/MS Neg	2050	[illegible]
eicosenoate (20:1n9 or 1n11)	Lipid	Long Chain Fatty Acid	33587	LC/MS Neg	100001335	[illegible]
eicosenoylcarnitine (C20:1)*	Lipid	Fatty Acid Metabolism(Acyl Carnitine)	57519	LC/MS Pos Late	100015838	[illegible]
enterolactone	Xenobiotics	Food Component/Plant	39626	LC/MS Neg	100003070	[illegible]
equol sulfate	Xenobiotics	Food Component/Plant	40478	LC/MS Neg	100003429	[illegible]
ergothioneine	Xenobiotics	Food Component/Plant	37459	LC/MS Pos Early	100002154	[illegible]
erucate (22:1n9)	Lipid	Long Chain Fatty Acid	1552	LC/MS Neg	1087	[illegible]
erucoylcarnitine (C22:1)*	Lipid	Fatty Acid Metabolism(Acyl Carnitine)	57525	LC/MS Pos Late	100015841	[illegible]
erythritol	Xenobiotics	Food Component/Plant	20699	LC/MS Polar	100000846	[illegible]
erythronate*	Carbohydrate	Aminosugar Metabolism	42420	LC/MS Polar	100001320	[illegible]
ethylmalonate	Amino Acid	Leucine, Isoleucine and Valine Metabolism	15765	LC/MS Polar	2054	[illegible]
ferulic acid 4-sulfate	Xenobiotics	Food Component/Plant	47114	LC/MS Neg	100005389	[illegible]
FAD	ofactors and Vitami	Riboflavin Metabolism	2134	LC/MS Neg	327	[illegible]
FMN	ofactors and Vitami	Riboflavin Metabolism	15797	LC/MS Neg	100000251	[illegible]
folate	ofactors and Vitami	Folate Metabolism	1826	LC/MS Neg	328	[illegible]
formiminoglutamate	Amino Acid	Histidine Metabolism	43493	LC/MS Pos Early	100002500	[illegible]
fructose	Carbohydrate	Fructose, Mannose and Galactose Metabolism	577	LC/MS Polar	878	[illegible]
Isobar: hexose diphosphates	Carbohydrate	colysis, Gluconeogenesis, and Pyruvate Metabo	46896	LC/MS Neg	100002180	[illegible]
fumarate	Energy	TCA Cycle	1643	LC/MS Polar	330	[illegible]
galactonate	Carbohydrate	Fructose, Mannose and Galactose Metabolism	27719	LC/MS Polar	100001026	[illegible]
galactose 1-phosphate	Carbohydrate	Fructose, Mannose and Galactose Metabolism	15706	LC/MS Polar	100000300	[illegible]
gamma-carboxyglutamate	Amino Acid	Glutamate Metabolism	38754	LC/MS Pos Early	100002679	[illegible]
gamma-glutamyl-alpha-lysine	Peptide	Gamma-glutamyl Amino Acid	55015	LC/MS Pos Early	100010901	[illegible]
gamma-glutamylcitrulline*	Peptide	Gamma-glutamyl Amino Acid	62104	LC/MS Pos Early	100020241	[illegible]
gamma-glutamyl-epsilon-lysine	Peptide	Gamma-glutamyl Amino Acid	33934	LC/MS Pos Early	100001262	[illegible]
gamma-glutamylglutamate	Peptide	Gamma-glutamyl Amino Acid	36738	LC/MS Pos Early	331	[illegible]
gamma-glutamylglutamine	Peptide	Gamma-glutamyl Amino Acid	2730	LC/MS Pos Early	1140	[illegible]
gamma-glutamylglycine	Peptide	Gamma-glutamyl Amino Acid	33949	LC/MS Pos Early	100001294	[illegible]
gamma-glutamylhistidine	Peptide	Gamma-glutamyl Amino Acid	18245	LC/MS Pos Early	1235	[illegible]
gamma-glutamylisoleucine*	Peptide	Gamma-glutamyl Amino Acid	34456	LC/MS Pos Early	100001485	[illegible]
gamma-glutamylleucine	Peptide	Gamma-glutamyl Amino Acid	18369	LC/MS Pos Early	1268	[illegible]
gamma-glutamylmethionine	Peptide	Gamma-glutamyl Amino Acid	44872	LC/MS Pos Early	100001313	[illegible]
gamma-glutamylphenylalanine	Peptide	Gamma-glutamyl Amino Acid	33422	LC/MS Pos Early	100000491	[illegible]
gamma-glutamylserine	Peptide	Gamma-glutamyl Amino Acid	54914	LC/MS Pos Early	100009231	[illegible]
gamma-glutamylthreonine	Peptide	Gamma-glutamyl Amino Acid	33364	LC/MS Pos Early	100001314	[illegible]
gamma-glutamyltryptophan	Peptide	Gamma-glutamyl Amino Acid	33947	LC/MS Pos Early	100001295	[illegible]
gamma-glutamyltyrosine	Peptide	Gamma-glutamyl Amino Acid	2734	LC/MS Pos Early	1102	[illegible]
gamma-glutamylvaline	Peptide	Gamma-glutamyl Amino Acid	43829	LC/MS Pos Early	100001126	[illegible]
gamma-tocopherol/beta-tocopherol	ofactors and Vitami	Tocopherol Metabolism	52473	LC/MS Pos Late	100008998	[illegible]
genistein	Xenobiotics	Food Component/Plant	32448	LC/MS Neg	100001221	[illegible]
gluconate	Xenobiotics	Food Component/Plant	587	LC/MS Polar	338	[illegible]
glucose	Carbohydrate	colysis, Gluconeogenesis, and Pyruvate Metabo	20488	LC/MS Polar	572	[illegible]
glucose 6-phosphate	Carbohydrate	colysis, Gluconeogenesis, and Pyruvate Metabo	31260	LC/MS Polar	291	[illegible]
glucuronate	Carbohydrate	Aminosugar Metabolism	15443	LC/MS Polar	100000257	[illegible]
glutamate	Amino Acid	Glutamate Metabolism	57	LC/MS Pos Early	561	[illegible]
glutamate, gamma-methyl ester	Amino Acid	Glutamate Metabolism	33487	LC/MS Pos Early	100001103	[illegible]
glutamine	Amino Acid	Glutamate Metabolism	53	LC/MS Pos Early	563	[illegible]
glutarate (C5-DC)	Lipid	Fatty Acid, Dicarboxylate	396	LC/MS Polar	339	[illegible]
glutaroylcarnitine (C5)	Amino Acid	Lysine Metabolism	44664	LC/MS Pos Early	100001593	[illegible]
glutathione, oxidized (GSSG)	Amino Acid	Glutathione Metabolism	27727	LC/MS Pos Early	448	[illegible]
glutathione, reduced (GSH)	Amino Acid	Glutathione Metabolism	2127	LC/MS Pos Early	496	[illegible]
glycerate	Carbohydrate	colysis, Gluconeogenesis, and Pyruvate Metabo	1572	LC/MS Polar	1052	[illegible]
glycerol	Lipid	Glycerolipid Metabolism	15122	LC/MS Neg	1254	[illegible]
glycerol 3-phosphate	Lipid	Glycerolipid Metabolism	43847	LC/MS Pos Early	100000258	[illegible]
glycerophosphoethanolamine	Lipid	Phospholipid Metabolism	37455	LC/MS Polar	100001620	[illegible]
glycerophosphoglycerol	Lipid	Glycerolipid Metabolism	48857	LC/MS Polar	100001619	[illegible]
glycerophosphoinositol*	Lipid	Phospholipid Metabolism	52307	LC/MS Pos Early	100001621	[illegible]
glycerophosphorylcholine (GPC)	Lipid	Phospholipid Metabolism	15990	LC/MS Pos Early	100000269	[illegible]
glycerophosphoserine*	Lipid	Phospholipid Metabolism	57404	LC/MS Pos Early	100015666	[illegible]
glycine	Amino Acid	Glycine, Serine and Threonine Metabolism	58	LC/MS Pos Early	340	[illegible]
glycine conjugate of C10H14O2 (1)*	ly Characterized Mo	Partially Characterized Molecules	62146	LC/MS Neg	100020254	[illegible]
glyco-beta-muricholate	Lipid	Primary Bile Acid Metabolism	62066	LC/MS Neg	100020214	[illegible]
glycocholate	Lipid	Primary Bile Acid Metabolism	18476	LC/MS Neg	342	[illegible]
glycosyl ceramide (d18:1/20:0, d16:1/22:0)*	Lipid	Hexosylceramides (HCER)	57595	LC/MS Pos Late	100015882	[illegible]
glycosyl ceramide (d18:2/24:1, d18:1/24:2)*	Lipid	Hexosylceramides (HCER)	57453	LC/MS Pos Late	100015745	[illegible]
glycosyl-N-nervonoyl-sphingosine (d18:1/24:1)*	Lipid	Hexosylceramides (HCER)	57369	LC/MS Pos Late	100001878	[illegible]
glycosyl-N-palmitoyl-sphingosine (d18:1/16:0)	Lipid	Hexosylceramides (HCER)	53013	LC/MS Pos Late	100009272	[illegible]
glycosyl-N-stearoyl-sphingosine (d18:1/18:0)	Lipid	Hexosylceramides (HCER)	52234	LC/MS Pos Late	100001882	[illegible]
glycylleucine	Peptide	Dipeptide	34398	LC/MS Pos Early	100001258	[illegible]
glycylvaline	Peptide	Dipeptide	18357	LC/MS Pos Early	100000487	[illegible]
guanidinoacetate	Amino Acid	Creatine Metabolism	43802	LC/MS Pos Early	344	[illegible]
guanidinosuccinate	Amino Acid	Guanidino and Acetamido Metabolism	32446	LC/MS Pos Early	100001212	[illegible]
guanine	Nucleotide	Purine Metabolism, Guanine containing	32352	LC/MS Pos Early	172	[illegible]
guanosine	Nucleotide	Purine Metabolism, Guanine containing	1573	LC/MS Neg	1099	[illegible]
guanosine 2'-monophosphate (2'-GMP)*	Nucleotide	Purine Metabolism, Guanine containing	52442	LC/MS Neg	100002733	[illegible]
guanosine 3'-monophosphate (3'-GMP)	Nucleotide	Purine Metabolism, Guanine containing	39786	LC/MS Neg	100002704	[illegible]
guanosine 5'-diphospho-fucose	Carbohydrate	Nucleotide Sugar	15903	LC/MS Neg	335	[illegible]
gulonate*	ofactors and Vitami	Ascorbate and Aldarate Metabolism	46957	LC/MS Polar	100001586	[illegible]
heme	ofactors and Vitami	Hemoglobin and Porphyrin Metabolism	41754	LC/MS Neg	100001386	[illegible]
heneicosapentaenoate (21:5n3)	Lipid	Polyunsaturated Fatty Acid (n3 and n6)	57658	LC/MS Neg	100015797	[illegible]
HEPES	Xenobiotics	Chemical	21248	LC/MS Pos Early	100000843	[illegible]
heptadecasphingosine (d17:1)	Lipid	Sphingosines	57427	LC/MS Pos Late	100015724	[illegible]
heptadecatrienoate (17:3)*	Lipid	Long Chain Fatty Acid	62383	LC/MS Neg	100020715	[illegible]
heptanoyl glycine	Lipid	Fatty Acid Metabolism(Acyl Glycine)	42629	LC/MS Neg	100004195	[illegible]
heptenedioate (C7:1-DC)*	Lipid	Fatty Acid, Dicarboxylate	61866	LC/MS Polar	100019981	[illegible]
hexadecadienoate (16:2n6)	Lipid	Polyunsaturated Fatty Acid (n3 and n6)	57652	LC/MS Neg	100009394	[illegible]
hexadecanedioate (C16)	Lipid	Fatty Acid, Dicarboxylate	35678	LC/MS Neg	100001614	[illegible]
hexadecasphingosine (d16:1)*	Lipid	Sphingosines	57428	LC/MS Pos Late	100015723	[illegible]
hexadecatrienoate (16:3n3)	Lipid	Polyunsaturated Fatty Acid (n3 and n6)	57651	LC/MS Neg	100009393	[illegible]
hexadecenedioate (C16:1-DC)*	Lipid	Fatty Acid, Dicarboxylate	61862	LC/MS Neg	100019975	[illegible]
hexanoylglycine (C6)	Lipid	Fatty Acid Metabolism(Acyl Glycine)	35436	LC/MS Neg	100001527	[illegible]
hippurate	Xenobiotics	Benzoate Metabolism	15753	LC/MS Neg	100000014	[illegible]
histidine	Amino Acid	Histidine Metabolism	59	LC/MS Neg	355	[illegible]
histidine betaine (hercynine)*	Xenobiotics	Food Component/Plant	62278	LC/MS Pos Early	100004059	[illegible]
histidylalanine	Peptide	Dipeptide	42027	LC/MS Pos Early	100003542	[illegible]
homocitrulline	Amino Acid	Urea cycle; Arginine and Proline Metabolism	22138	LC/MS Polar	100000963	[illegible]
homostachydrine*	Xenobiotics	Food Component/Plant	33009	LC/MS Pos Early	100001550	[illegible]
hydroxy-N6,N6,N6-trimethyllysine*	Amino Acid	Lysine Metabolism	62959	LC/MS Pos Early	100021123	[illegible]
hydroxypalmitoyl sphingomyelin (d18:1/16:0(OH))	Lipid	Sphingomyelins	62851	LC/MS Pos Late	100021000	[illegible]
hyocholate	Lipid	Secondary Bile Acid Metabolism	34093	LC/MS Neg	100001279	[illegible]
hyodeoxycholate	Lipid	Secondary Bile Acid Metabolism	27531	LC/MS Neg	100000777	[illegible]
hypotaurine	Amino Acid	ethionine, Cysteine, SAM and Taurine Metaboli	590	LC/MS Polar	358	[illegible]
hypoxanthine	Nucleotide	ne Metabolism, (Hypo)Xanthine/Inosine contai	3127	LC/MS Polar	171	[illegible]
imidazole lactate	Amino Acid	Histidine Metabolism	15716	LC/MS Pos Early	100000263	[illegible]
imidazole propionate	Amino Acid	Histidine Metabolism	40730	LC/MS Pos Early	100003434	[illegible]
indoleacetate	Amino Acid	Tryptophan Metabolism	27513	LC/MS Neg	100001034	[illegible]
indoleacetylglycine	Amino Acid	Tryptophan Metabolism	54730	LC/MS Neg	100006730	[illegible]
indolelactate	Amino Acid	Tryptophan Metabolism	18349	LC/MS Neg	100000463	[illegible]
inosine	Nucleotide	ne Metabolism, (Hypo)Xanthine/Inosine contai	1123	LC/MS Neg	361	[illegible]
inositol 1-phosphate (I1P)	Lipid	Inositol Metabolism	43849	LC/MS Polar	370	[illegible]
isobutyrylcarnitine (C4)	Amino Acid	Leucine, Isoleucine and Valine Metabolism	33441	LC/MS Pos Early	100001055	[illegible]
isocaproylglycine	Lipid	Fatty Acid Metabolism(Acyl Glycine)	61789	LC/MS Neg	100019816	[illegible]
isocitrate	Energy	TCA Cycle	12110	LC/MS Polar	1206	[illegible]
isocitric lactone	Energy	TCA Cycle	54724	LC/MS Polar	100009239	[illegible]
isoleucine	Amino Acid	Leucine, Isoleucine and Valine Metabolism	1125	LC/MS Pos Early	376	[illegible]
isoleucylglycine	Peptide	Dipeptide	40008	LC/MS Neg	100003169	[illegible]
isovalerate (C5)	Amino Acid	Leucine, Isoleucine and Valine Metabolism	44656	LC/MS Neg	100000708	[illegible]
isovalerylcarnitine (C5)	Amino Acid	Leucine, Isoleucine and Valine Metabolism	34407	LC/MS Pos Early	100001393	[illegible]
isovalerylglycine	Amino Acid	Leucine, Isoleucine and Valine Metabolism	35107	LC/MS Neg	100001452	[illegible]
kynurenate	Amino Acid	Tryptophan Metabolism	1417	LC/MS Pos Early	98	[illegible]
kynurenine	Amino Acid	Tryptophan Metabolism	15140	LC/MS Pos Early	100000265	[illegible]
lactate	Carbohydrate	colysis, Gluconeogenesis, and Pyruvate Metabo	527	LC/MS Neg	482	[illegible]
lactosyl-N-behenoyl-sphingosine (d18:1/22:0)*	Lipid	Lactosylceramides (LCER)	57422	LC/MS Pos Late	100015623	[illegible]
lactosyl-N-nervonoyl-sphingosine (d18:1/24:1)*	Lipid	Lactosylceramides (LCER)	57370	LC/MS Pos Late	100015620	[illegible]
lactosyl-N-palmitoyl-sphingosine (d18:1/16:0)	Lipid	Lactosylceramides (LCER)	53010	LC/MS Pos Late	100009030	[illegible]
laurate (12:0)	Lipid	Medium Chain Fatty Acid	1645	LC/MS Neg	181	[illegible]
laurylcarnitine (C12)	Lipid	Fatty Acid Metabolism(Acyl Carnitine)	34534	LC/MS Pos Late	100001392	[illegible]
leucine	Amino Acid	Leucine, Isoleucine and Valine Metabolism	60	LC/MS Pos Early	397	[illegible]
leucylalanine	Peptide	Dipeptide	40010	LC/MS Pos Early	100003179	[illegible]
leucylglycine	Peptide	Dipeptide	40045	LC/MS Pos Early	100003185	[illegible]
lignoceroyl sphingomyelin (d18:1/24:0)	Lipid	Sphingomyelins	57330	LC/MS Pos Late	100006298	[illegible]
lignoceroylcarnitine (C24)*	Lipid	Fatty Acid Metabolism(Acyl Carnitine)	57515	LC/MS Pos Late	100015834	[illegible]
linoleate (18:2n6)	Lipid	Polyunsaturated Fatty Acid (n3 and n6)	1105	LC/MS Neg	180	[illegible]
linolenate (18:3n3 or 3n6)	Lipid	Polyunsaturated Fatty Acid (n3 and n6)	34035	LC/MS Neg	100001337	[illegible]
linolenoyl-linolenoyl-glycerol (18:3/18:3) [2]*	Lipid	Diacylglycerol	54952	LC/MS Pos Late	100010933	[illegible]
linoleoyl ethanolamide	Lipid	Endocannabinoid	52608	LC/MS Neg	100006726	[illegible]
linoleoyl-arachidonoyl-glycerol (18:2/20:4) [1]*	Lipid	Diacylglycerol	54955	LC/MS Pos Late	100010922	[illegible]
linoleoyl-arachidonoyl-glycerol (18:2/20:4) [2]*	Lipid	Diacylglycerol	54956	LC/MS Pos Late	100010923	[illegible]
linoleoylcarnitine (C18:2)*	Lipid	Fatty Acid Metabolism(Acyl Carnitine)	46223	LC/MS Pos Late	100003151	[illegible]
linoleoylcholine*	Lipid	Fatty Acid Metabolism (Acyl Choline)	57463	LC/MS Pos Late	100015760	[illegible]
linoleoyl-docosahexaenoyl-glycerol (18:2/22:6) [1]*	Lipid	Diacylglycerol	54949	LC/MS Pos Late	100010928	[illegible]
linoleoyl-docosahexaenoyl-glycerol (18:2/22:6) [2]*	Lipid	Diacylglycerol	54950	LC/MS Pos Late	100010929	[illegible]
linoleoyl-linolenoyl-glycerol (18:2/18:3) [1]*	Lipid	Diacylglycerol	54963	LC/MS Pos Late	100010926	[illegible]
linoleoyl-linolenoyl-glycerol (18:2/18:3) [2]*	Lipid	Diacylglycerol	54964	LC/MS Pos Late	100010927	[illegible]
linoleoyl-linoleoyl-glycerol (18:2/18:2) [1]*	Lipid	Diacylglycerol	54968	LC/MS Pos Late	100010941	[illegible]
linoleoyl-linoleoyl-glycerol (18:2/18:2) [2]*	Lipid	Diacylglycerol	54969	LC/MS Pos Late	100010942	[illegible]
L-urobilin	ofactors and Vitami	Hemoglobin and Porphyrin Metabolism	40173	LC/MS Pos Late	100002568	[illegible]
lysine	Amino Acid	Lysine Metabolism	1301	LC/MS Pos Early	407	[illegible]
malate	Energy	TCA Cycle	1303	LC/MS Polar	409	[illegible]
malonate	Lipid	Fatty Acid Synthesis	15872	LC/MS Polar	818	[illegible]
malonylcarnitine	Lipid	Fatty Acid Synthesis	37059	LC/MS Pos Early	100001526	[illegible]
maltopentaose	Carbohydrate	Glycogen Metabolism	35163	LC/MS Neg	100001447	[illegible]

maltose	Carbohydrate	Glycogen Metabolism	15586	LC/MS Polar	913	1.0703	1.2361	9.5177	7.3232	7.8896	6.3816	0.7375	2.3156	1.0039	0.1713	1.8073	[illegible]
maltotetraose	Carbohydrate	Glycogen Metabolism	15910	LC/MS Neg	100000275	0.9022	0.9209	35.4307	27.7947	92.3613	6.9855	1.5026	1.7094	1.2915	1.7183	1.5269	[illegible]
maltotriose	Carbohydrate	Glycogen Metabolism	44688	LC/MS Polar	100000276	0.0257	0.3858	50.3340	42.8715	72.7162	20.1996	0.0257	1.3698	0.1523	0.0257	0.9576	[illegible]
mannitol/sorbitol	Carbohydrate	Fructose, Mannose and Galactose Metabolism	46142	LC/MS Polar	100001740	0.5747	0.7721	2.1996	0.6244	2.0301	1.1298	1.1541	2.6341	1.3309	0.9773	1.5883	[illegible]
mannonate*	Xenobiotics	Food Component/Plant	62864	LC/MS Polar	100003695	0.9425	0.6637	0.6818	0.4890	0.5143	0.6424	0.0652	1.5707	0.8842	1.0223	0.9107	[illegible]
mannose	Carbohydrate	Fructose, Mannose and Galactose Metabolism	48153	LC/MS Polar	803	1.0516	1.1913	2.1562	2.0549	2.1018	1.6560	0.9996	1.3622	0.9879	0.5425	1.0800	[illegible]
mannose 6-phosphate	Carbohydrate	Fructose, Mannose and Galactose Metabolism	1469	LC/MS Polar	294	1.7282	1.3112	1.9859	2.4135	2.2091	1.5556	0.9643	0.8502	0.6734	8.9750	0.7752	[illegible]
margarate (17:0)	Lipid	Long Chain Fatty Acid	1121	LC/MS Neg	891	1.0551	1.4766	1.0931	1.1074	1.3046	1.3248	1.1127	1.6129	0.8218	2.6984	1.3457	[illegible]
margaroylcarnitine (C17)*	Lipid	Fatty Acid Metabolism(Acyl Carnitine)	57512	LC/MS Pos Late	100004054	0.8206	1.2234	0.7465	0.8916	0.6890	0.7955	0.4616	0.4092	1.2876	0.4371	0.6398	[illegible]
methionine	Amino Acid	[illegible]thionine, Cysteine, SAM and Taurine Metaboli[illegible]	1302	LC/MS Pos Early	415	0.7326	1.0029	0.8005	0.6959	0.8294	0.8138	0.8875	1.0536	1.0356	0.7342	1.0565	[illegible]
methionine sulfoxide	Amino Acid	[illegible]thionine, Cysteine, SAM and Taurine Metaboli[illegible]	18374	LC/MS Pos Early	100000039	0.8210	0.9163	0.6986	0.7230	0.7031	0.5469	0.8861	1.0059	0.9747	0.7398	0.9269	[illegible]
methyl glucopyranoside (alpha + beta)	Xenobiotics	Food Component/Plant	46144	LC/MS Neg	100005864	0.4948	0.5177	1.1412	0.5947	0.9400	1.0923	0.7128	0.9792	0.3588	0.9542	1.0964	[illegible]
methylmalonate (MMA)	Lipid	[illegible]atty Acid Metabolism (also BCAA Metabolism	1496	LC/MS Polar	418	0.6769	1.0100	0.9712	1.2146	1.1768	1.0518	0.8904	1.2158	0.7721	0.7301	1.2073	[illegible]
methylphosphate	Nucleotide	Purine and Pyrimidine Metabolism	37070	LC/MS Neg	100001805	0.5495	0.8184	0.6947	0.6758	0.7365	2.7042	1.1814	1.3583	1.1376	1.5244	1.2113	[illegible]
methylsuccinate	Amino Acid	Leucine, Isoleucine and Valine Metabolism	15745	LC/MS Polar	2051	1.4574	1.4516	1.0763	2.1203	1.5727	1.6887	0.8529	1.1125	1.1598	1.1020	0.9601	[illegible]
myo-inositol	Lipid	Inositol Metabolism	1124	LC/MS Polar	363	0.8084	1.0603	0.8008	0.9191	0.7652	0.9487	0.9349	0.8590	0.9288	1.0668	0.8762	[illegible]
myristate (14:0)	Lipid	Long Chain Fatty Acid	1365	LC/MS Neg	519	1.1087	1.6966	0.9819	0.7566	0.9694	1.2224	0.8739	1.3991	0.5885	2.5697	1.2107	[illegible]
myristoleate (14:1n5)	Lipid	Long Chain Fatty Acid	32418	LC/MS Neg	100001198	1.2252	1.6722	0.9343	0.6316	0.7408	1.0603	0.6712	0.9754	0.6280	3.6010	1.0343	[illegible]
myristoleoylcarnitine (C14:1)*	Lipid	Fatty Acid Metabolism(Acyl Carnitine)	48182	LC/MS Pos Late	100006051	0.6257	1.1822	0.6257	0.8280	0.6257	0.9781	0.8393	0.6257	0.6257	1.7145	0.6257	[illegible]
myristoylcholine*	Lipid	Fatty Acid Metabolism (Acyl Choline)	61506	LC/MS Pos Late	100019770	2.1610	3.9622	3.7731	2.3561	3.5044	4.2915	1.0169	0.5676	0.9765	0.6126	0.5676	[illegible]
myristoyl-linoleoyl-glycerol (14:0/18:2) [1]*	Lipid	Diacylglycerol	57407	LC/MS Pos Late	100015683	2.6066	3.1501	2.1884	1.4277	1.4371	2.5034	0.7653	0.7461	0.8744	0.5679	1.0000	[illegible]
myristoyl-linoleoyl-glycerol (14:0/18:2) [2]*	Lipid	Diacylglycerol	57408	LC/MS Pos Late	100015684	1.5177	2.7292	1.5912	1.0615	1.2519	1.5968	0.7344	1.3997	0.7247	0.2962	1.5404	[illegible]
N('1)-acetylspermidine	Amino Acid	Polyamine Metabolism	57689	LC/MS Pos Early	187	2.5195	0.9225	2.8012	28.6407	27.2831	5.0009	0.1988	0.2993	0.1554	0.4239	0.2238	[illegible]
N-(2-hydroxypalmitoyl)-sphingosine (d18:1/16:0(2OH))	Lipid	Ceramides	57697	LC/MS Pos Late	100015642	0.3395	1.5095	1.1628	2.1836	1.6151	2.1855	0.7386	0.5529	1.0575	0.3395	0.6137	[illegible]
N,N,N-trimethyl-5-aminovalerate	Amino Acid	Lysine Metabolism	57687	LC/MS Pos Early	100015962	0.5044	0.5786	0.3913	0.3977	0.2663	0.2567	1.0629	1.3269	1.2512	0.7220	1.5649	[illegible]
N,N,N-trimethyl-alanylproline betaine (TMAP)	Amino Acid	Urea cycle; Arginine and Proline Metabolism	62947	LC/MS Pos Early	100020217	1.2275	2.2466	1.7591	2.6975	2.0507	2.2745	1.0444	1.0199	0.7359	0.3074	0.4216	[illegible]
N1,N12-diacetylspermine	Amino Acid	Polyamine Metabolism	52987	LC/MS Pos Early	100002132	0.0219	0.0219	0.0219	1.9155	5.1589	0.0845	0.0219	0.0219	0.0219	0.0219	0.0219	[illegible]
N1-Methyl-2-pyridone-5-carboxamide	[illegible]ofactors and Vitami[illegible]	Nicotinate and Nicotinamide Metabolism	40469	LC/MS Neg	100001468	1.0643	1.3869	1.1119	1.5483	1.4181	1.0687	0.9968	1.0032	0.7567	0.7776	1.2896	[illegible]
1-methyladenosine	Nucleotide	Purine Metabolism, Adenine containing	15650	LC/MS Pos Early	1242	1.0710	1.4261	1.1425	1.7317	1.4524	1.2675	1.1049	1.5244	1.4426	0.9983	0.9098	[illegible]

[illegible]

stearoyl-docosahexaenoyl-glycerol (18:0/22:6) [2]*	Lipid	Diacylglycerol	57368	LC/MS Pos Late	100015617	1.0295	1.1479	0.6995	0.8946	0.7362	1.0097	0.8224	0.7574	1.2447	0.2468	0.7603	1.0171	0.5803	0.4535	0.5335	0.7897	0.6929	0.6529	0.6320	1.2445	0.3305	0.4626	0.5822	0.6077	1.2262	1.4640	0.9903	1.2343	1.2643	0.3750
stearoyl-linoleoyl-glycerol (18:0/18:2) [2]*	Lipid	Diacylglycerol	54947	LC/MS Pos Late	100010921	1.9409	2.8085	1.8934	1.8464	1.5932	2.0847	0.7586	1.1083	1.2093	0.6515	1.0380	0.8464	0.6859	0.7324	0.7230	1.0048	0.8891	0.9888	0.9289	1.0547	0.7162	1.0524	0.9952	1.1495	0.8818	0.9702	0.8826	0.9417	0.8267	0.5634
suberate (C8-DC)	Lipid	Fatty Acid, Dicarboxylate	15730	LC/MS Polar	100000016	0.9687	1.0313	1.8649	3.2285	2.2205	1.3783	0.4325	0.6711	0.7318	0.7432	0.8455	0.5157	4.4884	2.9664	3.3961	3.4090	3.1919	3.3647	2.7746	4.2505	2.4228	2.5333	2.9882	5.8347	0.6822	1.1091	1.1366	1.0671	0.7614	2.4486
suberoylcarnitine (C8-DC)	Lipid	Fatty Acid Metabolism(Acyl Carnitine)	52990	LC/MS Pos Early	100006627	0.0478	0.2074	0.3891	0.4285	0.2903	0.1273	0.2079	0.6278	0.6301	0.7622	0.8217	0.8636	6.1772	8.0390	4.7783	2.8797	5.4042	4.6285	5.2566	6.8913	5.8063	6.0068	3.7924	12.0935	0.8867	1.4887	1.0259	1.4391	0.8552	3.1625
suberylglycine	Lipid	Fatty Acid Metabolism(Acyl Glycine)	35419	LC/MS Neg	100001528	1.0968	0.6228	0.4119	0.3250	0.2943	0.2916	3.8890	1.7391	2.3653	0.7326	1.1547	0.5402	0.6815	0.2112	0.5614	0.5730	0.4030	0.4724	1.0484	0.5456	1.3082	0.9064	0.8322	1.3242	1.5078	0.9516	2.5932	1.8888	1.3134	0.4733
succinate	Energy	TCA Cycle	1437	LC/MS Polar	252	0.8529	1.4115	0.8149	1.6025	1.3117	1.3044	0.9687	1.4627	1.5868	3.3662	1.6230	1.0843	0.9416	0.6299	1.3150	0.9569	0.7310	0.9843	0.7304	0.8300	1.3018	1.4395	1.7726	1.1986	1.1311	0.9404	1.0691	0.9704	1.0121	0.8278
succinylcarnitine (C4)	Energy	TCA Cycle	37058	LC/MS Pos Early	100001948	0.1068	0.1068	0.1068	0.1990	0.1705	0.1068	0.4245	0.7451	0.6675	0.5724	1.1614	1.0530	0.9458	0.6139	1.1812	0.7283	1.0875	1.4361	0.5236	0.3237	0.2993	0.3118	0.8124	1.3652	1.6832	1.3406	1.9511	1.2551	1.1758	1.6359
sulfate*	Xenobiotics	Chemical	46960	LC/MS Neg	100002528	0.9570	1.0592	1.1993	1.0402	1.0468	0.8992	1.0826	0.9760	0.7711	0.2434	1.0803	0.9696	1.0289	0.8325	0.7109	0.8005	1.0037	0.9123	1.0087	0.9152	0.9022	0.7556	0.8451	0.7655	1.2392	1.1407	0.9597	0.9914	1.0386	0.9437
tartronate (hydroxymalonate)	Xenobiotics	Food Component/Plant	20693	LC/MS Neg	100000840	1.4470	1.2741	1.1719	1.3648	1.1364	1.1360	0.9216	0.7484	1.0284	0.4107	0.7576	0.8643	1.0334	0.7083	0.9491	0.8715	0.9861	0.8714	1.0295	1.1069	1.3119	1.0391	0.8567	0.5011	0.8625	1.0139	0.5907	0.8247	0.9696	0.5616
taurine	Amino Acid	ethionine, Cysteine, SAM and Taurine Metaboli	2125	LC/MS Neg	512	0.9508	1.2551	1.1167	0.7641	0.9658	1.1229	0.8164	1.1440	0.9295	0.6070	0.8749	1.0927	1.1104	1.0003	1.0900	0.9997	1.2056	1.0290	0.9542	1.2659	1.1088	0.8550	0.9420	1.2641	0.8517	0.7869	1.0086	1.0894	0.9464	1.1325
tauro-beta-muricholate	Lipid	Primary Bile Acid Metabolism	33983	LC/MS Neg	100001250	0.6755	0.9217	2.4179	0.5346	0.8339	0.2995	5.5989	5.8269	4.1937	0.6672	0.5919	1.2321	0.9957	0.2354	0.5092	0.9018	0.2367	1.1468	1.4357	0.2008	3.4474	1.3024	1.0043	1.5670	1.0142	1.9312	0.9883	0.5235	0.8140	0.8650
taurochenodeoxycholate	Lipid	Primary Bile Acid Metabolism	18494	LC/MS Neg	1629	0.4693	0.6916	0.8760	0.2378	0.4563	0.7526	3.4101	3.7502	3.3878	0.6286	0.6535	1.3850	0.5442	0.1855	0.5582	0.5019	0.2782	0.5285	0.7334	0.2552	1.2531	0.7580	0.3387	0.7441	1.3196	1.8349	1.4807	1.3805	1.3021	0.3614
taurochenodeoxycholic acid 7-sulfate	Lipid	Secondary Bile Acid Metabolism	52976	LC/MS Neg	100006643	0.3803	1.1166	0.3232	0.3232	0.4647	0.3232	2.5844	2.8159	1.1790	0.6419	0.9128	0.6773	0.3232	0.3232	0.5188	0.3232	0.3232	0.3232	0.3232	0.3232	0.3232	0.3232	0.3232	0.3232	1.1897	0.8042	1.2133	1.9024	1.5745	0.3232
taurocholate	Lipid	Primary Bile Acid Metabolism	18497	LC/MS Neg	1648	0.9167	0.7076	0.8175	0.2405	0.3811	0.5929	2.1841	2.2378	1.7769	0.4481	0.6578	1.3349	1.2238	0.5601	0.9318	1.0805	0.5316	1.6105	1.2664	0.4800	1.6525	0.7224	1.0354	1.2493	1.0910	1.7581	1.0233	0.9175	1.1268	1.0905
taurocyamine	Amino Acid	ethionine, Cysteine, SAM and Taurine Metaboli	35117	LC/MS Neg	100001418	0.8758	1.6389	1.0049	0.6747	0.9033	1.0838	0.7422	0.7681	0.7147	0.7473	0.7309	0.8796	1.1379	0.9610	1.3866	1.5357	1.1441	1.1618	1.1048	2.0884	1.7388	1.0951	1.1595	1.3568	0.7585	1.1301	0.7829	0.9762	0.7383	0.9704
taurodeoxycholate	Lipid	Secondary Bile Acid Metabolism	12261	LC/MS Neg	1668	0.0136	0.1467	0.0136	0.0136	0.0136	0.0615	3.1847	1.5753	1.0520	0.8131	0.7160	1.9333	0.8157	0.3581	0.5474	0.6283	0.5689	1.2293	0.9567	0.1562	1.6770	0.7642	0.8250	0.7631	1.4345	2.9098	1.5606	1.0433	2.1837	0.6778
taurohyocholate*	Lipid	Secondary Bile Acid Metabolism	42603	LC/MS Neg	100004084	0.3214	0.9846	0.3214	0.3214	0.3214	0.3214	2.9385	4.0604	3.4614	0.3538	1.3372	0.3214	0.5934	0.3214	0.4716	0.6809	0.3214	0.8375	0.9714	0.3214	3.4936	1.4879	0.9005	1.3045	0.3884	1.7335	1.0154	0.5333	0.8066	0.6404
taurohyodeoxycholic acid	Lipid	Secondary Bile Acid Metabolism	43588	LC/MS Neg	100003869	0.1745	0.1745	0.1745	0.1745	0.1745	0.1745	0.8825	0.3656	0.4889	0.1745	0.1745	0.1745	0.5869	0.2474	1.0000	0.7151	1.0100	1.8833	1.0894	0.1745	1.0398	0.1745	1.2372	1.0782	2.3662	3.8672	0.6846	1.5493	0.6606	0.3960
taurolithocholate	Lipid	Secondary Bile Acid Metabolism	31889	LC/MS Neg	100001065	0.1524	0.1524	0.1524	0.1524	0.1524	0.1524	3.0088	1.6531	0.9641	0.5226	0.3860	0.6098	0.1524	0.1524	0.1524	0.5823	0.2957	0.1524	0.4983	0.1524	0.3386	0.1524	0.4862	0.1524	1.2151	1.6069	0.9351	0.8853	1.7899	0.1524
tauroursodeoxycholate	Lipid	Secondary Bile Acid Metabolism	39378	LC/MS Neg	100002912	1.8028	0.7143	2.3745	0.7376	1.4217	0.8574	3.3742	3.4616	2.3313	0.6344	0.1699	1.0302	0.9842	0.2419	0.5998	0.8689	0.2219	1.0158	1.0265	0.2767	1.9417	0.6479	0.6215	1.2364	0.8185	1.3812	0.7944	0.5554	0.7947	0.4919
tetradecadienedioate (C14:2-DC)*	Lipid	Fatty Acid, Dicarboxylate	62918	LC/MS Neg	100021100	17.2059	16.7380	7.0771	10.5888	9.7264	18.8075	1.1179	0.9813	0.7623	0.8237	0.7863	0.5226	0.9513	0.7958	0.8472	1.3534	0.7826	0.8937	1.6541	1.5357	3.6401	2.1019	1.0929	1.5956	0.8279	0.8063	1.1074	1.9136	0.7571	0.6691
tetradecadienoate (14:2)*	Lipid	Polyunsaturated Fatty Acid (n3 and n6)	62850	LC/MS Neg	100020343	1.7560	1.9597	1.2252	0.9441	1.0300	1.6104	0.4324	0.5512	0.5749	2.6212	1.0192	0.5093	2.2937	1.8111	2.4039	2.4434	2.5625	2.4841	3.2050	2.5017	3.1834	2.3313	2.7471	3.6220	0.3298	0.3881	0.6914	0.5397	0.3889	1.8589
tetradecanedioate (C14)	Lipid	Fatty Acid, Dicarboxylate	35669	LC/MS Neg	100001613	1.7354	1.9146	3.8990	5.6147	3.8395	1.6238	1.2008	0.7167	0.6206	0.7128	0.5688	0.2955	2.6495	2.0161	2.7326	2.9772	2.0372	2.4645	2.1843	2.3046	2.9020	2.0714	1.9245	3.8469	0.3345	0.3392	0.9145	0.6163	0.5301	3.0649
thiamin (Vitamin B1)	ofactors and Vitami	Thiamine Metabolism	5341	LC/MS Pos Early	873	1.0016	1.0964	0.9028	0.9375	0.9415	1.1892	1.1036	1.0299	0.8702	0.9971	0.9608	0.8424	1.3371	0.8291	1.2634	1.5806	1.1077	1.0996	1.3529	1.5645	0.9239	0.7289	1.1850	1.1326	1.1416	1.0953	1.1958	1.0437	1.2402	1.1478
thiamin diphosphate	ofactors and Vitami	Thiamine Metabolism	35670	LC/MS Neg	522	0.6036	1.1819	1.0000	0.7258	0.5348	0.7177	1.0013	0.5183	0.7473	0.7695	0.5523	0.8532	1.1644	0.2912	1.2230	1.2794	0.6361	0.7654	1.0636	0.9121	0.6053	0.8459	1.4601	0.9786	1.3193	1.4411	0.2912	1.0340	0.9764	1.3115
thiamin monophosphate	ofactors and Vitami	Thiamine Metabolism	15798	LC/MS Pos Early	523	1.0069	0.9954	1.1996	1.3907	1.4547	1.1981	1.0181	0.9335	0.6990	0.5600	0.6881	0.6053	1.2389	1.0823	1.1370	1.4059	1.0103	1.0033	1.2570	2.0789	1.0027	0.9173	1.3093	1.1103	1.0292	0.9350	1.0608	0.9251	1.1019	1.1171
thioproline	Xenobiotics	Chemical	53231	LC/MS Neg	100009232	0.8389	1.8781	1.8403	0.7194	1.5621	4.1780	0.9503	0.7609	0.5886	14.9707	0.7250	1.3634	0.7890	0.4387	0.6801	2.3410	0.7017	1.2930	1.4590	1.0082	0.4387	0.5578	1.3629	1.0116	0.9858	0.8486	0.9963	0.6196	0.8361	1.3510
threonate	ofactors and Vitami	Ascorbate and Aldarate Metabolism	27738	LC/MS Polar	100001022	1.9945	1.4274	1.6223	1.6730	1.6075	2.0282	1.0757	0.9997	0.8682	1.0672	0.7468	1.2370	0.7333	0.6910	0.8372	0.6064	0.7450	0.7875	0.8585	1.0085	2.0334	0.8211	0.7350	0.4226	1.0907	1.1467	0.9304	0.9025	1.1519	0.4959
threonine	Amino Acid	Glycine, Serine and Threonine Metabolism	1284	LC/MS Pos Early	564	0.6968	0.9455	0.7621	0.7647	0.6976	0.6847	0.8767	0.9732	0.9260	0.7280	0.9818	0.8621	1.0072	0.7183	0.9428	1.2064	1.1367	0.9771	0.9930	0.9173	1.2266	1.0388	1.1083	1.0175	1.1324	1.0291	1.1240	1.0729	1.2366	0.7667
thymidine	Nucleotide	Pyrimidine Metabolism, Thymine containing	2183	LC/MS Neg	872	0.9610	1.1260	0.8600	1.3583	1.0070	1.3511	1.3313	1.1978	1.9745	0.9874	1.0058	1.0245	0.7849	0.5079	0.5106	0.7341	0.5365	0.4611	1.1077	2.5900	1.0488	0.8122	0.7104	1.0171	1.1891	0.9864	0.6420	1.0322	0.9942	0.4291
thymine	Nucleotide	Pyrimidine Metabolism, Thymine containing	604	LC/MS Neg	882	1.3403	1.3375	1.0575	1.2993	1.1616	1.2095	0.9549	0.9378	0.6307	0.4950	1.1125	1.1342	1.3215	0.7640	0.8816	0.8595	0.8836	0.7838	0.6180	0.6159	0.6682	0.6265	0.8081	0.4763	1.6449	1.2617	0.9989	1.2887	1.5267	0.7885
tigloylglycine	Amino Acid	Leucine, Isoleucine and Valine Metabolism	1598	LC/MS Pos Early	1161	0.8062	0.8075	0.3284	3.6762	2.8389	0.8490	1.8593	0.8323	1.7261	1.0267	0.5357	0.2859	1.3514	0.2859	0.2859	0.2859	0.2859	0.2859	0.2859	0.4759	0.2859	0.2859	0.5899	0.2859	2.9325	0.8791	1.7578	0.6701	1.4075	0.2859
tiglyl carnitine (C5)	Amino Acid	Leucine, Isoleucine and Valine Metabolism	35428	LC/MS Pos Early	100001597	0.4171	0.7530	0.4405	0.6190	0.5474	0.3921	1.1447	1.4876	1.0624	0.2194	1.4989	1.3230	1.3912	0.6370	0.7128	0.6148	0.6849	0.5881	0.6482	1.1294	0.5334	0.4405	0.6092	0.8252	1.1763	1.1631	1.3807	1.5819	1.3978	0.5237
hydroxyproline	Amino Acid	Urea cycle; Arginine and Proline Metabolism	32306	LC/MS Pos Early	1001	2.2853	3.2025	2.6158	1.7444	1.5945	3.0367	0.8861	0.8895	0.8329	2.6009	1.1572	0.7292	0.9680	0.9441	0.8130	0.8017	0.8118	0.8389	0.8776	1.3595	1.0702	1.1313	1.0118	1.3340	0.9882	1.0898	0.7325	0.8672	0.6598	0.9395
tricarballylate	Energy	TCA Cycle	15729	LC/MS Polar	2053	1.5701	3.3342	1.8850	3.0711	1.2062	12.1280	1.1109	0.6726	0.8369	9.7739	3.6156	1.3074	1.3824	1.6572	1.1107	1.0480	3.6714	0.8262	0.7286	2.0467	0.5629	0.4113	0.9520	0.4299	7.1161	5.5863	0.5871	1.2238	0.9286	1.2789
tricosanoyl sphingomyelin (d18:1/23:0)*	Lipid	Sphingomyelins	52436	LC/MS Pos Late	100008955	0.9128	0.7816	0.7017	0.9264	0.9175	0.9255	1.0351	0.8576	1.4234	0.9034	1.0019	0.9143	0.9981	0.7313	0.9948	1.1033	1.0077	1.0934	0.8763	1.0864	0.7517	0.7687	0.8935	0.9140	1.0368	1.1412	0.9442	0.8764	1.2760	0.9010
trigonelline (N'-methylnicotinate)	ofactors and Vitami	Nicotinate and Nicotinamide Metabolism	32401	LC/MS Pos Early	100001092	5.0923	2.9231	3.9616	2.1559	5.4652	3.8187	0.6772	0.4257	0.7234	0.7092	1.9293	0.9662	2.7935	2.6462	2.2854	2.8794	7.9730	1.9698	5.9513	11.2291	2.3745	1.0338	6.6199	1.4947	2.0859	0.9545	0.4826	0.5249	0.5098	5.2590
trimethylamine N-oxide	Lipid	Phospholipid Metabolism	40406	LC/MS Pos Early	100003397	0.2584	0.9061	1.3593	0.4551	0.8608	0.9875	0.5619	0.4577	0.4839	0.8240	1.8113	1.0524	1.6617	1.3050	1.0000	2.1048	1.6898	2.5893	0.8543	1.3448	1.7754	1.3108	1.0436	1.3323	4.3237	1.4225	0.6398	1.0029	1.3670	2.5019
trizma acetate	Xenobiotics	Chemical	20710	LC/MS Pos Early	100000856	0.6075	4.3155	1.0000	4.3073	181.4721	2.5341	0.8258	3.0529	0.4593	1.1058	0.8796	0.4075	3.3716	3.8244	0.3685	1.2760	0.3645	0.5367	9.8776	88.2442	4.5070	4.4320	0.7147	0.5706	0.8392	0.6675	0.3121	0.2863	0.2209	0.3740
tryptophan	Amino Acid	Tryptophan Metabolism	54	LC/MS Pos Early	565	0.6792	0.9983	0.7571	0.5522	0.6164	0.6812	1.0017	1.0540	0.9038	0.8394	1.0728	1.0176	0.9394	0.7939	0.7738	1.0041	1.0437	0.9658	0.8549	0.8210	0.7463	0.8635	0.9597	0.8831	1.1152	1.0501	0.9501	1.1151	1.2744	0.6840
tyrosine	Amino Acid	Tyrosine Metabolism	1299	LC/MS Pos Early	815	0.4413	0.8647	0.6018	0.4332	0.4352	0.4414	0.8104	0.8892	0.8932	0.8645	0.8815	0.7648	1.0149	0.6542	0.8219	1.2207	1.1257	1.1290	1.0570	0.7573	1.2274	1.0218	1.1176	1.1001	1.1164	0.9349	1.0660	0.9731	1.1389	0.7196
tyrosylglycine	Peptide	Dipeptide	41375	LC/MS Neg	100003598	0.3161	0.5895	0.3161	0.5782	0.4327	0.4726	0.7524	0.9674	0.8852	5.5027	0.3589	0.5552	0.7132	0.3454	0.8039	1.6181	1.1929	1.4398	1.1609	0.3161	2.7253	2.9768	1.5697	2.4061	0.4625	0.8737	1.2874	1.2468	0.9083	1.0326
UDP-galactose	Carbohydrate	Nucleotide Sugar	15860	LC/MS Polar	100000298	0.7018	0.7250	0.1049	0.0836	0.0584	0.6079	0.7760	0.5609	0.7207	1.0071	0.7754	0.3218	1.2834	0.9898	1.2760	1.1447	1.3680	1.1051	1.0797	1.0798	0.8435	0.6309	0.8611	1.1124	0.8502	0.6013	0.9494	1.4102	0.8867	1.3541
UDP-glucose	Carbohydrate	Nucleotide Sugar	32344	LC/MS Polar	1096	0.1583	0.0858	0.0031	0.0045	0.0069	0.0825	0.1010	0.0403	0.1097	2.7458	0.0498	0.0102	2.2165	1.1061	1.8047	0.9585	1.8569	2.4222	2.3737	0.5165	1.7713	1.1976	1.0415	2.6550	0.0713	0.0321	0.1128	0.4382	0.1595	2.9076
UDP-N-acetylglucosamine/galactosamine	Carbohydrate	Nucleotide Sugar	46148	LC/MS Neg	100002957	0.5000	1.3003	1.9450	1.4814	2.8154	2.0692	0.1104	0.3635	0.1523	0.7245	0.1904	0.2982	1.2256	0.9633	2.0897	2.1294	1.6393	2.9939	1.7875	0.4947	3.5010	2.9478	2.1590	3.3050	0.3512	0.4228	0.4647	0.3600	0.2356	0.8525
uracil	Nucleotide	Pyrimidine Metabolism, Uracil containing	605	LC/MS Polar	825	0.9582	1.2933	1.5407	2.1016	1.7562	1.3742	1.3717	1.7404	1.4140	1.1745	1.3655	1.0418	0.8865	0.6324	0.7807	0.8375	0.7634	0.7789	0.6362	0.7905	0.6768	0.7177	0.6840	0.6032	1.1680	0.9187	1.1027	1.3257	1.4320	0.7356
urate	Nucleotide	ne Metabolism, (Hypo)Xanthine/Inosine contai	1604	LC/MS Neg	1134	1.1937	0.8546	0.4861	0.5215	0.5615	0.7109	1.5159	1.2478	1.0925	1.1293	0.8740	0.9872	1.7904	1.2903	1.2236	1.7650	1.3993	1.1501	1.1922	1.7168	0.1246	1.0128	1.0762	1.4463	0.7705	0.8524	0.7518	1.2629	0.7921	1.1862
urea	Amino Acid	Urea cycle; Arginine and Proline Metabolism	1670	LC/MS Pos Early	533	0.7016	1.2281	1.5668	1.9730	1.6155	1.1092	0.9599	0.9445	0.8410	0.6914	0.6247	0.7532	1.0772	0.8830	0.9898	1.0617	1.2367	1.0628	1.2228	1.0489	1.2202	0.8018	1.1297	0.9875	1.2478	0.8021	0.7533	0.8945	0.8398	0.9654
uridine	Nucleotide	Pyrimidine Metabolism, Uracil containing	606	LC/MS Neg	535	1.0335	1.0566	0.4969	0.3638	0.3836	1.0114	0.9094	0.8849	0.6584	0.9886	1.1490	1.4063	0.7739	0.8712	0.8644	0.9250	1.1517	1.0893	0.8987	1.0686	0.7898	0.7672	1.0967	1.0659	1.3500	1.3343	0.9813	1.1324	0.9514	0.8686
uridine 2'-monophosphate (2'-UMP)*	Nucleotide	Pyrimidine Metabolism, Uracil containing	52444	LC/MS Neg	100008975	0.0220	0.0220	0.0220	0.0220	0.0220	0.0220	0.0220	0.0402	0.0220	7.5387	0.0220	0.0220	0.0220	0.0220	0.0220	0.0220	0.0220	0.0220	0.0220	0.0220	0.0220	0.0220	0.0220	0.0220	0.0220	0.0220	0.0220	0.0220	0.0220	0.0220
uridine 3'-monophosphate (3'-UMP)	Nucleotide	Pyrimidine Metabolism, Uracil containing	39764	LC/MS Neg	106	0.2369	0.2369	0.2369	0.2369	0.2369	0.2369	0.2369	0.2369	0.2369	55.5613	0.2369	0.2369	0.9939	0.3980	0.2369	4.0769	0.9877	0.7224	0.2369	0.2369	0.7252	1.0581	1.1624	1.5913	0.2369	0.2369	0.2369	0.2369	0.2369	0.3846
uridine 5'-diphosphate (UDP)	Nucleotide	Pyrimidine Metabolism, Uracil containing	5345	LC/MS Neg	870	0.7544	1.2926	1.0724	1.3456	0.7340	0.6628	0.1851	0.1851	0.1851	0.1851	0.1851	0.2892	1.2886	1.4333	1.2663	1.3596	1.4237	1.2152	0.9512	1.4489	0.4062	0.7464	0.9480	0.4878	0.8764	0.2961	1.2609	1.0488	0.2851	0.1851
UMP	Nucleotide	Pyrimidine Metabolism, Uracil containing	2856	LC/MS Polar	869	0.9845	0.7340	0.6316	1.2372	1.5182	1.4179	1.0432	0.4614	0.5539	0.6421	0.5989	0.7558	1.4408	1.0155	0.8588	1.1886	1.1828	0.9196	1.2674	1.1692	1.3000	0.6505	0.6798	1.3464	0.9486	0.6343	1.0979	2.0091	0.7263	1.3986
uridine-2',3'-cyclic monophosphate	Nucleotide	Pyrimidine Metabolism, Uracil containing	37137	LC/MS Neg	75	0.1027	0.1027	0.1027	0.1027	0.1027	0.1027	0.1027	0.1027	0.1027	1.8309	0.1027	0.1027	0.1027	0.1027	0.1027	0.1027	0.1027	0.1027	0.1027	0.1027	0.1027	0.1027	0.1027	0.1027	0.1027	0.1027	0.1027	0.1027	0.1027	0.1027
ursocholate	Lipid	Secondary Bile Acid Metabolism	57573	LC/MS Neg	100002913	0.0351	0.5458	1.0791	1.3754	0.4798	1.1725	1.3199	1.1605	1.7619	0.2485	0.2316	0.3920	0.8659	0.0351	1.3855	3.6683	0.1819	1.5505	1.7322	0.2303	0.2307	0.3934	3.8878	0.7191	1.0950	1.5884	0.6491	0.8680	0.5797	0.0351
ursodeoxycholate	Lipid	Secondary Bile Acid Metabolism	1605	LC/MS Neg	1135	1.9087	0.9222	1.4262	0.6202	0.8969	1.0235	1.8947	2.2264	1.7536	0.3312	0.2777	1.2006	1.3301	0.2836	1.0100	1.3560	0.3363	0.9730	0.8914	0.9416	0.8998	0.3788	0.6419	0.8989	1.1189	1.6399	0.9900	1.1527	1.2252	0.5838
valerylglycine (C5)	Lipid	Fatty Acid Metabolism(Acyl Glycine)	36768	LC/MS Neg	100001451	1.0848	1.2142	0.5882	0.9495	0.7890	0.6781	2.1802	1.0505	0.6592	0.6455	0.6744	1.6151	1.7604	0.8828	0.3055	0.7396	0.5495	0.6214	0.6623	1.9644	1.1055	0.8373	3.6017	0.6719	2.3612	0.7247	2.3911	0.9483	0.8530	0.9040
valine	Amino Acid	Leucine, Isoleucine and Valine Metabolism	1649	LC/MS Pos Early	566	0.7275	0.9761	0.8408	0.9276	0.8906	0.6635	0.9373	1.1044	1.0410	0.8503	1.0194	0.8668	0.9874	0.6922	0.7748	1.0587	1.1412	0.9941	0.8725	0.8524	1.0168	0.9871	1.0059	0.9062	1.1467	0.9893	1.2331	1.1185	1.2838	0.6600
valylglutamine	Peptide	Dipeptide	42079	LC/MS Pos Early	100003640	0.3748	0.3748	0.3748	0.3748	0.9791	1.0545	0.3748	0.3748	0.8027	4.4401	0.3748	0.3748	0.7817	0.3748	0.5936	1.4053	0.5635	0.8513	1.1764	0.4363	2.4475	1.1693	2.1103	5.1028	0.8926	0.6473	2.9308	0.3748	0.8785	1.0209
valylglycine	Peptide	Dipeptide	40475	LC/MS Neg	100003641	0.1799	0.4982	0.5257	0.4435	0.7187	0.7013	0.7987	1.0474	1.0092	2.8158	0.8851	0.9425	0.8587	0.6018	0.6555	1.4625	1.3046	1.2816	1.1152	0.4927	1.6475	1.1998	1.3302	2.3865	0.6854	0.9637	1.5390	1.1419	0.9744	0.9664
valylleucine	Peptide	Dipeptide	39994	LC/MS Neg	100003210	0.2967	0.6360	0.5498	0.6483	0.6670	0.5604	0.8021	0.8849	0.7280	4.9358	0.6302	0.8460	0.8174	0.7078	0.6189	1.1306	1.4745	1.2529	1.5932	0.7731	2.7470	2.0473	2.0213	1.6660	0.6500	0.9313	1.5188	0.8273	0.7152	1.1023
xanthine	Nucleotide	ne Metabolism, (Hypo)Xanthine/Inosine contai	3147	LC/MS Polar	1004	1.0025	1.0329	0.9442	0.8461	0.9155	0.9738	1.0570	1.0195	0.9444	1.0733	1.0014	1.0314	1.0039	0.9059	0.9401	1.0146	1.0464	0.9548	0.9556	1.0240	0.8657	0.8246	0.9324	0.9777	1.0649	1.0114	1.0154	1.1245	1.0591	0.9339
xanthopterin	ofactors and Vitami	Pterin Metabolism	54728	LC/MS Polar	100006338	0.6490	0.6818	0.8993	0.8127	0.8414	0.4383	1.7174	1.7512	1.4716	0.9460	1.3111	1.3416	1.0604	0.9086	1.0693	1.0079	1.1801	0.9170	1.1077	1.0292	0.0496	0.8881	0.9803	0.7068	1.1392	0.9777	1.1877	1.3903	1.4993	0.9697
xanthosine	Nucleotide	ne Metabolism, (Hypo)Xanthine/Inosine contai	15136	LC/MS Neg	100000299	1.0532	1.1535	1.0875	0.7448	0.6854	0.9564	1.3781	1.1859	1.0057	1.3637	1.2172	1.5704	0.9943	0.7481	0.8131	0.8675	0.9037	0.8024	0.7887	1.1609	0.6442	0.5515	0.9233	0.8511	1.1849	1.2021	1.2683	1.3253	1.3746	0.6888
xanthurenate	Amino Acid	Tryptophan Metabolism	15679	LC/MS Neg	100000015	0.5467	1.8763	0.5799	0.3270	0.5369	0.8508	1.1327	0.9019	0.6513	0.5718	0.3738	0.6635	1.6139	0.8679	1.5271	1.4446	2.2797	2.0617	2.2291	4.2975	0.5269	1.2905	1.2008	1.4779	3.3643	1.2007	0.5158	1.0776	0.9725	1.3436

	f.value	p.value	FDR	Tukey's HSD
cytidine	173.17	7.79E-18	6.40E-15	Cpt2 cff-Atgl cff; PPAR-a-Atgl cff; ff:ff-Cpt2 cff; PPAR-a-Cpt2 cff; ff:ff Cre-ff:ff; PPAR-a-ff:ff; PPAR-a-ff:ff Cre
malonylcarnitine	155.04	2.94E-17	1.21E-14	Cpt2 cff-Atgl cff; ff:ff Cre-Atgl cff; PPAR-a-Atgl cff; ff:ff-Cpt2 cff; ff:ff Cre-ff:ff; PPAR-a-ff:ff; PPAR-a-ff:ff Cre
12-dilinoleoyl-GPE182/182	117.69	7.82E-16	2.14E-13	Cpt2 cff-Atgl cff; ff:ff Cre-Atgl cff; PPAR-a-Atgl cff; ff:ff-Cpt2 cff; ff:ff Cre-Cpt2 cff; PPAR-a-Cpt2 cff; ff:ff Cre-ff:ff; PPAR-a-ff:ff; PPAR-a-ff:ff Cre
2-aminooctanoate	112.21	1.37E-15	2.82E-13	PPAR-a-Atgl cff; PPAR-a-Cpt2 cff; PPAR-a-ff:ff; PPAR-a-ff:ff Cre
5-methylcytidine	89.936	1.83E-14	2.46E-12	Cpt2 cff-Atgl cff; ff:ff Cre-Atgl cff; PPAR-a-Atgl cff; ff:ff-Cpt2 cff; PPAR-a-Cpt2 cff; ff:ff Cre-ff:ff; PPAR-a-ff:ff; PPAR-a-ff:ff Cre
palmitoyl-palmitoyl-glycerol160/1601	89.199	2.02E-14	2.46E-12	PPAR-a-Atgl cff; PPAR-a-Cpt2 cff; PPAR-a-ff:ff; PPAR-a-ff:ff Cre
1-stearoyl-2-linoleoyl-GPI180/182	88.891	2.10E-14	2.46E-12	PPAR-a-Atgl cff; PPAR-a-Cpt2 cff; PPAR-a-ff:ff; PPAR-a-ff:ff Cre
1-palmitoyl-2-linoleoyl-GPI160/182	70.859	2.86E-13	2.94E-11	Cpt2 cff-Atgl cff; ff:ff Cre-Atgl cff; PPAR-a-Atgl cff; ff:ff-Cpt2 cff; ff:ff Cre-ff:ff; PPAR-a-ff:ff
2-deoxycytidine	67.181	5.24E-13	4.79E-11	PPAR-a-Atgl cff; ff:ff-Cpt2 cff; PPAR-a-Cpt2 cff; ff:ff Cre-ff:ff; PPAR-a-ff:ff; PPAR-a-ff:ff Cre
2-methylcitrate/homocitrate	65.617	6.85E-13	5.63E-11	Cpt2 cff-Atgl cff; ff:ff-Atgl cff; ff:ff Cre-Atgl cff; PPAR-a-Atgl cff; ff:ff-Cpt2 cff; PPAR-a-Cpt2 cff; ff:ff Cre-ff:ff; PPAR-a-ff:ff; PPAR-a-ff:ff Cre
sphingomyelind181/221d182/220d161/241	64.832	7.85E-13	5.81E-11	Cpt2 cff-Atgl cff; ff:ff Cre-Atgl cff; PPAR-a-Atgl cff; ff:ff-Cpt2 cff; PPAR-a-Cpt2 cff; ff:ff Cre-ff:ff; PPAR-a-ff:ff; PPAR-a-ff:ff Cre
3-hydroxybutyrateBHBA	64.39	8.48E-13	5.81E-11	Cpt2 cff-Atgl cff; ff:ff Cre-Atgl cff; PPAR-a-Atgl cff; ff:ff-Cpt2 cff; PPAR-a-Cpt2 cff; ff:ff Cre-ff:ff; PPAR-a-ff:ff; PPAR-a-ff:ff Cre
1-palmitoyl-2-docosahexaenoyl-GPE160/226	60.616	1.68E-12	9.39E-11	Cpt2 cff-Atgl cff; ff:ff Cre-Atgl cff; PPAR-a-Atgl cff; ff:ff-Cpt2 cff; PPAR-a-Cpt2 cff; ff:ff Cre-ff:ff; PPAR-a-ff:ff; PPAR-a-ff:ff Cre
N-acetylglycine	60.391	1.75E-12	9.39E-11	Cpt2 cff-Atgl cff; ff:ff Cre-Atgl cff; PPAR-a-Atgl cff; ff:ff-Cpt2 cff; PPAR-a-Cpt2 cff; ff:ff Cre-ff:ff; PPAR-a-ff:ff; PPAR-a-ff:ff Cre
1-stearoyl-2-linoleoyl-GPE180/182	60.226	1.80E-12	9.39E-11	Cpt2 cff-Atgl cff; ff:ff Cre-Atgl cff; PPAR-a-Atgl cff; ff:ff-Cpt2 cff; PPAR-a-Cpt2 cff; ff:ff Cre-ff:ff; PPAR-a-ff:ff; PPAR-a-ff:ff Cre
1-palmitoyl-2-docosahexaenoyl-GPC160/226	60.152	1.83E-12	9.39E-11	Cpt2 cff-Atgl cff; ff:ff Cre-Atgl cff; PPAR-a-Atgl cff; ff:ff-Cpt2 cff; PPAR-a-Cpt2 cff; ff:ff Cre-ff:ff; PPAR-a-ff:ff; PPAR-a-ff:ff Cre
1-stearoyl-2-oleoyl-GPC180/181	59.084	2.24E-12	1.08E-10	Cpt2 cff-Atgl cff; ff:ff Cre-Atgl cff; PPAR-a-Atgl cff; ff:ff-Cpt2 cff; PPAR-a-Cpt2 cff; ff:ff Cre-ff:ff; PPAR-a-ff:ff; PPAR-a-ff:ff Cre
1-stearoyl-2-linoleoyl-GPC180/182	58.646	2.43E-12	1.11E-10	Cpt2 cff-Atgl cff; ff:ff Cre-Atgl cff; PPAR-a-Atgl cff; ff:ff-Cpt2 cff; PPAR-a-Cpt2 cff; ff:ff Cre-ff:ff; PPAR-a-ff:ff; PPAR-a-ff:ff Cre
910-DiHOME	56.75	3.51E-12	1.52E-10	PPAR-a-Atgl cff; PPAR-a-Cpt2 cff; PPAR-a-ff:ff; PPAR-a-ff:ff Cre
1-stearoyl-2-linoleoyl-GPS180/182	56.067	4.02E-12	1.65E-10	PPAR-a-Atgl cff; PPAR-a-Cpt2 cff; PPAR-a-ff:ff; PPAR-a-ff:ff Cre
1-oleoyl-2-linoleoyl-GPE181/182	54.769	5.21E-12	2.04E-10	Cpt2 cff-Atgl cff; ff:ff Cre-Atgl cff; PPAR-a-Atgl cff; ff:ff-Cpt2 cff; ff:ff Cre-Cpt2 cff; PPAR-a-Cpt2 cff; ff:ff Cre-ff:ff; PPAR-a-ff:ff
sphingomyelind181/241d182/240	51.859	9.56E-12	3.57E-10	Cpt2 cff-Atgl cff; ff:ff Cre-Atgl cff; PPAR-a-Atgl cff; ff:ff-Cpt2 cff; ff:ff Cre-ff:ff; PPAR-a-ff:ff
adipoylcarnitineC6-DC	50.452	1.29E-11	4.61E-10	Cpt2 cff-Atgl cff; ff:ff-Atgl cff; ff:ff Cre-Atgl cff; ff:ff Cre-Cpt2 cff; PPAR-a-Cpt2 cff; ff:ff Cre-ff:ff; PPAR-a-ff:ff; PPAR-a-ff:ff Cre
gulonate	50.271	1.35E-11	4.61E-10	PPAR-a-Atgl cff; PPAR-a-Cpt2 cff; PPAR-a-ff:ff; PPAR-a-ff:ff Cre
3-hydroxy-3-methylglutarate	49.292	1.67E-11	5.50E-10	Cpt2 cff-Atgl cff; ff:ff Cre-Atgl cff; ff:ff-Cpt2 cff; PPAR-a-Cpt2 cff; ff:ff Cre-ff:ff; PPAR-a-ff:ff Cre
N-acetylaspartateNAA	44.533	5.07E-11	1.60E-09	Cpt2 cff-Atgl cff; ff:ff Cre-Atgl cff; PPAR-a-Atgl cff; ff:ff-Cpt2 cff; ff:ff Cre-ff:ff; PPAR-a-ff:ff
1-palmitoleoyl-2-linolenoyl-GPC161/183	44.124	5.60E-11	1.71E-09	Cpt2 cff-Atgl cff; ff:ff Cre-Atgl cff; PPAR-a-Atgl cff; ff:ff-Cpt2 cff; ff:ff Cre-ff:ff; PPAR-a-ff:ff; PPAR-a-ff:ff Cre
docosahexaenoylcarnitineC226	43.358	6.77E-11	1.99E-09	Cpt2 cff-Atgl cff; ff:ff Cre-Atgl cff; PPAR-a-Atgl cff; ff:ff-Cpt2 cff; ff:ff Cre-ff:ff; PPAR-a-ff:ff
N-myristoyltaurine	42.723	7.94E-11	2.25E-09	PPAR-a-Atgl cff; PPAR-a-Cpt2 cff; PPAR-a-ff:ff; PPAR-a-ff:ff Cre
erythritol	42.109	9.28E-11	2.54E-09	Cpt2 cff-Atgl cff; ff:ff-Atgl cff; ff:ff Cre-Atgl cff; PPAR-a-Atgl cff; ff:ff-Cpt2 cff; ff:ff Cre-ff:ff; PPAR-a-ff:ff
12-dilinoleoyl-GPC182/182	41.175	1.18E-10	3.06E-09	Cpt2 cff-Atgl cff; PPAR-a-Atgl cff; ff:ff-Cpt2 cff; ff:ff Cre-Cpt2 cff; PPAR-a-Cpt2 cff; PPAR-a-ff:ff; PPAR-a-ff:ff Cre
1-linoleoyl-2-linolenoyl-GPC182/183	41.148	1.19E-10	3.06E-09	ff:ff Cre-Atgl cff; PPAR-a-Atgl cff; ff:ff Cre-Cpt2 cff; PPAR-a-Cpt2 cff; ff:ff Cre-ff:ff; PPAR-a-ff:ff; PPAR-a-ff:ff Cre
1-palmitoyl-2-arachidonoyl-GPC160/204n6	40.514	1.40E-10	3.50E-09	Cpt2 cff-Atgl cff; ff:ff Cre-Atgl cff; PPAR-a-Atgl cff; ff:ff-Cpt2 cff; ff:ff Cre-ff:ff; PPAR-a-ff:ff; PPAR-a-ff:ff Cre
2-hydroxyglutarate	40.378	1.46E-10	3.52E-09	Cpt2 cff-Atgl cff; ff:ff Cre-Atgl cff; PPAR-a-Atgl cff; ff:ff-Cpt2 cff; PPAR-a-Cpt2 cff; ff:ff Cre-ff:ff; PPAR-a-ff:ff; PPAR-a-ff:ff Cre
myristoylcholine	39.919	1.65E-10	3.86E-09	PPAR-a-Atgl cff; PPAR-a-Cpt2 cff; PPAR-a-ff:ff; PPAR-a-ff:ff Cre
sphingomyelind182/210d162/230	39.814	1.69E-10	3.86E-09	Cpt2 cff-Atgl cff; ff:ff Cre-Atgl cff; PPAR-a-Atgl cff; ff:ff-Cpt2 cff; ff:ff Cre-ff:ff; PPAR-a-ff:ff; PPAR-a-ff:ff Cre
3-hydroxybutyroylglycine	39.407	1.89E-10	4.20E-09	Cpt2 cff-Atgl cff; ff:ff Cre-Atgl cff; PPAR-a-Atgl cff; ff:ff-Cpt2 cff; PPAR-a-Cpt2 cff; ff:ff Cre-ff:ff; PPAR-a-ff:ff; PPAR-a-ff:ff Cre
2-AMP	37.063	3.62E-10	7.66E-09	Cpt2 cff-Atgl cff; ff:ff Cre-Atgl cff; ff:ff-Cpt2 cff; ff:ff Cre-Cpt2 cff; PPAR-a-Cpt2 cff; ff:ff Cre-ff:ff; PPAR-a-ff:ff Cre
isovalerateC5	36.904	3.79E-10	7.66E-09	Cpt2 cff-Atgl cff; ff:ff-Atgl cff; ff:ff Cre-Atgl cff; PPAR-a-Atgl cff; PPAR-a-Cpt2 cff; PPAR-a-ff:ff; PPAR-a-ff:ff Cre
tetradecadienedioateC142-DC	36.808	3.89E-10	7.66E-09	PPAR-a-Atgl cff; PPAR-a-Cpt2 cff; PPAR-a-ff:ff; PPAR-a-ff:ff Cre
isocitrate	36.754	3.96E-10	7.66E-09	PPAR-a-Atgl cff; PPAR-a-Cpt2 cff; PPAR-a-ff:ff; PPAR-a-ff:ff Cre
R-3-hydroxybutyrylcarnitine	36.711	4.00E-10	7.66E-09	Cpt2 cff-Atgl cff; ff:ff Cre-Atgl cff; PPAR-a-Atgl cff; ff:ff-Cpt2 cff; ff:ff Cre-ff:ff; PPAR-a-ff:ff
isocitriclactone	36.71	4.01E-10	7.66E-09	PPAR-a-Atgl cff; PPAR-a-Cpt2 cff; PPAR-a-ff:ff; PPAR-a-ff:ff Cre
palmitoyl-oleoyl-glycerol160/1811	36.533	4.22E-10	7.87E-09	ff:ff Cre-Atgl cff; PPAR-a-Atgl cff; PPAR-a-Cpt2 cff; PPAR-a-ff:ff; PPAR-a-ff:ff Cre
N-palmitoyl-sphingadienined182/160	35.94	5.01E-10	9.15E-09	Cpt2 cff-Atgl cff; PPAR-a-Atgl cff; PPAR-a-Cpt2 cff; PPAR-a-ff:ff; PPAR-a-ff:ff Cre
N-methyl-GABA	34.977	6.66E-10	1.19E-08	Cpt2 cff-Atgl cff; PPAR-a-Atgl cff; ff:ff-Cpt2 cff; ff:ff Cre-Cpt2 cff; PPAR-a-ff:ff; PPAR-a-ff:ff Cre
ethylmalonate	34.3	8.17E-10	1.43E-08	Cpt2 cff-Atgl cff; ff:ff Cre-Atgl cff; PPAR-a-Atgl cff; ff:ff-Cpt2 cff; PPAR-a-Cpt2 cff; ff:ff Cre-ff:ff; PPAR-a-ff:ff; PPAR-a-ff:ff Cre
2-ketogulonate	32.799	1.30E-09	2.23E-08	PPAR-a-Atgl cff; PPAR-a-Cpt2 cff; PPAR-a-ff:ff; PPAR-a-ff:ff Cre
palmitoyl-myristoyl-glycerol160/1401	32.629	1.37E-09	2.30E-08	PPAR-a-Atgl cff; PPAR-a-Cpt2 cff; PPAR-a-ff:ff; PPAR-a-ff:ff Cre
4-cholesten-3-one	31.563	1.93E-09	3.18E-08	Cpt2 cff-Atgl cff; ff:ff Cre-Atgl cff; ff:ff Cre-Cpt2 cff; PPAR-a-Cpt2 cff; ff:ff Cre-ff:ff; PPAR-a-ff:ff Cre
glutaroylcarnitineC5	30.659	2.60E-09	4.20E-08	Cpt2 cff-Atgl cff; ff:ff Cre-Atgl cff; PPAR-a-Cpt2 cff; ff:ff Cre-ff:ff; PPAR-a-ff:ff; PPAR-a-ff:ff Cre
arabitol/xylitol	29.15	4.35E-09	6.88E-08	PPAR-a-Atgl cff; PPAR-a-Cpt2 cff; PPAR-a-ff:ff; PPAR-a-ff:ff Cre
stearoyl-linoleoyl-glycerol180/1822	28.102	6.30E-09	9.69E-08	PPAR-a-Atgl cff; PPAR-a-Cpt2 cff; PPAR-a-ff:ff; PPAR-a-ff:ff Cre
1-palmitoyl-2-palmitoleoyl-GPC160/161	28.032	6.46E-09	9.69E-08	Cpt2 cff-Atgl cff; ff:ff Cre-Atgl cff; ff:ff-Cpt2 cff; PPAR-a-Cpt2 cff; ff:ff Cre-ff:ff; PPAR-a-ff:ff Cre
sphingomyelind181/200d161/220	28.022	6.48E-09	9.69E-08	Cpt2 cff-Atgl cff; ff:ff Cre-Atgl cff; PPAR-a-Atgl cff; ff:ff-Cpt2 cff; PPAR-a-Cpt2 cff; ff:ff Cre-ff:ff; PPAR-a-ff:ff
N-linolenoyltaurine	27.406	8.10E-09	1.19E-07	PPAR-a-Atgl cff; PPAR-a-Cpt2 cff; PPAR-a-ff:ff; PPAR-a-ff:ff Cre
sphingomyelind182/230d181/231d171/241	27.141	8.93E-09	1.29E-07	Cpt2 cff-Atgl cff; ff:ff Cre-Atgl cff; PPAR-a-Atgl cff; ff:ff-Cpt2 cff; ff:ff Cre-ff:ff; PPAR-a-ff:ff
galactose1-phosphate	26.853	9.93E-09	1.41E-07	Cpt2 cff-Atgl cff; ff:ff Cre-Atgl cff; ff:ff-Cpt2 cff; PPAR-a-Cpt2 cff; ff:ff Cre-ff:ff; PPAR-a-ff:ff Cre
diacylglycerol120/181140/161160/1412	26.8	1.01E-08	1.41E-07	Cpt2 cff-Atgl cff; ff:ff Cre-Atgl cff; ff:ff-Cpt2 cff; ff:ff Cre-Cpt2 cff; PPAR-a-Cpt2 cff; ff:ff Cre-ff:ff; PPAR-a-ff:ff Cre
pyroglutamine	26.573	1.10E-08	1.51E-07	Cpt2 cff-Atgl cff; ff:ff Cre-Atgl cff; PPAR-a-Atgl cff; ff:ff-Cpt2 cff; PPAR-a-Cpt2 cff; ff:ff Cre-ff:ff; PPAR-a-ff:ff; PPAR-a-ff:ff Cre
1-palmitoyl-2-linoleoyl-GPG160/182	26.525	1.12E-08	1.51E-07	Cpt2 cff-Atgl cff; ff:ff Cre-Atgl cff; PPAR-a-Atgl cff; ff:ff-Cpt2 cff; ff:ff Cre-ff:ff; PPAR-a-ff:ff
margaroylcarnitineC17	26.348	1.20E-08	1.57E-07	Cpt2 cff-Atgl cff; ff:ff Cre-Atgl cff; ff:ff-Cpt2 cff; PPAR-a-Cpt2 cff; ff:ff Cre-ff:ff; PPAR-a-ff:ff Cre
beta-sitosterol	26.344	1.20E-08	1.57E-07	Cpt2 cff-Atgl cff; ff:ff Cre-Atgl cff; PPAR-a-Atgl cff; PPAR-a-Cpt2 cff; PPAR-a-ff:ff; PPAR-a-ff:ff Cre
citrate	25.768	1.50E-08	1.92E-07	PPAR-a-Atgl cff; PPAR-a-Cpt2 cff; PPAR-a-ff:ff; PPAR-a-ff:ff Cre
behenoylcarnitineC22	25.722	1.52E-08	1.93E-07	Cpt2 cff-Atgl cff; ff:ff Cre-Atgl cff; ff:ff-Cpt2 cff; PPAR-a-Cpt2 cff; ff:ff Cre-ff:ff; PPAR-a-ff:ff Cre
1-linoleoyl-GPS182	25.551	1.63E-08	2.03E-07	PPAR-a-Atgl cff; PPAR-a-Cpt2 cff; PPAR-a-ff:ff; PPAR-a-ff:ff Cre
1-palmitoyl-2-arachidonoyl-GPE160/204	25.258	1.82E-08	2.24E-07	Cpt2 cff-Atgl cff; PPAR-a-Atgl cff; ff:ff-Cpt2 cff; PPAR-a-Cpt2 cff; ff:ff Cre-ff:ff; PPAR-a-ff:ff; PPAR-a-ff:ff Cre
arachidoylcarnitineC20	25.042	1.98E-08	2.39E-07	Cpt2 cff-Atgl cff; ff:ff Cre-Atgl cff; ff:ff-Cpt2 cff; PPAR-a-Cpt2 cff; ff:ff Cre-ff:ff; PPAR-a-ff:ff Cre
daidzein	25.013	2.00E-08	2.39E-07	PPAR-a-Atgl cff; PPAR-a-Cpt2 cff; PPAR-a-ff:ff; PPAR-a-ff:ff Cre
octadecanedioateC18	24.901	2.10E-08	2.46E-07	Cpt2 cff-Atgl cff; ff:ff Cre-Atgl cff; PPAR-a-Atgl cff; ff:ff-Cpt2 cff; PPAR-a-Cpt2 cff; ff:ff Cre-ff:ff; PPAR-a-ff:ff; PPAR-a-ff:ff Cre
12-dipalmitoyl-GPC160/160	24.867	2.12E-08	2.46E-07	Cpt2 cff-Atgl cff; ff:ff Cre-Atgl cff; PPAR-a-Atgl cff; ff:ff-Cpt2 cff; ff:ff Cre-ff:ff; PPAR-a-ff:ff; PPAR-a-ff:ff Cre
ribulonate/xylulonate	24.786	2.19E-08	2.50E-07	ff:ff-Atgl cff; PPAR-a-Atgl cff; PPAR-a-Cpt2 cff; ff:ff Cre-ff:ff; PPAR-a-ff:ff; PPAR-a-ff:ff Cre
galactonate	24.561	2.40E-08	2.70E-07	PPAR-a-Atgl cff; PPAR-a-Cpt2 cff; PPAR-a-ff:ff; PPAR-a-ff:ff Cre
N-acetylglucosamine6-phosphate	24.504	2.45E-08	2.72E-07	Cpt2 cff-Atgl cff; ff:ff Cre-Atgl cff; PPAR-a-Atgl cff; ff:ff-Cpt2 cff; PPAR-a-Cpt2 cff; PPAR-a-ff:ff; PPAR-a-ff:ff Cre
palmitoyl-arachidonoyl-glycerol160/2042	24.212	2.76E-08	3.02E-07	Cpt2 cff-Atgl cff; ff:ff-Atgl cff; ff:ff Cre-Atgl cff; PPAR-a-Atgl cff; ff:ff-Cpt2 cff; ff:ff Cre-ff:ff; PPAR-a-ff:ff
glucose	23.867	3.17E-08	3.43E-07	Cpt2 cff-Atgl cff; ff:ff-Atgl cff; ff:ff Cre-Atgl cff; ff:ff-Cpt2 cff; PPAR-a-Cpt2 cff; ff:ff Cre-ff:ff; PPAR-a-ff:ff Cre
aconitatecisortrans	23.472	3.73E-08	3.98E-07	PPAR-a-Atgl cff; PPAR-a-Cpt2 cff; PPAR-a-ff:ff; PPAR-a-ff:ff Cre
pantothenateVitaminB5	23.379	3.87E-08	4.08E-07	PPAR-a-Atgl cff; PPAR-a-Cpt2 cff; PPAR-a-ff:ff; PPAR-a-ff:ff Cre
3-hydroxylaurate	23.301	4.00E-08	4.16E-07	PPAR-a-Atgl cff; PPAR-a-Cpt2 cff; PPAR-a-ff:ff; PPAR-a-ff:ff Cre
pimelateC7-DC	23.048	4.44E-08	4.57E-07	Cpt2 cff-Atgl cff; ff:ff Cre-Atgl cff; PPAR-a-Atgl cff; ff:ff-Cpt2 cff; ff:ff Cre-ff:ff; PPAR-a-ff:ff
1-linolenoyl-GPC183	22.899	4.73E-08	4.80E-07	ff:ff Cre-Atgl cff; PPAR-a-Atgl cff; PPAR-a-Cpt2 cff; ff:ff Cre-ff:ff; PPAR-a-ff:ff; PPAR-a-ff:ff Cre
tetradecadienoate142	22.709	5.13E-08	5.14E-07	Cpt2 cff-Atgl cff; ff:ff Cre-Atgl cff; ff:ff-Cpt2 cff; PPAR-a-Cpt2 cff; ff:ff Cre-ff:ff; PPAR-a-ff:ff; PPAR-a-ff:ff Cre
homostachydrine	22.575	5.42E-08	5.33E-07	Cpt2 cff-Atgl cff; ff:ff Cre-Atgl cff; ff:ff-Cpt2 cff; PPAR-a-Cpt2 cff; ff:ff Cre-ff:ff; PPAR-a-ff:ff Cre
adipate	22.565	5.45E-08	5.33E-07	ff:ff Cre-Atgl cff; PPAR-a-Atgl cff; PPAR-a-Cpt2 cff; PPAR-a-ff:ff; PPAR-a-ff:ff Cre
2-methylbutyrylcarnitineC5	22.319	6.05E-08	5.85E-07	Cpt2 cff-Atgl cff; ff:ff Cre-Atgl cff; PPAR-a-Atgl cff; ff:ff-Cpt2 cff; ff:ff Cre-ff:ff; PPAR-a-ff:ff
palmitoylcholine	22.125	6.58E-08	6.29E-07	Cpt2 cff-Atgl cff; ff:ff Cre-Atgl cff; PPAR-a-Atgl cff; ff:ff-Cpt2 cff; ff:ff Cre-ff:ff; PPAR-a-ff:ff
1-methyl-4-imidazoleacetate	21.999	6.95E-08	6.56E-07	PPAR-a-Atgl cff; PPAR-a-Cpt2 cff; PPAR-a-ff:ff; PPAR-a-ff:ff Cre
N-oleoyltaurine	21.804	7.56E-08	7.05E-07	PPAR-a-Atgl cff; PPAR-a-Cpt2 cff; PPAR-a-ff:ff; PPAR-a-ff:ff Cre
mannose	21.782	7.63E-08	7.05E-07	ff:ff-Atgl cff; ff:ff Cre-Atgl cff; PPAR-a-Atgl cff; ff:ff-Cpt2 cff; PPAR-a-Cpt2 cff; ff:ff Cre-ff:ff; PPAR-a-ff:ff Cre
1-oleoyl-2-docosahexaenoyl-GPC181/226	21.585	8.32E-08	7.60E-07	Cpt2 cff-Atgl cff; ff:ff Cre-Atgl cff; PPAR-a-Atgl cff; ff:ff-Cpt2 cff; PPAR-a-Cpt2 cff; ff:ff Cre-ff:ff; PPAR-a-ff:ff; PPAR-a-ff:ff Cre
nicotinamideribonucleotideNMN	21.459	8.80E-08	7.95E-07	Cpt2 cff-Atgl cff; ff:ff-Atgl cff; ff:ff Cre-Atgl cff; ff:ff-Cpt2 cff; PPAR-a-Cpt2 cff; ff:ff Cre-ff:ff; PPAR-a-ff:ff Cre
3-dephospho-CoA-glutathione	21.371	9.15E-08	8.17E-07	Cpt2 cff-Atgl cff; ff:ff Cre-Atgl cff; ff:ff-Cpt2 cff; PPAR-a-Cpt2 cff; ff:ff Cre-ff:ff; PPAR-a-ff:ff Cre
3-methylglutaconate	21.257	9.62E-08	8.50E-07	Cpt2 cff-Atgl cff; ff:ff Cre-Atgl cff; PPAR-a-Atgl cff; ff:ff-Cpt2 cff; ff:ff Cre-ff:ff; PPAR-a-ff:ff
linoleoyl-linoleoyl-glycerol182/1822	21.12	1.02E-07	8.94E-07	ff:ff Cre-Atgl cff; PPAR-a-Atgl cff; PPAR-a-Cpt2 cff; PPAR-a-ff:ff; PPAR-a-ff:ff Cre
suberoylcarnitineC8-DC	20.972	1.09E-07	9.45E-07	Cpt2 cff-Atgl cff; ff:ff Cre-Atgl cff; ff:ff-Cpt2 cff; PPAR-a-Cpt2 cff; ff:ff Cre-ff:ff; PPAR-a-ff:ff Cre
beta-alanine	20.799	1.18E-07	1.01E-06	Cpt2 cff-Atgl cff; ff:ff-Atgl cff; ff:ff Cre-Atgl cff; ff:ff-Cpt2 cff; ff:ff Cre-ff:ff; PPAR-a-ff:ff; PPAR-a-ff:ff Cre
sphingomyelind181/222d182/221d161/242	20.71	1.23E-07	1.04E-06	Cpt2 cff-Atgl cff; ff:ff Cre-Atgl cff; PPAR-a-Atgl cff; ff:ff-Cpt2 cff; ff:ff Cre-ff:ff; PPAR-a-ff:ff; PPAR-a-ff:ff Cre
N-behenoyl-sphingadienined182/220	20.535	1.33E-07	1.12E-06	Cpt2 cff-Atgl cff; ff:ff Cre-Atgl cff; PPAR-a-Atgl cff; ff:ff-Cpt2 cff; PPAR-a-Cpt2 cff; ff:ff Cre-ff:ff; PPAR-a-ff:ff; PPAR-a-ff:ff Cre
creatine	20.378	1.43E-07	1.19E-06	Cpt2 cff-Atgl cff; ff:ff Cre-Atgl cff; PPAR-a-Atgl cff; ff:ff-Cpt2 cff; PPAR-a-Cpt2 cff; ff:ff Cre-ff:ff; PPAR-a-ff:ff
3-aminoisobutyrate	20.361	1.44E-07	1.19E-06	ff:ff Cre-Atgl cff; PPAR-a-Atgl cff; PPAR-a-Cpt2 cff; ff:ff Cre-ff:ff; PPAR-a-ff:ff; PPAR-a-ff:ff Cre
NNN-trimethyl-5-aminovalerate	20.249	1.52E-07	1.24E-06	Cpt2 cff-Atgl cff; PPAR-a-Atgl cff; ff:ff-Cpt2 cff; ff:ff Cre-Cpt2 cff; PPAR-a-Cpt2 cff; PPAR-a-ff:ff; PPAR-a-ff:ff Cre
N-palmitoleoyltaurine	20.174	1.57E-07	1.27E-06	PPAR-a-Atgl cff; PPAR-a-Cpt2 cff; PPAR-a-ff:ff; PPAR-a-ff:ff Cre
behenoylsphingomyelind181/220	19.875	1.81E-07	1.44E-06	Cpt2 cff-Atgl cff; ff:ff Cre-Atgl cff; ff:ff-Cpt2 cff; PPAR-a-Cpt2 cff; ff:ff Cre-ff:ff; PPAR-a-ff:ff Cre
N4-acetylcytidine	19.852	1.83E-07	1.44E-06	PPAR-a-Atgl cff; PPAR-a-Cpt2 cff; PPAR-a-ff:ff; PPAR-a-ff:ff Cre
mannitol/sorbitol	19.804	1.87E-07	1.46E-06	Cpt2 cff-Atgl cff; ff:ff-Atgl cff; ff:ff Cre-Atgl cff; ff:ff-Cpt2 cff; ff:ff Cre-ff:ff; PPAR-a-ff:ff

CDP-ethanolamine	19.682	1.98E-07	1.53E-06	Cpt2 cff-Atgl cff; ff:ff Cre-Atgl cff; PPAR-a-Atgl cff; ff:ff-Cpt2 cff; ff:ff Cre-ff:ff; PPAR-a-ff:ff Cre
glycerophosphoglycerol	19.656	2.00E-07	1.54E-06	ff:ff-Atgl cff; PPAR-a-Atgl cff; ff:ff-Cpt2 cff; PPAR-a-Cpt2 cff; ff:ff Cre-ff:ff; PPAR-a-ff:ff; PPAR-a-ff:ff Cre
N-linoleoyltaurine	19.535	2.12E-07	1.61E-06	PPAR-a-Atgl cff; PPAR-a-Cpt2 cff; PPAR-a-ff:ff; PPAR-a-ff:ff Cre
glycerol3-phosphate	19.504	2.15E-07	1.62E-06	Cpt2 cff-Atgl cff; ff:ff Cre-Atgl cff; PPAR-a-Cpt2 cff; PPAR-a-ff:ff; PPAR-a-ff:ff Cre
NAD	19.409	2.25E-07	1.68E-06	Cpt2 cff-Atgl cff; ff:ff Cre-Atgl cff; PPAR-a-Atgl cff; ff:ff-Cpt2 cff; ff:ff Cre-ff:ff; PPAR-a-ff:ff
sphingomyelind181/210d171/220d161/230	19.202	2.48E-07	1.84E-06	Cpt2 cff-Atgl cff; ff:ff Cre-Atgl cff; ff:ff-Cpt2 cff; ff:ff Cre-ff:ff; PPAR-a-ff:ff; PPAR-a-ff:ff Cre
1-ribosyl-imidazoleacetate	19.115	2.59E-07	1.90E-06	Cpt2 cff-Atgl cff; ff:ff Cre-Atgl cff; PPAR-a-Atgl cff; ff:ff-Cpt2 cff; ff:ff Cre-Cpt2 cff; PPAR-a-Cpt2 cff; ff:ff Cre-ff:ff; PPAR-a-ff:ff
N6-carbamoylthreonyladenosine	18.937	2.82E-07	2.05E-06	PPAR-a-Atgl cff; PPAR-a-Cpt2 cff; PPAR-a-ff:ff; PPAR-a-ff:ff Cre
N-acetyl-isoputreanine	18.9	2.87E-07	2.07E-06	PPAR-a-Atgl cff; PPAR-a-Cpt2 cff; PPAR-a-ff:ff; PPAR-a-ff:ff Cre
diacylglycerol140/181160/1611	18.653	3.24E-07	2.32E-06	Cpt2 cff-Atgl cff; ff:ff Cre-Atgl cff; PPAR-a-Atgl cff; ff:ff-Cpt2 cff; ff:ff Cre-ff:ff; PPAR-a-ff:ff
ribitol	18.584	3.35E-07	2.38E-06	Cpt2 cff-Atgl cff; ff:ff Cre-Atgl cff; PPAR-a-Atgl cff; ff:ff-Cpt2 cff; ff:ff Cre-ff:ff; PPAR-a-ff:ff
3-hydroxyglutarate	18.526	3.45E-07	2.42E-06	PPAR-a-Atgl cff; PPAR-a-Cpt2 cff; PPAR-a-ff:ff; PPAR-a-ff:ff Cre
glutarateC5-DC	18.435	3.61E-07	2.51E-06	PPAR-a-Atgl cff; PPAR-a-Cpt2 cff; PPAR-a-ff:ff; PPAR-a-ff:ff Cre
oleoylcholine	18.343	3.78E-07	2.60E-06	Cpt2 cff-Atgl cff; PPAR-a-Atgl cff; ff:ff-Cpt2 cff; PPAR-a-Cpt2 cff; ff:ff Cre-ff:ff; PPAR-a-ff:ff; PPAR-a-ff:ff Cre
oleoyl-linoleoyl-glycerol181/1822	18.333	3.80E-07	2.60E-06	ff:ff-Atgl cff; ff:ff Cre-Atgl cff; PPAR-a-Cpt2 cff; PPAR-a-ff:ff; PPAR-a-ff:ff Cre
N-stearoyl-sphingosined181/180	18.303	3.85E-07	2.62E-06	Cpt2 cff-Atgl cff; ff:ff Cre-Atgl cff; PPAR-a-Atgl cff; ff:ff Cre-Cpt2 cff; ff:ff Cre-ff:ff; PPAR-a-ff:ff
sphingomyelind181/250d190/241d201/230d191/24	18.243	3.97E-07	2.67E-06	Cpt2 cff-Atgl cff; ff:ff Cre-Atgl cff; PPAR-a-Atgl cff; ff:ff-Cpt2 cff; ff:ff Cre-ff:ff; PPAR-a-ff:ff
sphingomyelind181/201d182/200	18.167	4.12E-07	2.75E-06	Cpt2 cff-Atgl cff; ff:ff Cre-Atgl cff; PPAR-a-Atgl cff; ff:ff-Cpt2 cff; ff:ff Cre-ff:ff
AMP	17.775	5.02E-07	3.33E-06	PPAR-a-Atgl cff; PPAR-a-Cpt2 cff; ff:ff Cre-ff:ff; PPAR-a-ff:ff; PPAR-a-ff:ff Cre
myristoyl-linoleoyl-glycerol140/1821	17.664	5.31E-07	3.49E-06	PPAR-a-Atgl cff; ff:ff-Cpt2 cff; PPAR-a-Cpt2 cff; PPAR-a-ff:ff; PPAR-a-ff:ff Cre
1-stearoyl-2-oleoyl-GPE180/181	17.631	5.40E-07	3.53E-06	PPAR-a-Atgl cff; PPAR-a-Cpt2 cff; PPAR-a-ff:ff; PPAR-a-ff:ff Cre
1-stearoyl-GPC180	17.542	5.65E-07	3.66E-06	PPAR-a-Atgl cff; PPAR-a-Cpt2 cff; PPAR-a-ff:ff; PPAR-a-ff:ff Cre
ceramided181/140d161/160	17.428	6.00E-07	3.85E-06	Cpt2 cff-Atgl cff; ff:ff Cre-Atgl cff; ff:ff-Cpt2 cff; ff:ff Cre-Cpt2 cff; ff:ff Cre-ff:ff; PPAR-a-ff:ff Cre
2-aminobutyrate	17.103	7.09E-07	4.52E-06	Cpt2 cff-Atgl cff; ff:ff Cre-Atgl cff; ff:ff-Cpt2 cff; PPAR-a-Cpt2 cff; ff:ff Cre-ff:ff; PPAR-a-ff:ff Cre
suberateC8-DC	17.075	7.20E-07	4.55E-06	Cpt2 cff-Atgl cff; ff:ff Cre-Atgl cff; ff:ff-Cpt2 cff; PPAR-a-Cpt2 cff; ff:ff Cre-ff:ff; PPAR-a-ff:ff Cre
imidazolelactate	16.863	8.05E-07	5.05E-06	PPAR-a-Atgl cff; PPAR-a-Cpt2 cff; PPAR-a-ff:ff; PPAR-a-ff:ff Cre
N-stearoyltaurine	16.629	9.10E-07	5.67E-06	PPAR-a-Atgl cff; PPAR-a-Cpt2 cff; PPAR-a-ff:ff; PPAR-a-ff:ff Cre
1-oleoyl-2-arachidonoyl-GPE181/204	16.599	9.25E-07	5.70E-06	Cpt2 cff-Atgl cff; ff:ff Cre-Atgl cff; PPAR-a-Atgl cff; ff:ff-Cpt2 cff; PPAR-a-Cpt2 cff; ff:ff Cre-ff:ff; PPAR-a-ff:ff; PPAR-a-ff:ff Cre
linoleoyl-linoleoyl-glycerol182/1821	16.59	9.29E-07	5.70E-06	ff:ff Cre-Atgl cff; PPAR-a-Cpt2 cff; PPAR-a-ff:ff; PPAR-a-ff:ff Cre
NNN-trimethyl-alanylprolinebetaineTMAP	16.465	9.93E-07	6.03E-06	PPAR-a-Atgl cff; PPAR-a-Cpt2 cff; PPAR-a-ff:ff; PPAR-a-ff:ff Cre
sphingomyelind181/140d161/160	16.459	9.97E-07	6.03E-06	ff:ff Cre-Atgl cff; ff:ff-Cpt2 cff; ff:ff Cre-Cpt2 cff; ff:ff Cre-ff:ff; PPAR-a-ff:ff Cre
1-linoleoyl-2-arachidonoyl-GPC182/204n6	16.31	1.08E-06	6.48E-06	Cpt2 cff-Atgl cff; ff:ff Cre-Atgl cff; PPAR-a-Atgl cff; ff:ff-Cpt2 cff; ff:ff Cre-ff:ff; PPAR-a-ff:ff
N-palmitoyltaurine	16.223	1.13E-06	6.72E-06	PPAR-a-Atgl cff; PPAR-a-Cpt2 cff; PPAR-a-ff:ff; PPAR-a-ff:ff Cre
3-methylcytidine	16.215	1.14E-06	6.72E-06	Cpt2 cff-Atgl cff; ff:ff Cre-Atgl cff; PPAR-a-Atgl cff; ff:ff-Cpt2 cff; ff:ff Cre-Cpt2 cff; PPAR-a-ff:ff
ribose1-phosphate	15.998	1.28E-06	7.51E-06	Cpt2 cff-Atgl cff; ff:ff Cre-Atgl cff; ff:ff-Cpt2 cff; PPAR-a-Cpt2 cff; ff:ff Cre-ff:ff; PPAR-a-ff:ff Cre
6-oxopiperidine-2-carboxylate	15.603	1.59E-06	9.27E-06	PPAR-a-Atgl cff; PPAR-a-Cpt2 cff; PPAR-a-ff:ff; PPAR-a-ff:ff Cre
erucoylcarnitineC221	15.569	1.62E-06	9.38E-06	Cpt2 cff-Atgl cff; ff:ff Cre-Atgl cff; ff:ff-Cpt2 cff; PPAR-a-Cpt2 cff; ff:ff Cre-ff:ff; PPAR-a-ff:ff Cre
uracil	15.465	1.72E-06	9.87E-06	Cpt2 cff-Atgl cff; ff:ff Cre-Atgl cff; ff:ff-Cpt2 cff; PPAR-a-Cpt2 cff; ff:ff Cre-ff:ff; PPAR-a-ff:ff Cre
quinolinate	15.324	1.86E-06	1.06E-05	Cpt2 cff-Atgl cff; ff:ff Cre-Atgl cff; ff:ff-Cpt2 cff; PPAR-a-Cpt2 cff; ff:ff Cre-ff:ff; PPAR-a-ff:ff Cre
1-palmitoyl-2-stearoyl-GPC160/180	15.276	1.91E-06	1.08E-05	Cpt2 cff-Atgl cff; ff:ff Cre-Atgl cff; ff:ff-Cpt2 cff; PPAR-a-Cpt2 cff; ff:ff Cre-ff:ff; PPAR-a-ff:ff Cre
phytosphingosine	15.056	2.16E-06	1.22E-05	PPAR-a-Atgl cff; PPAR-a-Cpt2 cff; PPAR-a-ff:ff; PPAR-a-ff:ff Cre
palmitoyl-linoleoyl-glycerol160/1821	15.037	2.19E-06	1.22E-05	ff:ff Cre-Atgl cff; PPAR-a-Atgl cff; PPAR-a-Cpt2 cff; PPAR-a-ff:ff; PPAR-a-ff:ff Cre
pimeloylcarnitine/3-methyladipoylcarnitineC7-DC	14.939	2.31E-06	1.28E-05	Cpt2 cff-Atgl cff; ff:ff Cre-Atgl cff; PPAR-a-Atgl cff; ff:ff-Cpt2 cff; ff:ff Cre-ff:ff; PPAR-a-ff:ff
N-arachidonoyltaurine	14.853	2.43E-06	1.34E-05	PPAR-a-Atgl cff; PPAR-a-Cpt2 cff; PPAR-a-ff:ff; PPAR-a-ff:ff Cre
nervonoylcarnitineC241	14.826	2.47E-06	1.35E-05	Cpt2 cff-Atgl cff; ff:ff Cre-Atgl cff; ff:ff-Cpt2 cff; PPAR-a-Cpt2 cff; ff:ff Cre-ff:ff; PPAR-a-ff:ff Cre
arabonate/xylonate	14.783	2.53E-06	1.38E-05	Cpt2 cff-Atgl cff; PPAR-a-Atgl cff; PPAR-a-Cpt2 cff; PPAR-a-ff:ff; PPAR-a-ff:ff Cre
glutamate	14.766	2.55E-06	1.38E-05	Cpt2 cff-Atgl cff; ff:ff Cre-Atgl cff; PPAR-a-Atgl cff; ff:ff-Cpt2 cff; ff:ff Cre-ff:ff; PPAR-a-ff:ff
sphingomyelind182/241d181/242	14.691	2.67E-06	1.43E-05	Cpt2 cff-Atgl cff; ff:ff Cre-Atgl cff; PPAR-a-Atgl cff; ff:ff-Cpt2 cff; ff:ff Cre-ff:ff; PPAR-a-ff:ff
linoleoyl-linolenoyl-glycerol182/1832	14.665	2.71E-06	1.44E-05	Cpt2 cff-Atgl cff; ff:ff-Atgl cff; ff:ff Cre-Atgl cff; PPAR-a-Cpt2 cff; PPAR-a-ff:ff; PPAR-a-ff:ff Cre
dihydrobiopterin	14.613	2.79E-06	1.48E-05	Cpt2 cff-Atgl cff; ff:ff Cre-Atgl cff; ff:ff-Cpt2 cff; PPAR-a-Cpt2 cff; ff:ff Cre-ff:ff; PPAR-a-ff:ff Cre
succinylcarnitineC4	14.365	3.22E-06	1.70E-05	ff:ff-Atgl cff; PPAR-a-Atgl cff; ff:ff-Cpt2 cff; PPAR-a-ff:ff; PPAR-a-ff:ff Cre
N-acetylneuraminate	14.307	3.34E-06	1.75E-05	Cpt2 cff-Atgl cff; ff:ff Cre-Atgl cff; ff:ff-Cpt2 cff; PPAR-a-Cpt2 cff; ff:ff Cre-ff:ff; PPAR-a-ff:ff Cre
2-palmitoleoyl-GPC161	14.188	3.58E-06	1.86E-05	Cpt2 cff-Atgl cff; ff:ff Cre-Atgl cff; PPAR-a-Cpt2 cff; ff:ff Cre-ff:ff; PPAR-a-ff:ff Cre
3-hydroxyhexanoate	14.055	3.87E-06	2.00E-05	PPAR-a-Atgl cff; PPAR-a-Cpt2 cff; PPAR-a-ff:ff; PPAR-a-ff:ff Cre
1-palmitoyl-2-oleoyl-GPE160/181	13.968	4.08E-06	2.09E-05	PPAR-a-Atgl cff; PPAR-a-Cpt2 cff; PPAR-a-ff:ff; PPAR-a-ff:ff Cre
sedoheptulose-7-phosphate	13.964	4.09E-06	2.09E-05	PPAR-a-Atgl cff; PPAR-a-Cpt2 cff; PPAR-a-ff:ff; PPAR-a-ff:ff Cre
N6-methyllysine	13.688	4.83E-06	2.45E-05	PPAR-a-Atgl cff; PPAR-a-Cpt2 cff; PPAR-a-ff:ff; PPAR-a-ff:ff Cre
maltose	13.542	5.28E-06	2.66E-05	ff:ff-Atgl cff; PPAR-a-Atgl cff; ff:ff-Cpt2 cff; PPAR-a-Cpt2 cff; ff:ff Cre-ff:ff; PPAR-a-ff:ff Cre
inosine	13.495	5.43E-06	2.72E-05	Cpt2 cff-Atgl cff; ff:ff Cre-Atgl cff; PPAR-a-Atgl cff; ff:ff-Cpt2 cff; ff:ff Cre-ff:ff; PPAR-a-ff:ff
1-lignoceroyl-GPC240	13.409	5.73E-06	2.85E-05	Cpt2 cff-Atgl cff; ff:ff Cre-Atgl cff; ff:ff-Cpt2 cff; PPAR-a-Cpt2 cff; ff:ff Cre-ff:ff; PPAR-a-ff:ff Cre
1-linoleoyl-GPE182	13.23	6.40E-06	3.17E-05	Cpt2 cff-Atgl cff; PPAR-a-Atgl cff; ff:ff-Cpt2 cff; PPAR-a-ff:ff; PPAR-a-ff:ff Cre
1920-DiHDPA	13.119	6.85E-06	3.37E-05	PPAR-a-Atgl cff; PPAR-a-Cpt2 cff; PPAR-a-ff:ff; PPAR-a-ff:ff Cre
serotonin	12.938	7.67E-06	3.75E-05	Cpt2 cff-Atgl cff; ff:ff Cre-Atgl cff; PPAR-a-Cpt2 cff; PPAR-a-ff:ff; PPAR-a-ff:ff Cre
stachydrine	12.928	7.72E-06	3.76E-05	Cpt2 cff-Atgl cff; ff:ff-Cpt2 cff; ff:ff Cre-Cpt2 cff; PPAR-a-Cpt2 cff
2-aminoadipate	12.913	7.80E-06	3.77E-05	PPAR-a-Atgl cff; PPAR-a-Cpt2 cff; PPAR-a-ff:ff; PPAR-a-ff:ff Cre
cystathionine	12.728	8.76E-06	4.21E-05	Cpt2 cff-Atgl cff; ff:ff Cre-Atgl cff; ff:ff-Cpt2 cff; PPAR-a-Cpt2 cff; ff:ff Cre-ff:ff; PPAR-a-ff:ff Cre
diacylglycerol140/181160/1612	12.674	9.07E-06	4.33E-05	ff:ff Cre-Atgl cff; ff:ff-Cpt2 cff; PPAR-a-Cpt2 cff; ff:ff Cre-ff:ff; PPAR-a-ff:ff Cre
pipecolate	12.631	9.33E-06	4.43E-05	Cpt2 cff-Atgl cff; ff:ff-Cpt2 cff; ff:ff Cre-Cpt2 cff; PPAR-a-Cpt2 cff
arachidonoylcarnitineC204	12.604	9.49E-06	4.48E-05	Cpt2 cff-Atgl cff; ff:ff-Atgl cff; ff:ff Cre-Atgl cff; PPAR-a-Atgl cff
1-stearoyl-2-arachidonoyl-GPS180/204	12.586	9.60E-06	4.49E-05	Cpt2 cff-Atgl cff; ff:ff Cre-Atgl cff; ff:ff-Cpt2 cff; PPAR-a-Cpt2 cff; ff:ff Cre-ff:ff
sphingomyelind182/231	12.582	9.62E-06	4.49E-05	Cpt2 cff-Atgl cff; ff:ff Cre-Atgl cff; PPAR-a-Atgl cff; ff:ff-Cpt2 cff; ff:ff Cre-ff:ff; PPAR-a-ff:ff
pyridoxate	12.571	9.69E-06	4.50E-05	Cpt2 cff-Atgl cff; ff:ff Cre-Atgl cff; PPAR-a-Atgl cff; ff:ff-Cpt2 cff; ff:ff Cre-ff:ff; PPAR-a-ff:ff
thymine	12.519	1.00E-05	4.62E-05	ff:ff-Atgl cff; PPAR-a-Atgl cff; ff:ff-Cpt2 cff; PPAR-a-Cpt2 cff; ff:ff Cre-ff:ff; PPAR-a-ff:ff Cre
N-acetylglutamate	12.374	1.10E-05	5.05E-05	Cpt2 cff-Atgl cff; ff:ff Cre-Atgl cff; PPAR-a-Atgl cff; ff:ff-Cpt2 cff; ff:ff Cre-ff:ff
hydroxyproline	12.286	1.16E-05	5.32E-05	PPAR-a-Atgl cff; PPAR-a-Cpt2 cff; PPAR-a-ff:ff; PPAR-a-ff:ff Cre
fructose	12.233	1.20E-05	5.47E-05	PPAR-a-Atgl cff; PPAR-a-Cpt2 cff; PPAR-a-ff:ff Cre
24-dihydroxybutyrate	12.191	1.24E-05	5.59E-05	PPAR-a-Atgl cff; PPAR-a-Cpt2 cff; PPAR-a-ff:ff; PPAR-a-ff:ff Cre
N-acetylglucosaminylasparagine	12.079	1.33E-05	5.96E-05	PPAR-a-Atgl cff; PPAR-a-Cpt2 cff; PPAR-a-ff:ff; PPAR-a-ff:ff Cre
glycosyl-N-stearoyl-sphingosined181/180	12.078	1.33E-05	5.96E-05	ff:ff Cre-Atgl cff; ff:ff Cre-Cpt2 cff; ff:ff Cre-ff:ff; PPAR-a-ff:ff Cre
gluconate	12.048	1.36E-05	6.04E-05	Cpt2 cff-Atgl cff; ff:ff Cre-Atgl cff; ff:ff-Cpt2 cff; PPAR-a-Cpt2 cff; ff:ff Cre-ff:ff
beta-guanidinopropanoate	12.033	1.37E-05	6.05E-05	Cpt2 cff-Atgl cff; PPAR-a-Atgl cff; ff:ff-Cpt2 cff; PPAR-a-ff:ff; PPAR-a-ff:ff Cre
N-acetyl-glucosamine1-phosphate	12.03	1.38E-05	6.05E-05	PPAR-a-Atgl cff; PPAR-a-Cpt2 cff; PPAR-a-ff:ff; PPAR-a-ff:ff Cre
3-hydroxyadipate	12.013	1.39E-05	6.09E-05	PPAR-a-Atgl cff; PPAR-a-Cpt2 cff; PPAR-a-ff:ff; PPAR-a-ff:ff Cre
alpha-ketoglutarate	11.972	1.43E-05	6.22E-05	Cpt2 cff-Atgl cff; ff:ff Cre-Atgl cff; ff:ff-Cpt2 cff; ff:ff Cre-ff:ff
5-methyluridineribothymidine	11.954	1.45E-05	6.26E-05	ff:ff-Atgl cff; ff:ff Cre-Atgl cff; ff:ff-Cpt2 cff; ff:ff Cre-ff:ff; PPAR-a-ff:ff
1-oleoyl-2-docosahexaenoyl-GPE181/226	11.895	1.51E-05	6.48E-05	PPAR-a-Atgl cff; ff:ff-Cpt2 cff; PPAR-a-Cpt2 cff; ff:ff Cre-ff:ff; PPAR-a-ff:ff
palmitoyldihydrosphingomyelind180/160	11.873	1.53E-05	6.54E-05	Cpt2 cff-Atgl cff; ff:ff-Cpt2 cff; ff:ff Cre-Cpt2 cff; PPAR-a-Cpt2 cff; ff:ff Cre-ff:ff
1415-DiHETrE	11.852	1.55E-05	6.57E-05	Cpt2 cff-Atgl cff; PPAR-a-Atgl cff; PPAR-a-Cpt2 cff; PPAR-a-ff:ff; PPAR-a-ff:ff Cre
cis-4-decenoate101n6	11.85	1.55E-05	6.57E-05	Cpt2 cff-Atgl cff; ff:ff-Cpt2 cff; PPAR-a-Cpt2 cff; ff:ff Cre-ff:ff; PPAR-a-ff:ff Cre
oleoyl-linoleoyl-glycerol181/1821	11.829	1.57E-05	6.63E-05	ff:ff Cre-Atgl cff; PPAR-a-Atgl cff; PPAR-a-Cpt2 cff; PPAR-a-ff:ff; PPAR-a-ff:ff Cre
pyridoxal	11.775	1.63E-05	6.84E-05	Cpt2 cff-Atgl cff; ff:ff Cre-Atgl cff; PPAR-a-Atgl cff
tetradecanedioateC14	11.621	1.81E-05	7.54E-05	Cpt2 cff-Atgl cff; ff:ff Cre-Atgl cff; PPAR-a-Atgl cff; ff:ff-Cpt2 cff; ff:ff Cre-ff:ff; PPAR-a-ff:ff
octadecenedioateC181-DC	11.602	1.83E-05	7.60E-05	PPAR-a-Atgl cff; PPAR-a-Cpt2 cff; PPAR-a-ff:ff; PPAR-a-ff:ff Cre
xanthosine	11.432	2.05E-05	8.48E-05	Cpt2 cff-Atgl cff; ff:ff Cre-Atgl cff; PPAR-a-Atgl cff; ff:ff-Cpt2 cff; ff:ff Cre-ff:ff; PPAR-a-ff:ff
erythronate	11.379	2.13E-05	8.75E-05	ff:ff-Atgl cff; ff:ff-Cpt2 cff; PPAR-a-Cpt2 cff; ff:ff Cre-ff:ff; PPAR-a-ff:ff Cre
N-acetylserine	11.368	2.14E-05	8.77E-05	PPAR-a-Atgl cff; PPAR-a-Cpt2 cff; ff:ff Cre-ff:ff; PPAR-a-ff:ff; PPAR-a-ff:ff Cre
2-deoxyinosine	11.309	2.23E-05	9.09E-05	Cpt2 cff-Atgl cff; ff:ff-Cpt2 cff; PPAR-a-Cpt2 cff; ff:ff Cre-ff:ff; PPAR-a-ff:ff Cre
chenodeoxycholate	11.283	2.27E-05	9.20E-05	Cpt2 cff-Atgl cff; ff:ff Cre-Atgl cff; PPAR-a-Atgl cff; ff:ff-Cpt2 cff; ff:ff Cre-ff:ff; PPAR-a-ff:ff
campesterol	11.276	2.28E-05	9.21E-05	Cpt2 cff-Atgl cff; ff:ff Cre-Atgl cff; ff:ff-Cpt2 cff; PPAR-a-Cpt2 cff; ff:ff Cre-ff:ff; PPAR-a-ff:ff Cre
3-hydroxysuberate	11.195	2.42E-05	9.69E-05	Cpt2 cff-Atgl cff; ff:ff Cre-Atgl cff; PPAR-a-Atgl cff; ff:ff-Cpt2 cff; ff:ff Cre-ff:ff; PPAR-a-ff:ff
palmitoleoyl-linoleoyl-glycerol161/1821	11.109	2.56E-05	0.00010212	Cpt2 cff-Atgl cff; ff:ff-Atgl cff; ff:ff-Cpt2 cff; ff:ff Cre-ff:ff; PPAR-a-ff:ff
adenine	11.104	2.57E-05	0.00010212	PPAR-a-Cpt2 cff; PPAR-a-ff:ff; PPAR-a-ff:ff Cre
ceramided181/170d171/180	11.072	2.63E-05	0.00010391	ff:ff Cre-Atgl cff; PPAR-a-Atgl cff; PPAR-a-Cpt2 cff; ff:ff Cre-ff:ff; PPAR-a-ff:ff
ophthalmate	10.944	2.87E-05	0.00011298	ff:ff-Cpt2 cff; PPAR-a-Cpt2 cff; ff:ff Cre-ff:ff; PPAR-a-ff:ff Cre
CoA-glutathione	10.937	2.89E-05	0.00011306	Cpt2 cff-Atgl cff; ff:ff Cre-Atgl cff; PPAR-a-Cpt2 cff; PPAR-a-ff:ff Cre
palmitoloelycholine	10.896	2.97E-05	0.00011576	Cpt2 cff-Atgl cff; ff:ff Cre-Atgl cff; PPAR-a-Atgl cff; ff:ff-Cpt2 cff; ff:ff Cre-ff:ff; PPAR-a-ff:ff

4-methylhexanoylglycine	10.879	3.01E-05	0.00011655	Cpt2 cff-Atgl cff; ff:ff Cre-Atgl cff; ff:ff-Cpt2 cff; ff:ff Cre-ff:ff; PPAR-a-ff:ff
N-formylmethionine	10.866	3.03E-05	0.00011711	Cpt2 cff-Atgl cff; ff:ff-Cpt2 cff; ff:ff Cre-Cpt2 cff; PPAR-a-Cpt2 cff
isovalerylcarnitineC5	10.845	3.08E-05	0.00011814	Cpt2 cff-Atgl cff; ff:ff Cre-Atgl cff; PPAR-a-Atgl cff; ff:ff-Cpt2 cff; ff:ff Cre-ff:ff; PPAR-a-ff:ff
threonate	10.84	3.09E-05	0.00011814	PPAR-a-Atgl cff; PPAR-a-Cpt2 cff; PPAR-a-ff:ff; PPAR-a-ff:ff Cre
palmitoleoylcarnitineC161	10.795	3.19E-05	0.00012139	Cpt2 cff-Atgl cff; ff:ff Cre-Atgl cff; ff:ff-Cpt2 cff; PPAR-a-Cpt2 cff; ff:ff Cre-ff:ff; PPAR-a-ff:ff Cre
UDP-galactose	10.721	3.36E-05	0.00012727	ff:ff Cre-Atgl cff; PPAR-a-Cpt2 cff; PPAR-a-ff:ff; PPAR-a-ff:ff Cre
tyrosine	10.692	3.43E-05	0.00012931	PPAR-a-Atgl cff; PPAR-a-Cpt2 cff; PPAR-a-ff:ff; PPAR-a-ff:ff Cre
oleoyl-oleoyl-glycerol181/1811	10.642	3.55E-05	0.00013338	PPAR-a-Atgl cff; PPAR-a-Cpt2 cff; PPAR-a-ff:ff; PPAR-a-ff:ff Cre
linoleoyl-docosahexaenoyl-glycerol182/2262	10.634	3.57E-05	0.00013357	Cpt2 cff-Atgl cff; ff:ff Cre-Atgl cff; PPAR-a-Atgl cff; ff:ff-Cpt2 cff; ff:ff Cre-ff:ff
N-acetylglucosamine/N-acetylgalactosamine	10.506	3.91E-05	0.0001456	Cpt2 cff-Atgl cff; ff:ff Cre-Atgl cff; PPAR-a-Atgl cff; ff:ff-Cpt2 cff; ff:ff Cre-ff:ff
3-phosphoglycerate	10.488	3.96E-05	0.00014681	PPAR-a-Atgl cff; PPAR-a-Cpt2 cff; PPAR-a-ff:ff; PPAR-a-ff:ff Cre
malate	10.306	4.52E-05	0.00016664	Cpt2 cff-Atgl cff; ff:ff Cre-Atgl cff; ff:ff-Cpt2 cff; PPAR-a-Cpt2 cff; ff:ff Cre-ff:ff; PPAR-a-ff:ff Cre
1-palmitoyl-2-arachidonoyl-GPI160/204	10.245	4.72E-05	0.00017332	ff:ff Cre-Atgl cff; ff:ff-Cpt2 cff; ff:ff Cre-ff:ff; PPAR-a-ff:ff
S-3-hydroxybutyrylcarnitine	10.189	4.92E-05	0.00017968	Cpt2 cff-Atgl cff; ff:ff Cre-Atgl cff; ff:ff-Cpt2 cff; PPAR-a-Cpt2 cff; ff:ff Cre-ff:ff; PPAR-a-ff:ff Cre
2-hydroxyadipate	10.167	5.00E-05	0.00018186	PPAR-a-Atgl cff; PPAR-a-Cpt2 cff; PPAR-a-ff:ff; PPAR-a-ff:ff Cre
indolelactate	10.147	5.07E-05	0.00018363	Cpt2 cff-Atgl cff; ff:ff Cre-Atgl cff; PPAR-a-Atgl cff; ff:ff-Cpt2 cff; ff:ff Cre-ff:ff
oleoyl-oleoyl-glycerol181/1812	10.052	5.44E-05	0.0001961	Cpt2 cff-Atgl cff; ff:ff-Atgl cff; ff:ff Cre-Atgl cff; PPAR-a-Cpt2 cff; PPAR-a-ff:ff; PPAR-a-ff:ff Cre
isobutyrylcarnitineC4	9.9925	5.68E-05	0.0002039	ff:ff-Cpt2 cff; ff:ff Cre-ff:ff; PPAR-a-ff:ff
nicotinicacidmononucleotideNaMN	9.9139	6.02E-05	0.00021512	Cpt2 cff-Atgl cff; ff:ff Cre-Atgl cff; ff:ff-Cpt2 cff; PPAR-a-Cpt2 cff; PPAR-a-ff:ff Cre
hexadecanedioateC16	9.8012	6.54E-05	0.00023285	Cpt2 cff-Atgl cff; ff:ff Cre-Atgl cff; PPAR-a-Atgl cff; ff:ff-Cpt2 cff; ff:ff Cre-ff:ff; PPAR-a-ff:ff
guanosine5-diphospho-fucose	9.794	6.58E-05	0.00023308	Cpt2 cff-Atgl cff; ff:ff-Cpt2 cff; PPAR-a-Cpt2 cff
caprylate80	9.7468	6.81E-05	0.0002404	Cpt2 cff-Atgl cff; ff:ff Cre-Atgl cff; ff:ff-Cpt2 cff; PPAR-a-Cpt2 cff; ff:ff Cre-ff:ff; PPAR-a-ff:ff Cre
5-methyl-2-deoxycytidine	9.6999	7.06E-05	0.00024789	PPAR-a-Atgl cff; ff:ff-Cpt2 cff; PPAR-a-Cpt2 cff; ff:ff Cre-ff:ff; PPAR-a-ff:ff Cre
butyrylglycineC4	9.6802	7.16E-05	0.00025051	Cpt2 cff-Atgl cff; ff:ff Cre-Atgl cff; ff:ff-Cpt2 cff; PPAR-a-Cpt2 cff; ff:ff Cre-ff:ff; PPAR-a-ff:ff Cre
oxalateethanedioate	9.6713	7.21E-05	0.00025099	Cpt2 cff-Atgl cff; PPAR-a-Atgl cff; ff:ff-Cpt2 cff; ff:ff Cre-Cpt2 cff
1-myristoyl-2-arachidonoyl-GPC140/204	9.6663	7.24E-05	0.00025099	ff:ff Cre-Atgl cff; PPAR-a-Atgl cff; PPAR-a-Cpt2 cff; PPAR-a-ff:ff
FAD	9.5794	7.73E-05	0.0002668	PPAR-a-Atgl cff; PPAR-a-Cpt2 cff; PPAR-a-ff:ff; PPAR-a-ff:ff Cre
isoleucylglycine	9.4844	8.30E-05	0.00028545	ff:ff-Atgl cff; PPAR-a-Cpt2 cff; PPAR-a-ff:ff; PPAR-a-ff:ff Cre
linoleoylcholine	9.4756	8.36E-05	0.00028616	PPAR-a-Atgl cff; PPAR-a-Cpt2 cff; PPAR-a-ff:ff; PPAR-a-ff:ff Cre
sphingomyelind171/160d181/150d161/170	9.4421	8.57E-05	0.00029232	Cpt2 cff-Atgl cff; ff:ff Cre-Atgl cff; PPAR-a-Cpt2 cff; PPAR-a-ff:ff Cre
eicosenoylcarnitineC201	9.4061	8.81E-05	0.00029918	Cpt2 cff-Atgl cff; ff:ff-Cpt2 cff; PPAR-a-Cpt2 cff; PPAR-a-ff:ff Cre
pterin	9.3455	9.22E-05	0.00031205	Cpt2 cff-Atgl cff; ff:ff Cre-Atgl cff; PPAR-a-Cpt2 cff; PPAR-a-ff:ff Cre
argininate	9.2724	9.76E-05	0.00032865	Cpt2 cff-Atgl cff; PPAR-a-Atgl cff; ff:ff-Cpt2 cff; ff:ff Cre-Cpt2 cff; PPAR-a-ff:ff
N-acetylthreonine	9.1543	0.00010684	0.00035845	PPAR-a-Atgl cff; PPAR-a-Cpt2 cff; PPAR-a-ff:ff; PPAR-a-ff:ff Cre
1-arachidonoyl-GPC204	9.1339	0.00010853	0.00036201	Cpt2 cff-Atgl cff; PPAR-a-Atgl cff; ff:ff-Cpt2 cff; PPAR-a-ff:ff
dimethylglycine	9.131	0.00010878	0.00036201	Cpt2 cff-Atgl cff; ff:ff Cre-Atgl cff; ff:ff Cre-ff:ff; PPAR-a-ff:ff Cre
1-methylnicotinamide	9.1102	0.00011054	0.00036639	PPAR-a-Atgl cff; PPAR-a-Cpt2 cff; PPAR-a-ff:ff; PPAR-a-ff:ff Cre
N-acetyltaurine	9.0801	0.00011315	0.00037354	Cpt2 cff-Atgl cff; ff:ff Cre-Atgl cff; ff:ff-Cpt2 cff; PPAR-a-Cpt2 cff; ff:ff Cre-ff:ff; PPAR-a-ff:ff Cre
S-methylglutathione	9.0195	0.00011861	0.00038883	ff:ff Cre-Atgl cff; PPAR-a-Cpt2 cff; PPAR-a-ff:ff; PPAR-a-ff:ff Cre
2-hydroxybutyrate/2-hydroxyisobutyrate	9.0182	0.00011873	0.00038883	Cpt2 cff-Atgl cff; ff:ff Cre-Atgl cff; PPAR-a-Cpt2 cff; ff:ff Cre-ff:ff; PPAR-a-ff:ff Cre
UDP-N-acetylglucosamine/galactosamine	8.9901	0.00012136	0.00039586	Cpt2 cff-Atgl cff; ff:ff Cre-Atgl cff; PPAR-a-Atgl cff; ff:ff-Cpt2 cff; ff:ff Cre-ff:ff; PPAR-a-ff:ff
sedoheptulose	8.9691	0.00012336	0.00039941	ff:ff-Cpt2 cff; ff:ff Cre-ff:ff; PPAR-a-ff:ff Cre
sphingomyelind182/160d181/161	8.9685	0.00012342	0.00039941	Cpt2 cff-Atgl cff; ff:ff Cre-Atgl cff; PPAR-a-Cpt2 cff; PPAR-a-ff:ff Cre
N-delta-acetylornithine	8.962	0.00012405	0.00039989	PPAR-a-Atgl cff; PPAR-a-Cpt2 cff; PPAR-a-ff:ff; PPAR-a-ff:ff Cre
1-linoleoyl-2-arachidonoyl-GPE182/204	8.9345	0.00012674	0.0004064	ff:ff Cre-Atgl cff; PPAR-a-Cpt2 cff; PPAR-a-ff:ff; PPAR-a-ff:ff Cre
2-oxoarginine	8.9313	0.00012706	0.0004064	Cpt2 cff-Atgl cff; ff:ff-Cpt2 cff; PPAR-a-Cpt2 cff
N-erucoyl-sphingosined181/221	8.9182	0.00012838	0.00040901	Cpt2 cff-Atgl cff; ff:ff Cre-Atgl cff; ff:ff-Cpt2 cff; PPAR-a-Cpt2 cff; ff:ff Cre-ff:ff; PPAR-a-ff:ff Cre
UDP-glucose	8.9059	0.00012962	0.00041137	ff:ff Cre-Atgl cff; ff:ff-Cpt2 cff; PPAR-a-Cpt2 cff; ff:ff Cre-ff:ff; PPAR-a-ff:ff Cre
N6-methyladenosine	8.8995	0.00013027	0.00041186	ff:ff-Cpt2 cff; PPAR-a-Cpt2 cff; ff:ff Cre-ff:ff; PPAR-a-ff:ff Cre
3-methylhistidine	8.8943	0.0001308	0.00041196	Cpt2 cff-Atgl cff; PPAR-a-Atgl cff; ff:ff-Cpt2 cff; PPAR-a-ff:ff
1-1-enyl-stearoyl-2-arachidonoyl-GPEP-180/204	8.8536	0.00013506	0.00042372	Cpt2 cff-Atgl cff; ff:ff Cre-Atgl cff; PPAR-a-Atgl cff; ff:ff-Cpt2 cff
N-linoleoylglycine	8.8318	0.00013739	0.00042942	Cpt2 cff-Atgl cff; ff:ff Cre-Atgl cff; ff:ff-Cpt2 cff; ff:ff Cre-ff:ff
taurodeoxycholate	8.8067	0.00014013	0.00043628	PPAR-a-Atgl cff; ff:ff Cre-ff:ff; PPAR-a-ff:ff
4-guanidinobutanoate	8.802	0.00014065	0.00043628	Cpt2 cff-Atgl cff; PPAR-a-Atgl cff; PPAR-a-ff:ff; PPAR-a-ff:ff Cre
azelatenonanedioateC9	8.7679	0.00014449	0.00044652	Cpt2 cff-Atgl cff; ff:ff-Cpt2 cff; PPAR-a-Cpt2 cff
ribose	8.7297	0.00014893	0.0004585	ff:ff-Cpt2 cff; ff:ff Cre-ff:ff; PPAR-a-ff:ff
linolenoyl-linolenoyl-glycerol183/1832	8.7042	0.00015197	0.00046613	ff:ff-Atgl cff; ff:ff-Cpt2 cff; PPAR-a-Cpt2 cff; ff:ff Cre-ff:ff
14-HDoHE/17-HDoHE	8.5976	0.00016543	0.00050553	ff:ff-Atgl cff; ff:ff-Cpt2 cff; ff:ff Cre-ff:ff; PPAR-a-ff:ff
N2-methylguanosine	8.5912	0.00016629	0.00050626	Cpt2 cff-Atgl cff; ff:ff Cre-Atgl cff; PPAR-a-Cpt2 cff; PPAR-a-ff:ff Cre
biopterin	8.5795	0.00016784	0.0005091	ff:ff Cre-Atgl cff; ff:ff-Cpt2 cff; ff:ff Cre-ff:ff; PPAR-a-ff:ff Cre
3-hydroxypalmitate	8.5666	0.00016959	0.00051251	ff:ff Cre-Atgl cff; ff:ff Cre-ff:ff
putrescine	8.5566	0.00017095	0.00051474	Cpt2 cff-Atgl cff; ff:ff Cre-Atgl cff; PPAR-a-Atgl cff; ff:ff-Cpt2 cff; ff:ff Cre-ff:ff; PPAR-a-ff:ff
dodecenedioateC121-DC	8.5205	0.00017597	0.00052792	PPAR-a-Atgl cff; PPAR-a-Cpt2 cff; PPAR-a-ff:ff; PPAR-a-ff:ff Cre
2-hydroxyheptanoate	8.5023	0.00017857	0.00053376	Cpt2 cff-Atgl cff; ff:ff Cre-Atgl cff; PPAR-a-Cpt2 cff; PPAR-a-ff:ff Cre
caprate100	8.4592	0.00018487	0.00055058	ff:ff-Cpt2 cff; PPAR-a-Cpt2 cff; ff:ff Cre-ff:ff; PPAR-a-ff:ff Cre
diacylglycerol161/1822160/1831	8.4453	0.00018694	0.00055474	ff:ff-Atgl cff; ff:ff-Cpt2 cff; PPAR-a-Cpt2 cff; ff:ff Cre-ff:ff; PPAR-a-ff:ff Cre
cholesterol	8.439	0.0001879	0.0005556	Cpt2 cff-Atgl cff; ff:ff Cre-Atgl cff; ff:ff-Cpt2 cff; PPAR-a-Cpt2 cff; ff:ff Cre-ff:ff; PPAR-a-ff:ff Cre
linoleoylethanolamide	8.3118	0.00020829	0.00061366	PPAR-a-Atgl cff; PPAR-a-Cpt2 cff; PPAR-a-ff:ff; PPAR-a-ff:ff Cre
2S3R-dihydroxybutyrate	8.2781	0.00021408	0.00062848	Cpt2 cff-Atgl cff; ff:ff Cre-Atgl cff; PPAR-a-Cpt2 cff; PPAR-a-ff:ff Cre
gamma-glutamylglutamate	8.2127	0.00022583	0.0006606	Cpt2 cff-Atgl cff; ff:ff Cre-Atgl cff; ff:ff-Cpt2 cff; ff:ff Cre-ff:ff; PPAR-a-ff:ff
S-adenosylhomocysteineSAH	8.2049	0.00022728	0.00066209	Cpt2 cff-Atgl cff; ff:ff Cre-Atgl cff; PPAR-a-Atgl cff; ff:ff-Cpt2 cff; ff:ff Cre-ff:ff; PPAR-a-ff:ff
tryptophan	8.2013	0.00022795	0.00066209	PPAR-a-Atgl cff; ff:ff-Cpt2 cff; ff:ff Cre-ff:ff; PPAR-a-ff:ff
7-hydroxycholesterolalphaorbeta	8.1884	0.00023036	0.00066568	Cpt2 cff-Atgl cff; ff:ff-Atgl cff; PPAR-a-Cpt2 cff; PPAR-a-ff:ff
xanthopterin	8.186	0.00023081	0.00066568	Cpt2 cff-Atgl cff; PPAR-a-Atgl cff; PPAR-a-ff:ff
stearoylcholine	8.1818	0.00023161	0.00066568	ff:ff-Cpt2 cff; ff:ff Cre-ff:ff; PPAR-a-ff:ff
N2N2-dimethylguanosine	8.1606	0.00023567	0.00067498	Cpt2 cff-Atgl cff; ff:ff Cre-Atgl cff; PPAR-a-Cpt2 cff; PPAR-a-ff:ff Cre
6-phosphogluconate	8.1421	0.00023929	0.00068297	ff:ff-Atgl cff; ff:ff-Cpt2 cff; PPAR-a-Cpt2 cff; ff:ff Cre-ff:ff; PPAR-a-ff:ff Cre
1-palmitoyl-GPI160	8.1199	0.00024369	0.00069314	Cpt2 cff-Atgl cff; ff:ff Cre-Atgl cff; ff:ff-Cpt2 cff; ff:ff Cre-ff:ff; PPAR-a-ff:ff
ascorbateVitaminC	8.0795	0.00025195	0.00071414	PPAR-a-Atgl cff; PPAR-a-Cpt2 cff; PPAR-a-ff:ff; PPAR-a-ff:ff Cre
3-hydroxyoleate	8.0529	0.00025753	0.00072745	Cpt2 cff-Atgl cff; ff:ff Cre-Atgl cff; ff:ff Cre-ff:ff
fumarate	8.0378	0.00026077	0.0007341	Cpt2 cff-Atgl cff; ff:ff Cre-Atgl cff; ff:ff-Cpt2 cff; PPAR-a-Cpt2 cff; ff:ff Cre-ff:ff; PPAR-a-ff:ff Cre
4-hydroxycinnamatesulfate	8.0128	0.00026622	0.00074686	PPAR-a-Atgl cff; PPAR-a-Cpt2 cff; PPAR-a-ff:ff; PPAR-a-ff:ff Cre
1-palmitoyl-2-gamma-linolenoyl-GPC160/183n6	8	0.00026906	0.00075226	Cpt2 cff-Atgl cff; ff:ff Cre-Atgl cff; ff:ff-Cpt2 cff; PPAR-a-Cpt2 cff; ff:ff Cre-ff:ff
histidine	7.9931	0.00027061	0.00075405	PPAR-a-Atgl cff; PPAR-a-Cpt2 cff; PPAR-a-ff:ff Cre
glucuronate	7.9097	0.00029005	0.00080548	PPAR-a-Atgl cff; PPAR-a-Cpt2 cff; PPAR-a-ff:ff; PPAR-a-ff:ff Cre
N6-succinyladenosine	7.8861	0.00029582	0.00081873	PPAR-a-Atgl cff; PPAR-a-Cpt2 cff; PPAR-a-ff:ff Cre
riboflavinVitaminB2	7.7913	0.00032026	0.00088341	Cpt2 cff-Atgl cff; ff:ff Cre-Atgl cff; ff:ff-Cpt2 cff; ff:ff Cre-ff:ff
taurochenodeoxycholicacid7-sulfate	7.7671	0.00032685	0.00089858	Cpt2 cff-Atgl cff; ff:ff Cre-Atgl cff; PPAR-a-Atgl cff; ff:ff-Cpt2 cff; ff:ff Cre-ff:ff
stearoyl-arachidonoyl-glycerol180/2041	7.7395	0.00033455	0.00091668	Cpt2 cff-Atgl cff; ff:ff Cre-Atgl cff; ff:ff-Cpt2 cff; ff:ff Cre-ff:ff; PPAR-a-ff:ff Cre
nonanoylcarnitineC9	7.68	0.00035178	0.00096069	Cpt2 cff-Atgl cff; ff:ff Cre-Atgl cff; PPAR-a-Atgl cff
1-1-enyl-palmitoyl-2-linoleoyl-GPEP-160/182	7.6733	0.0003538	0.00096299	Cpt2 cff-Atgl cff; ff:ff Cre-Atgl cff; PPAR-a-Atgl cff; PPAR-a-ff:ff
threonine	7.6636	0.00035671	0.0009677	ff:ff-Atgl cff; PPAR-a-Cpt2 cff; PPAR-a-ff:ff; PPAR-a-ff:ff Cre
palmitoyl-oleoyl-glycerol160/1812	7.6568	0.00035875	0.00097004	Cpt2 cff-Atgl cff; ff:ff-Atgl cff; ff:ff Cre-Atgl cff; PPAR-a-ff:ff Cre
2-hydroxy-3-methylvalerate	7.6053	0.00037481	0.0010102	ff:ff Cre-Atgl cff; PPAR-a-Cpt2 cff; PPAR-a-ff:ff Cre
glycineconjugateofC10H14O21	7.6013	0.00037607	0.0010102	PPAR-a-Atgl cff; PPAR-a-Cpt2 cff; PPAR-a-ff:ff; PPAR-a-ff:ff Cre
palmitoyl-palmitoyl-glycerol160/1602	7.5789	0.00038333	0.0010264	PPAR-a-Cpt2 cff; PPAR-a-ff:ff; PPAR-a-ff:ff Cre
adenosine5-diphosphoriboseADP-ribose	7.5468	0.00039396	0.0010514	PPAR-a-Atgl cff; ff:ff-Cpt2 cff; PPAR-a-ff:ff
glycylleucine	7.5383	0.00039683	0.0010557	PPAR-a-Atgl cff; ff:ff Cre-Cpt2 cff; PPAR-a-Cpt2 cff; ff:ff Cre-ff:ff; PPAR-a-ff:ff
2-hydroxypalmitate	7.5276	0.00040047	0.0010619	PPAR-a-Atgl cff; PPAR-a-Cpt2 cff; PPAR-a-ff:ff; PPAR-a-ff:ff Cre
1-myristoyl-2-palmitoyl-GPC140/160	7.5091	0.00040685	0.0010732	PPAR-a-Atgl cff; PPAR-a-Cpt2 cff; PPAR-a-ff:ff; PPAR-a-ff:ff Cre
1-1-enyl-palmitoyl-2-palmitoyl-GPCP-160/160	7.5058	0.00040801	0.0010732	Cpt2 cff-Atgl cff; ff:ff Cre-Atgl cff; ff:ff-Cpt2 cff; ff:ff Cre-ff:ff
taurolithocholate	7.5039	0.00040867	0.0010732	Cpt2 cff-Atgl cff; ff:ff Cre-Atgl cff; PPAR-a-Atgl cff; ff:ff-Cpt2 cff; ff:ff Cre-ff:ff; PPAR-a-ff:ff
3-hydroxydodecanedioate	7.4867	0.00041472	0.0010857	PPAR-a-Atgl cff; PPAR-a-Cpt2 cff; PPAR-a-ff:ff; PPAR-a-ff:ff Cre
1-stearoyl-2-docosahexaenoyl-GPE180/226	7.4182	0.00043988	0.0011479	PPAR-a-Atgl cff; PPAR-a-ff:ff; PPAR-a-ff:ff Cre
stearoylsphingomyelind181/180	7.3253	0.00047658	0.0012397	ff:ff Cre-Atgl cff; ff:ff Cre-Cpt2 cff; ff:ff Cre-ff:ff; PPAR-a-ff:ff Cre
1-methyladenosine	7.3002	0.00048709	0.0012631	Cpt2 cff-Atgl cff; PPAR-a-Cpt2 cff; PPAR-a-ff:ff Cre

1213-DiHOME	7.2645	0.00050242	0.0012973	PPAR-a-Atgl cff; PPAR-a-Cpt2 cff; PPAR-a-ff:ff; PPAR-a-ff:ff Cre
pyridoxamine	7.2621	0.00050346	0.0012973	ff:ff Cre-Atgl cff; PPAR-a-Atgl cff; ff:ff Cre-ff:ff; PPAR-a-ff:ff
nicotinateribonucleoside	7.256	0.00050614	0.0013001	Cpt2 cff-Atgl cff; ff:ff Cre-Atgl cff; PPAR-a-Cpt2 cff; PPAR-a-ff:ff Cre
dodecanedioateC12	7.2404	0.00051306	0.0013138	PPAR-a-Atgl cff; PPAR-a-Cpt2 cff; PPAR-a-ff:ff; PPAR-a-ff:ff Cre
N-acetylcysteine	7.2229	0.00052095	0.0013264	Cpt2 cff-Atgl cff; ff:ff Cre-Atgl cff; PPAR-a-Atgl cff; PPAR-a-ff:ff
O-sulfo-L-tyrosine	7.2223	0.00052121	0.0013264	PPAR-a-Cpt2 cff; PPAR-a-ff:ff Cre
N-palmitoylglycine	7.209	0.0005273	0.0013378	Cpt2 cff-Atgl cff; ff:ff Cre-Atgl cff; PPAR-a-Atgl cff
glycine	7.2041	0.00052958	0.0013385	PPAR-a-Atgl cff; ff:ff Cre-ff:ff; PPAR-a-ff:ff
linoleoyl-linolenoyl-glycerol182/1831	7.2014	0.00053082	0.0013385	ff:ff-Atgl cff; ff:ff Cre-Atgl cff; PPAR-a-ff:ff; PPAR-a-ff:ff Cre
N-octanoylglycine	7.1925	0.00053498	0.0013448	ff:ff Cre-Atgl cff; PPAR-a-Cpt2 cff; PPAR-a-ff:ff Cre
N-acetylglutamine	7.1614	0.00054972	0.0013758	Cpt2 cff-Atgl cff; ff:ff Cre-Atgl cff; ff:ff-Cpt2 cff; ff:ff Cre-ff:ff
phosphate	7.1595	0.00055064	0.0013758	ff:ff-Cpt2 cff; ff:ff Cre-ff:ff
tiglylcarnitineC5	7.1292	0.00056544	0.0014084	PPAR-a-Atgl cff; ff:ff-Cpt2 cff; ff:ff Cre-ff:ff; PPAR-a-ff:ff
taurochenodeoxycholate	7.1201	0.00056997	0.0014144	Cpt2 cff-Atgl cff; ff:ff Cre-Atgl cff; PPAR-a-Atgl cff
1-palmitoyl-2-linoleoyl-GPC160/182	7.1175	0.00057127	0.0014144	PPAR-a-Cpt2 cff; ff:ff Cre-ff:ff; PPAR-a-ff:ff Cre
3-amino-2-piperidone	7.0971	0.00058161	0.0014332	PPAR-a-Cpt2 cff; PPAR-a-ff:ff
S-3-hydroxypropylmercapturicacidHPMA	7.0957	0.00058235	0.0014332	PPAR-a-Atgl cff; PPAR-a-Cpt2 cff; PPAR-a-ff:ff; PPAR-a-ff:ff Cre
pyridoxaminephosphate	7.0767	0.00059216	0.001453	PPAR-a-Atgl cff; PPAR-a-Cpt2 cff; PPAR-a-ff:ff Cre
sphingomyelind180/180d190/170	7.0143	0.00062565	0.0015306	ff:ff Cre-Cpt2 cff; PPAR-a-Cpt2 cff
glycylvaline	7.0061	0.00063021	0.0015372	PPAR-a-Cpt2 cff; ff:ff Cre-ff:ff; PPAR-a-ff:ff
stearoylcarnitineC18	6.973	0.00064897	0.0015783	Cpt2 cff-Atgl cff; ff:ff-Cpt2 cff; PPAR-a-Cpt2 cff; ff:ff Cre-ff:ff
betainealdehyde	6.8543	0.00072124	0.0017489	ff:ff-Atgl cff; ff:ff-Cpt2 cff; ff:ff Cre-ff:ff; PPAR-a-ff:ff
octadecadienedioateC182-DC	6.7934	0.00076171	0.0018395	PPAR-a-Atgl cff; PPAR-a-Cpt2 cff; PPAR-a-ff:ff; PPAR-a-ff:ff Cre
glutamine	6.7913	0.0007631	0.0018395	ff:ff-Atgl cff; PPAR-a-Atgl cff; ff:ff-Cpt2 cff; ff:ff Cre-ff:ff
sphingomyelind181/181d182/180	6.7646	0.00078166	0.0018787	PPAR-a-Cpt2 cff; PPAR-a-ff:ff; PPAR-a-ff:ff Cre
alpha-hydroxyisovalerate	6.7454	0.00079528	0.0019059	ff:ff Cre-Atgl cff; PPAR-a-ff:ff Cre
Isobarhexosediphosphates	6.5819	0.00092213	0.0022035	PPAR-a-Cpt2 cff; PPAR-a-ff:ff Cre
uridine5-diphosphateUDP	6.5741	0.00092866	0.0022126	Cpt2 cff-Atgl cff; ff:ff Cre-Atgl cff; PPAR-a-Atgl cff
glycosyl-N-nervonoyl-sphingosined181/241	6.5409	0.00095725	0.0022742	PPAR-a-Atgl cff; PPAR-a-Cpt2 cff; PPAR-a-ff:ff; PPAR-a-ff:ff Cre
stearoyl-docosahexaenoyl-glycerol180/2262	6.53	0.00096675	0.0022901	ff:ff-Atgl cff; ff:ff-Cpt2 cff; ff:ff Cre-ff:ff
cis-4-decenoylcarnitineC101	6.4681	0.0010231	0.0024166	Cpt2 cff-Atgl cff; ff:ff Cre-Atgl cff; ff:ff-Cpt2 cff; ff:ff Cre-ff:ff
1-arachidonoyl-GPI204	6.4583	0.0010324	0.0024315	PPAR-a-Atgl cff; ff:ff-Cpt2 cff; PPAR-a-ff:ff; PPAR-a-ff:ff Cre
N-acetyl-3-methylhistidine	6.4208	0.0010686	0.0025096	PPAR-a-Cpt2 cff; PPAR-a-ff:ff Cre
3-ureidopropionate	6.4128	0.0010765	0.002521	Cpt2 cff-Atgl cff; ff:ff-Cpt2 cff; PPAR-a-Cpt2 cff
N-acetyl-1-methylhistidine	6.3358	0.0011557	0.0026989	PPAR-a-Atgl cff; PPAR-a-Cpt2 cff; PPAR-a-ff:ff; PPAR-a-ff:ff Cre
1-oleoyl-GPI181	6.3145	0.0011788	0.0027449	ff:ff Cre-Atgl cff; PPAR-a-Atgl cff; ff:ff Cre-ff:ff
enterolactone	6.293	0.0012025	0.0027923	Cpt2 cff-Atgl cff; PPAR-a-Atgl cff; ff:ff-Cpt2 cff; PPAR-a-ff:ff
2-aminoheptanoate	6.2645	0.0012348	0.0028592	Cpt2 cff-Atgl cff; ff:ff-Cpt2 cff; ff:ff Cre-Cpt2 cff; PPAR-a-Cpt2 cff
oleoyl-arachidonoyl-glycerol181/2042	6.2261	0.0012798	0.0029551	Cpt2 cff-Atgl cff; ff:ff Cre-Atgl cff; PPAR-a-Atgl cff
maltotriose	6.1829	0.0013324	0.003068	PPAR-a-Atgl cff; PPAR-a-Cpt2 cff; PPAR-a-ff:ff Cre
3-hydroxydecanoate	6.1635	0.0013569	0.0031155	PPAR-a-Atgl cff; PPAR-a-ff:ff; PPAR-a-ff:ff Cre
1-oleoyl-2-arachidonoyl-GPI181/204	6.1525	0.0013709	0.003139	ff:ff Cre-Atgl cff; ff:ff Cre-ff:ff
stearoyl-arachidonoyl-glycerol180/2042	6.139	0.0013884	0.0031701	ff:ff-Cpt2 cff; PPAR-a-Cpt2 cff; ff:ff Cre-ff:ff; PPAR-a-ff:ff Cre
5-methylthioadenosineMTA	6.1086	0.0014287	0.0032531	PPAR-a-Cpt2 cff; PPAR-a-ff:ff; PPAR-a-ff:ff Cre
adenosine35-diphosphate	6.0898	0.0014541	0.0033019	Cpt2 cff-Atgl cff; ff:ff-Cpt2 cff
N-acetylmethionine	6.0839	0.0014623	0.0033112	Cpt2 cff-Atgl cff; PPAR-a-Cpt2 cff
1-palmitoyl-GPE160	6.0572	0.0014995	0.0033797	PPAR-a-Atgl cff; ff:ff-Cpt2 cff; ff:ff Cre-ff:ff; PPAR-a-ff:ff
1-palmitoyl-GPC160	6.0563	0.0015007	0.0033797	Cpt2 cff-Atgl cff; ff:ff-Cpt2 cff; ff:ff Cre-ff:ff
FMN	6.0369	0.0015286	0.003433	ff:ff-Atgl cff; ff:ff-Cpt2 cff; ff:ff Cre-ff:ff; PPAR-a-ff:ff
5-hydroxyhexanoate	6.0282	0.0015411	0.0034517	PPAR-a-Atgl cff; PPAR-a-Cpt2 cff; PPAR-a-ff:ff; PPAR-a-ff:ff Cre
5-methyltetrahydrofolate5MeTHF	5.9727	0.0016243	0.0036283	ff:ff-Atgl cff; ff:ff-Cpt2 cff; ff:ff Cre-ff:ff; PPAR-a-ff:ff
picolinate	5.9641	0.0016376	0.0036479	Cpt2 cff-Atgl cff; PPAR-a-Cpt2 cff; PPAR-a-ff:ff
corticosterone	5.9169	0.0017128	0.0038051	ff:ff Cre-Cpt2 cff; ff:ff Cre-ff:ff; PPAR-a-ff:ff Cre
sphingomyelind180/200d160/220	5.8717	0.0017883	0.0039622	PPAR-a-Cpt2 cff; PPAR-a-ff:ff Cre
N2N5-diacetylornithine	5.8677	0.0017952	0.0039668	PPAR-a-Atgl cff; PPAR-a-Cpt2 cff; PPAR-a-ff:ff Cre
N1-methylinosine	5.859	0.0018102	0.0039893	PPAR-a-Cpt2 cff; PPAR-a-ff:ff Cre
hypoxanthine	5.8553	0.0018166	0.0039927	ff:ff-Cpt2 cff; ff:ff Cre-ff:ff
sphingosine	5.8241	0.0018717	0.0041029	ff:ff Cre-Atgl cff; PPAR-a-Atgl cff; ff:ff Cre-ff:ff; PPAR-a-ff:ff
1-stearoyl-2-arachidonoyl-GPC180/204	5.7811	0.0019507	0.0042646	Cpt2 cff-Atgl cff
adenosine-5-diphosphoglucose	5.7745	0.0019631	0.0042804	Cpt2 cff-Atgl cff; ff:ff-Cpt2 cff; PPAR-a-Cpt2 cff
1-methylhistamine	5.7689	0.0019737	0.0042862	ff:ff-Atgl cff; ff:ff-Cpt2 cff; ff:ff Cre-ff:ff
biliverdin	5.7676	0.0019762	0.0042862	PPAR-a-Atgl cff; PPAR-a-Cpt2 cff; PPAR-a-ff:ff; PPAR-a-ff:ff Cre
glycosylceramided182/241d181/242	5.7646	0.0019818	0.0042869	PPAR-a-Cpt2 cff; PPAR-a-ff:ff Cre
hexadecadienoate162n6	5.7115	0.0020861	0.0044904	ff:ff-Cpt2 cff; PPAR-a-ff:ff
hexadecenedioateC161-DC	5.7112	0.0020868	0.0044904	PPAR-a-Atgl cff; PPAR-a-ff:ff
hexanoylglycineC6	5.699	0.0021114	0.004521	ff:ff Cre-Atgl cff; ff:ff-Cpt2 cff; ff:ff Cre-ff:ff
phenylalanine	5.6987	0.002112	0.004521	ff:ff Cre-ff:ff; PPAR-a-ff:ff
taurocyamine	5.6784	0.002154	0.0045989	Cpt2 cff-Atgl cff; ff:ff-Cpt2 cff
2-phosphoglycerate	5.6722	0.0021669	0.0046144	PPAR-a-Cpt2 cff; PPAR-a-ff:ff Cre
sphinganine	5.6085	0.0023053	0.0048965	ff:ff Cre-Atgl cff; ff:ff Cre-ff:ff
homocitrulline	5.5511	0.002438	0.005165	Cpt2 cff-Atgl cff; ff:ff-Cpt2 cff; ff:ff Cre-Cpt2 cff; PPAR-a-Cpt2 cff
1-1-enyl-stearoyl-2-linoleoyl-GPEP-180/182	5.5269	0.0024964	0.0052752	Cpt2 cff-Atgl cff; ff:ff Cre-Atgl cff; PPAR-a-Atgl cff
1-palmitoyl-2-oleoyl-GPG160/181	5.5147	0.0025266	0.0053143	ff:ff-Cpt2 cff; PPAR-a-ff:ff
phenyllactatePLA	5.5142	0.0025278	0.0053143	PPAR-a-Atgl cff; PPAR-a-Cpt2 cff; PPAR-a-ff:ff; PPAR-a-ff:ff Cre
S-adenosylmethionineSAM	5.5106	0.0025367	0.0053193	ff:ff Cre-Atgl cff; PPAR-a-Cpt2 cff; PPAR-a-ff:ff Cre
hyocholate	5.4301	0.0027457	0.005743	ff:ff Cre-Atgl cff; PPAR-a-Atgl cff
heptenedioateC71-DC	5.4259	0.002757	0.0057519	ff:ff-Cpt2 cff; PPAR-a-ff:ff; PPAR-a-ff:ff Cre
lactosyl-N-behenoyl-sphingosined181/220	5.402	0.0028229	0.0058745	PPAR-a-Cpt2 cff; PPAR-a-ff:ff
phosphoenolpyruvatePEP	5.3944	0.0028441	0.0058976	PPAR-a-Cpt2 cff; PPAR-a-ff:ff; PPAR-a-ff:ff Cre
guanidinosuccinate	5.3929	0.0028483	0.0058976	ff:ff Cre-Cpt2 cff; PPAR-a-ff:ff Cre
tartronatehydroxymalonate	5.3842	0.0028731	0.005934	PPAR-a-Atgl cff; PPAR-a-ff:ff; PPAR-a-ff:ff Cre
linoleoyl-arachidonoyl-glycerol182/2042	5.3486	0.002976	0.0061311	Cpt2 cff-Atgl cff; ff:ff-Atgl cff; ff:ff Cre-Atgl cff
asparagine	5.3171	0.0030705	0.00631	ff:ff-Atgl cff; PPAR-a-ff:ff
propionylcarnitineC3	5.3022	0.0031165	0.0063885	Cpt2 cff-Atgl cff; ff:ff Cre-Atgl cff; PPAR-a-Atgl cff
gamma-glutamyl-epsilon-lysine	5.2609	0.0032476	0.0066407	PPAR-a-Atgl cff; PPAR-a-ff:ff
palmitoylsphingomyelind181/160	5.2579	0.0032572	0.0066437	Cpt2 cff-Atgl cff; ff:ff-Cpt2 cff
o-Tyrosine	5.2523	0.0032755	0.0066645	Cpt2 cff-Atgl cff; ff:ff-Atgl cff; ff:ff Cre-Atgl cff
N-palmitoyl-sphingosined181/160	5.2339	0.0033362	0.0067712	PPAR-a-Atgl cff; PPAR-a-Cpt2 cff; PPAR-a-ff:ff
carboxyethyl-GABA	5.1961	0.0034652	0.0070157	Cpt2 cff-Atgl cff; ff:ff Cre-Atgl cff
suberylglycine	5.1879	0.0034935	0.0070557	ff:ff Cre-Atgl cff; PPAR-a-Atgl cff; ff:ff Cre-ff:ff
nicotinate	5.1749	0.0035395	0.007131	PPAR-a-Atgl cff; PPAR-a-ff:ff; PPAR-a-ff:ff Cre
12-HETE	5.1399	0.0036661	0.007368	ff:ff Cre-ff:ff; PPAR-a-ff:ff
taurohyodeoxycholicacid	5.1307	0.0037005	0.0074191	ff:ff-Atgl cff; PPAR-a-ff:ff
serine	5.1276	0.0037122	0.0074243	ff:ff-Atgl cff; PPAR-a-ff:ff
erucate221n9	5.1071	0.0037894	0.0075605	Cpt2 cff-Atgl cff; ff:ff-Atgl cff; ff:ff Cre-Atgl cff
isocaproylglycine	5.0723	0.0039253	0.0078126	ff:ff Cre-Atgl cff; ff:ff Cre-ff:ff
myristoyl-linoleoyl-glycerol140/1822	5.0314	0.0040914	0.0081235	ff:ff-Cpt2 cff; PPAR-a-ff:ff
N6-acetyllysine	5.0081	0.0041893	0.0082978	Cpt2 cff-Atgl cff; ff:ff-Cpt2 cff; ff:ff Cre-Cpt2 cff; PPAR-a-Cpt2 cff
indoleacetylglycine	4.9421	0.0044806	0.0088535	PPAR-a-Atgl cff; PPAR-a-Cpt2 cff; PPAR-a-ff:ff Cre
mannonate	4.9305	0.0045339	0.0089286	ff:ff-Cpt2 cff; ff:ff Cre-ff:ff; PPAR-a-ff:ff
7-methylguanine	4.9291	0.0045403	0.0089286	PPAR-a-Cpt2 cff; PPAR-a-ff:ff Cre
3-5-adenylyladenosine	4.8912	0.0047202	0.0092602	Cpt2 cff-Atgl cff; ff:ff Cre-Atgl cff
methylsuccinate	4.8737	0.0048057	0.0094055	PPAR-a-Atgl cff; PPAR-a-Cpt2 cff; PPAR-a-ff:ff Cre
leucine	4.8146	0.0051069	0.0099711	ff:ff Cre-ff:ff; PPAR-a-ff:ff
3-indoxylsulfate	4.8096	0.0051329	0.0099983	ff:ff-Cpt2 cff; ff:ff Cre-ff:ff
78-dihydrofolate	4.7828	0.0052771	0.010255	Cpt2 cff-Atgl cff; ff:ff-Cpt2 cff

sebacateC10-DC	4.7617	0.0053932	0.010456	PPAR-a-Atgl cff; PPAR-a-ff:ff; PPAR-a-ff:ff Cre
thiaminmonophosphate	4.7339	0.0055508	0.010736	Cpt2 cff-Atgl cff; ff:ff Cre-Atgl cff; PPAR-a-Atgl cff
N-arachidoyl-sphingosined181/200	4.7086	0.0056988	0.010996	PPAR-a-Atgl cff; PPAR-a-Cpt2 cff; PPAR-a-ff:ff
beta-hydroxyisovalerate	4.6832	0.0058507	0.011263	PPAR-a-Cpt2 cff; PPAR-a-ff:ff Cre
pyridoxalphosphate	4.6568	0.0060138	0.01155	ff:ff-Atgl cff; ff:ff Cre-ff:ff; PPAR-a-ff:ff
p-cresolsulfate	4.6395	0.0061233	0.011733	PPAR-a-ff:ff
1-palmitoyl-2-linoleoyl-GPE160/182	4.6342	0.0061575	0.011771	Cpt2 cff-Atgl cff; PPAR-a-Atgl cff
alpha-ketoglutaramate	4.6219	0.0062369	0.011891	PPAR-a-ff:ff Cre
betaine	4.62	0.0062493	0.011891	ff:ff Cre-Atgl cff; ff:ff Cre-ff:ff
urea	4.6081	0.0063279	0.012013	PPAR-a-Atgl cff; PPAR-a-ff:ff
malonate	4.5964	0.006406	0.012133	PPAR-a-ff:ff Cre
palmitoleate161n7	4.5869	0.0064697	0.012225	ff:ff-Cpt2 cff; ff:ff Cre-ff:ff
N-2-hydroxypalmitoyl-sphingosined181/1602OH	4.5765	0.0065405	0.012331	PPAR-a-Atgl cff; PPAR-a-Cpt2 cff; PPAR-a-ff:ff Cre
genistein	4.5528	0.0067057	0.012613	PPAR-a-Atgl cff; PPAR-a-Cpt2 cff; PPAR-a-ff:ff; PPAR-a-ff:ff Cre
valine	4.5448	0.0067618	0.01269	ff:ff Cre-ff:ff; PPAR-a-ff:ff
hydroxy-N6N6N6-trimethyllysine	4.5393	0.0068012	0.012735	PPAR-a-Atgl cff; PPAR-a-Cpt2 cff; PPAR-a-ff:ff Cre
methylglucopyranosidealphabeta	4.4971	0.0071105	0.013284	ff:ff Cre-ff:ff
N1-Methyl-2-pyridone-5-carboxamide	4.4443	0.0075182	0.014004	ff:ff Cre-ff:ff; PPAR-a-ff:ff Cre
3-hydroxyoleoylcarnitine	4.44	0.0075529	0.014004	ff:ff-Atgl cff
orotate	4.439	0.0075609	0.014004	Cpt2 cff-Atgl cff; ff:ff-Cpt2 cff
23-dihydroxyisovalerate	4.4386	0.007564	0.014004	ff:ff Cre-ff:ff
oleoylethanolamide	4.4355	0.0075884	0.014017	PPAR-a-ff:ff
heptanoylglycine	4.4335	0.0076051	0.014017	PPAR-a-ff:ff; PPAR-a-ff:ff Cre
palmitoyl-myristoyl-glycerol160/1402	4.4298	0.0076342	0.014039	ff:ff Cre-Atgl cff; ff:ff Cre-ff:ff
palmitoylethanolamide	4.4078	0.0078149	0.014339	PPAR-a-Cpt2 cff; PPAR-a-ff:ff
carnitine	4.4005	0.0078755	0.014418	PPAR-a-Cpt2 cff; PPAR-a-ff:ff
adenosine35-cyclicmonophosphatecAMP	4.3897	0.0079661	0.014551	ff:ff-Cpt2 cff; ff:ff Cre-ff:ff
dCMP	4.3665	0.0081658	0.014883	ff:ff-Cpt2 cff; PPAR-a-Cpt2 cff
xanthine	4.3476	0.0083314	0.015151	ff:ff-Cpt2 cff
CDP-choline	4.3234	0.0085497	0.015514	ff:ff Cre-Atgl cff; PPAR-a-ff:ff Cre
12-HEPE	4.3148	0.0086283	0.015612	PPAR-a-Cpt2 cff; PPAR-a-ff:ff
pyruvate	4.3134	0.0086417	0.015612	ff:ff-Cpt2 cff; PPAR-a-ff:ff
lysine	4.3013	0.0087535	0.015779	ff:ff Cre-Cpt2 cff; PPAR-a-Cpt2 cff
cadaverine	4.2515	0.0092334	0.016574	PPAR-a-Atgl cff; PPAR-a-ff:ff; PPAR-a-ff:ff Cre
gamma-glutamylisoleucine	4.2514	0.0092348	0.016574	Cpt2 cff-Atgl cff; ff:ff Cre-Atgl cff
sphingomyelind182/242	4.223	0.0095204	0.01705	Cpt2 cff-Atgl cff; ff:ff Cre-Atgl cff
4-hydroxy-nonenal-glutathione	4.2095	0.0096598	0.017262	Cpt2 cff-Atgl cff; ff:ff Cre-Atgl cff
glycerophosphoinositol	4.1684	0.010098	0.018006	PPAR-a-ff:ff; PPAR-a-ff:ff Cre
argininosuccinate	4.1548	0.010247	0.018232	Cpt2 cff-Atgl cff; PPAR-a-Cpt2 cff
arachidate200	4.1361	0.010457	0.018565	Cpt2 cff-Atgl cff; ff:ff-Atgl cff; ff:ff Cre-Atgl cff
3-hydroxytetradecanedioate	4.1269	0.010562	0.018711	PPAR-a-Atgl cff; PPAR-a-Cpt2 cff; PPAR-a-ff:ff; PPAR-a-ff:ff Cre
N-acetylhomocitrulline	4.1147	0.010702	0.018919	Cpt2 cff-Atgl cff; ff:ff-Cpt2 cff; ff:ff Cre-Cpt2 cff
1-1-enyl-palmitoyl-2-arachidonoyl-GPEP-160/204	4.0538	0.011434	0.020148	Cpt2 cff-Atgl cff
retinal	4.0528	0.011447	0.020148	PPAR-a-ff:ff
trigonellineN-methylnicotinate	4.0416	0.011587	0.020351	Cpt2 cff-Atgl cff; ff:ff-Cpt2 cff
arachidonate204n6	4.0351	0.01167	0.020453	Cpt2 cff-Atgl cff
glyco-beta-muricholate	4.0276	0.011765	0.020577	ff:ff Cre-Atgl cff; PPAR-a-Atgl cff
hexadecatrienoate163n3	4.0098	0.011996	0.020935	PPAR-a-ff:ff
5-galactosylhydroxy-L-lysine	3.991	0.012245	0.021325	PPAR-a-Atgl cff; PPAR-a-ff:ff
palmitoylcarnitineC16	3.9874	0.012293	0.021363	ff:ff-Cpt2 cff; ff:ff Cre-ff:ff; PPAR-a-ff:ff
N-acetylhistidine	3.9848	0.012329	0.02138	PPAR-a-Cpt2 cff; PPAR-a-ff:ff Cre
proline	3.9815	0.012373	0.021412	PPAR-a-Atgl cff; PPAR-a-ff:ff
glycosylceramided181/200d161/220	3.9626	0.012632	0.021814	PPAR-a-Atgl cff; PPAR-a-ff:ff
urate	3.9301	0.01309	0.022511	PPAR-a-ff:ff Cre
caproate60	3.9301	0.01309	0.022511	Cpt2 cff-Atgl cff; PPAR-a-Cpt2 cff
myristate140	3.9231	0.013192	0.022638	ff:ff-Cpt2 cff; ff:ff Cre-ff:ff
anthranilate	3.9211	0.01322	0.022639	ff:ff Cre-Atgl cff
palmitate160	3.9128	0.013342	0.022801	ff:ff-Atgl cff
1-palmitoleoyl-GPC161	3.9011	0.013515	0.023048	Cpt2 cff-Atgl cff; ff:ff Cre-Atgl cff
lignoceroylsphingomyelind181/240	3.8881	0.01371	0.023332	Cpt2 cff-Atgl cff
ferulicacid4-sulfate	3.8819	0.013804	0.023443	PPAR-a-Atgl cff; PPAR-a-ff:ff Cre
saccharopine	3.8735	0.013931	0.023611	PPAR-a-Cpt2 cff; PPAR-a-ff:ff Cre
gamma-carboxyglutamate	3.8713	0.013966	0.023622	PPAR-a-Atgl cff; PPAR-a-ff:ff Cre
N-acetylasparagine	3.84	0.014457	0.024402	
anserine	3.8149	0.014863	0.025036	PPAR-a-Cpt2 cff
O-methyltyrosine	3.7787	0.015473	0.026009	PPAR-a-ff:ff
dihomolinolenate203n3or3n6	3.7703	0.015618	0.026199	Cpt2 cff-Atgl cff; ff:ff Cre-Atgl cff; PPAR-a-Atgl cff
lactosyl-N-nervonoyl-sphingosined181/241	3.7486	0.015999	0.026785	Cpt2 cff-Atgl cff
2-deoxyadenosine5-monophosphate	3.7458	0.016048	0.026812	ff:ff-Cpt2 cff
methionine	3.7273	0.016383	0.027316	PPAR-a-ff:ff
3-methylglutarate/2-methylglutarate	3.7199	0.016519	0.027487	PPAR-a-Atgl cff; PPAR-a-ff:ff
isoleucine	3.7074	0.016751	0.027817	ff:ff-Cpt2 cff; PPAR-a-ff:ff
1-stearoyl-2-oleoyl-GPS180/181	3.7046	0.016803	0.027846	PPAR-a-Cpt2 cff
2-O-methyluridine	3.6697	0.017472	0.028897	Cpt2 cff-Atgl cff
N-glycolylneuraminate	3.6584	0.017693	0.029204	PPAR-a-ff:ff Cre
56-dihydrouridine	3.6397	0.018069	0.029765	PPAR-a-Cpt2 cff
deoxycholate	3.5986	0.018923	0.031109	PPAR-a-ff:ff
thymidine	3.577	0.019387	0.031809	ff:ff Cre-Atgl cff; ff:ff Cre-Cpt2 cff
prostaglandinF2alpha	3.5717	0.019503	0.031936	ff:ff-Cpt2 cff
6-ketoprostaglandinF1alpha	3.5583	0.0198	0.032358	ff:ff-Cpt2 cff
N1-acetylspermidine	3.5558	0.019856	0.032385	PPAR-a-Atgl cff; PPAR-a-ff:ff
16or17-methylstearatea190ori190	3.5325	0.020386	0.033183	
hexadecasphingosined161	3.5272	0.020509	0.033316	Cpt2 cff-Atgl cff; PPAR-a-Cpt2 cff
3-hydroxyhexanoylcarnitine1	3.524	0.020583	0.033372	PPAR-a-Atgl cff
dihomolinoleate202n6	3.4946	0.021279	0.034431	ff:ff Cre-Atgl cff
heneicosapentaenoate215n3	3.4622	0.022074	0.035649	Cpt2 cff-Atgl cff; PPAR-a-Atgl cff
maltotetraose	3.4508	0.022363	0.036043	PPAR-a-Cpt2 cff; PPAR-a-ff:ff Cre
23-diphosphoglycerate	3.3798	0.024245	0.039001	Cpt2 cff-Atgl cff; PPAR-a-Cpt2 cff
taurohyocholate	3.3759	0.024355	0.039101	ff:ff Cre-Atgl cff; PPAR-a-Atgl cff
N-acetylarginine	3.368	0.024576	0.039359	
CMP	3.3667	0.024611	0.039359	
orotidine	3.3632	0.02471	0.03944	
1-palmitoyl-2-oleoyl-GPC160/181	3.3409	0.025349	0.040381	ff:ff Cre-ff:ff
docosadienoate222n6	3.3147	0.026121	0.04153	ff:ff Cre-Atgl cff
6-beta-hydroxylithocholate	3.2968	0.026662	0.042309	PPAR-a-ff:ff
12-dipalmitoyl-GPE160/160	3.2875	0.026949	0.042682	ff:ff Cre-Atgl cff
nisinate246n3	3.2503	0.028128	0.044464	Cpt2 cff-Atgl cff; PPAR-a-Atgl cff
2-deoxyuridine	3.2391	0.028493	0.044955	ff:ff Cre-ff:ff
N-alpha-acetylornithine	3.2275	0.028875	0.04547	PPAR-a-ff:ff Cre
2-hydroxyphenylacetate	3.2239	0.028997	0.045574	PPAR-a-ff:ff Cre
3-dehydrocholate	3.2049	0.029639	0.046494	PPAR-a-ff:ff Cre
behenate220	3.2026	0.029717	0.046529	
tauro-beta-muricholate	3.1964	0.029933	0.046778	ff:ff Cre-Atgl cff
5-dodecenoate121n7	3.1939	0.03002	0.046825	
3-hydroxysebacate	3.1889	0.030192	0.047003	PPAR-a-Atgl cff
5-oxoproline	3.1849	0.030333	0.047034	Cpt2 cff-Atgl cff; ff:ff Cre-Atgl cff

2-butenoylglycine	3.1848	0.030335	0.047034	ff:ff-Cpt2 cff; ff:ff Cre-ff:ff
N-tetracosadienoyl-sphingosined181/242	3.1835	0.030383	0.047034	PPAR-a-ff:ff
3-hydroxyoctanoate	3.1593	0.031246	0.048207	
histidylalanine	3.159	0.031258	0.048207	
1-carboxyethylisoleucine	3.1504	0.03157	0.048597	PPAR-a-Atgl cff
N2-acetylN6-methyllysine	3.1321	0.032247	0.049547	PPAR-a-ff:ff Cre
alanine	3.1303	0.032315	0.049558	PPAR-a-ff:ff
12-HHTrE	3.1277	0.032413	0.049615	ff:ff-Cpt2 cff
tigloylglycine	3.1234	0.032574	0.049768	
1-1-enyl-palmitoyl-2-oleoyl-GPEP-160/181	3.1196	0.03272	0.049899	PPAR-a-Atgl cff

Supplemental Table 1.

GENE name	Forward primers	Reverse primers	
Pnpla2/Atgl	TGTGGCCTCATTCCTCCTAC	TCGTGGGATGTTGGTGGAGCT	
Acot1	GACAAGAAGAGCTTCATTCCCGTG	CATCAGCATAGAACTCGCTCTTCC	
Acot2	AGTCAACGACGCAAAATGGTG	GCTCTTCCAATCCTGTTGGC	
Cpt2	CAACTCGTATACCCAAACCCAGTC	GTTCCCATCTTGATCGAGGACATC	
Hadh	TGCATTTGCCGCAGCTTTAC	GTTGGCCCAGATTTCGTTCA	
Lcad	TTTCCTCGGAGCATGACATTTT	GCCAGCTTTTTCCCAGACCT	
Fgf21	CTGCTGGGGGTCTACCAAG	CTGCGCCTACCACTGTTCC	
Acox1	ACGCCACTTCCTTGCTCTTC	AGATTGGTAGAAATTGCTGCAAA	
Cpt1b	GGTCCCATAAGAAACAAGACCTCC	CAGAAAGTACCTCAGCCAGGAAAG	
Phospho1	AAGCACATCATCCACAGTCCCTC	TTGGTCTCCAGCTGTCATCCAG	
Elovl7	CATCGAGGACTGTGCGTTTTT	CCAGGATGATGGTTTGTGGCA	
fabp3	ACCTGGAAGCTAGTGGACAG	TGATGGTAGTAGGCTTGGTCAT	
plin4	GACAACTGAGGAACAAGCTCAG	TCCATGGTCATGTCTGTCATCT	PMID: 24820416
Atf3	CGAAGACTGGAGCAAAATGAT	CAGGTTAGCAAAATCCTCAAAC	
gnmbp	CATTCCCATCTCGAAGGTGAAA	AAATGGCAGAGTCGTTGAGGA	
Myc	ATGCCCCTCAACGTGAACTTG	CGCAACATAGGATGGAGAGCA	
col4a1	GCCAAGTGTGCATGAGAAGA	AGCGGGGTGTGTTAGTTACG	
col5a1	CTCCAACACCTCCAATCCAG	GTCCTCCAATCCCCTCAAAG	
elastin	TGGTATTGGTGGCATCGG	CCTTGGCTTTGACTCCTGTG	
Lumican	GAATGTAACTGCCCCCACAG	GGTCAAGAATGAGCCACTGC	
mmp12	GCTAGAAGCAACTGGGCAAC	ACCGCTTCATCCATCTTGAC	
cd68	TTCTGCTGTGGAAATGCAAG	CAATGATGAGAGGCAGCAAG	
F4/80	TTGTACGTGCAACTCAGGACT	GATCCCAGAGTGTTGATGCAA	
CD11	CTGGATAGCCTTTCTTCTGCTG	GCACACTGTGTCCGAACTCA	
TNFa	ATGCTGGGACAGTGACCTGG	CCTTGATGGTGGTGCATGAG	
Nos2	GTTCTCAGCCCAACAATACAAGA	GTGGACGGGTCGATGTCAC	
Tgfb	ATGTCACGGTTAGGGGCTC	GGCTTGCATACTGTGCTGTATAG	
a-sma	GACGTACAACTGGTATTGTG	TCAGGATCTTCATGAGGTAG	PMID: 29320561
nrpl3	CTCCAACCATTCTCTGACCAG	ACAGATTGAAGTAAGGCCGG	PMID: 29070054
pnpla3	GGATCACAGGATCTGGGCTA	TGCACTCCCTGGTGTGTTTA	PMID: 25060692
gdf15	CGCCCTGGCAATGCCTGAACAACGAC	GCAGCGGGTAGGCTTCGGGGAGACC	
angptl3	AGCAAGACAACAGCATAAGAGAACTC	TGAGCTGCTTTTCTATTTCTTTTATCTG	PMID: 26305978
18S	GCAATTATTCCCCATGAACG	GGCCTCACTAAACCATCCAA	
Ehhadh	ATGGCTGAGTATCTGAGGCTG	GGTCCAAACTAGCTTTCTGGAG	
Pdk4	ATCTAACATCGCCAGAATTAAACC	GGAACGTACACAATGTGGATTG	
ppara	GCGTACGGCAATGGCTTTAT	GAACGGCTTCCTCAGGTTCTT	
Cyclo a	TCCGACTGTGGACAGCTCTA	ATTGCGAGCAGATGGGGTAG	PMID: 29390963

Supplemental Table 2.
